# End-Functionalized Poly(vinylpyrrolidone) for Ligand Display in Lateral Flow Device Test Lines

**DOI:** 10.1021/acspolymersau.1c00032

**Published:** 2021-11-12

**Authors:** Alexander N. Baker, Thomas R. Congdon, Sarah-Jane Richards, Panagiotis G. Georgiou, Marc Walker, Simone Dedola, Robert A. Field, Matthew I. Gibson

**Affiliations:** † Department of Chemistry, University of Warwick, Gibbet Hill Road, CV4 7AL Coventry, U.K.; ‡ Warwick Medical School, University of Warwick, Gibbet Hill Road, CV4 7AL Coventry, U.K.; § Department of Physics, University of Warwick, Gibbet Hill Road, CV4 7AL Coventry, U.K.; ∥ Iceni Diagnostics Ltd, Norwich Research Park, Norwich NR4 7GJ, U.K.; ⊥ Department of Chemistry and Manchester Institute of Biotechnology, University of Manchester, Manchester M1 7DN, U.K.

**Keywords:** lateral flow assay, polymers, RAFT, glycans, diagnostics, biosensing

## Abstract

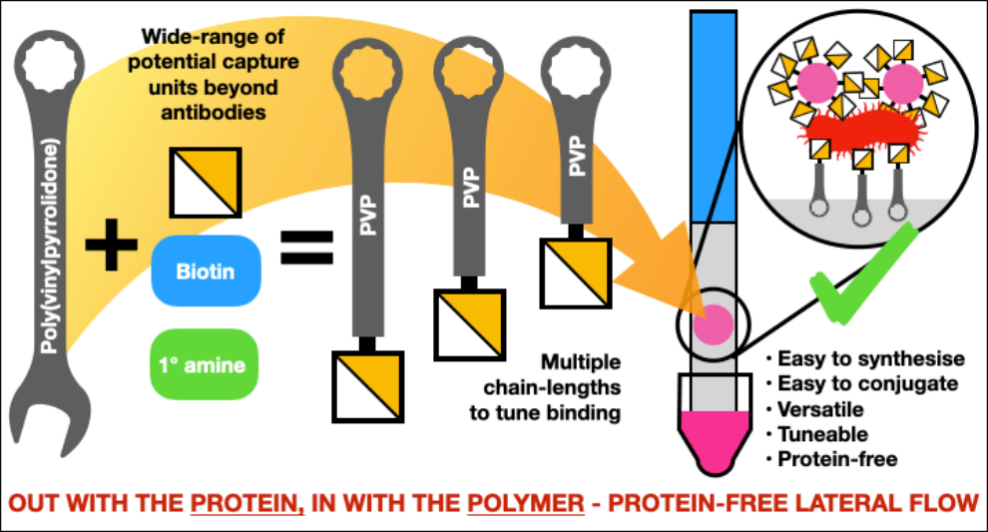

Lateral flow devices are rapid (and often low cost) point-of-care diagnostics—the classic example being the home pregnancy test. A test line (the stationary phase) is typically prepared by the physisorption of an antibody, which binds to analytes/antigens such as viruses, toxins, or hormones. However, there is no intrinsic requirement for the detection unit to be an antibody, and incorporating other ligand classes may bring new functionalities or detection capabilities. To enable other (nonprotein) ligands to be deployed in lateral flow devices, they must be physiosorbed to the stationary phase as a conjugate, which currently would be a high-molecular-weight carrier protein, which requires (challenging) chemoselective modifications and purification. Here, we demonstrate that poly(vinylpyrrolidone), PVP, is a candidate for a polymeric, protein-free test line, owing to its unique balance of water solubility (for printing) and adhesion to the nitrocellulose stationary phase. End-functionalized PVPs were prepared by RAFT polymerization, and the model capture ligands of biotin and galactosamine were installed on PVP and subsequently immobilized on nitrocellulose. This polymeric test line was validated in both flow-through and full lateral flow formats using streptavidin and soybean agglutinin and is the first demonstration of an “all-polymer” approach for installation of capture units. This work illustrates the potential of polymeric scaffolds as anchoring agents for small-molecule capture agents in the next generation of robust and modular lateral flow devices and that macromolecular engineering may provide real benefit.

## Introduction

Lateral flow devices (LFDs) are point-of-care (POC) diagnostics that are suited to primary care, triage, and emergency applications.^1^ The most widely known LFD is the home pregnancy test, which detects the presence of the hormone human chorionic gonadotrophin (HCG) in urine in under 20 min.^2,3^ In these devices, the stationary phase of the LFD is nitrocellulose functionalized with an antibody that binds HCG. Gold nanoparticles (AuNPs) functionalized with the same antibody are in the mobile phase. This leads to the sandwiching of HCG between the immobilized antibody on the device surface and the antibody on the AuNPs, producing an optical signal—often a red line; notably, other signal producing elements can be used such as quantum dots,^4^ graphene oxide,^5,6^ and carbon nanotubes.^7^

LFDs have many applications beyond detecting HCG; for example, they have been deployed for analytes such as drugs of abuse,^8^ Ebola virus,^9^ meningitis,^10^ and SARS-COV-2.^11^ The common design principle shared by the above tests is they all use antibodies as capture agents (lateral flow immunoassays) due to the very high affinity and selectivity of antibodies toward their ligands (in the range of nM to pM). Despite the ubiquity of antibodies in LFDs, there is no functional requirement that these be used as the capture agent. There are examples of LFDs that use protein-anchored nucleotides,^12^ protein-anchored glycans,^13^ and lectins^14^ as capture agents in the mobile phase and as test lines in the stationary phase. There are potential benefits of using alternative ligand capture molecules. For example, Baker et al. have demonstrated that the spike protein from SARS-COV-2 (causative agent of COVID-19) can be detected in a lateral flow/flow-through setup by using *N*-acetyl neuraminic acid (NeuNAc, a glycan) as the recognition agent but required a glycosylated protein as the test line.^13^ The same system could be deployed in flow-through (no test line) to detect COVID-19 positives in primary patient swab samples.^15^

Miura et al. have made hybrid LFDs to detect plant proteins, using glycosylated nanoparticles as the mobile phase but still using an antibody as the test line.^16^ By moving away from (or combining with) antibody-based detection, it may be possible to more rapidly develop new LFDs, by enabling the development of fully synthetic systems removing the need to raise antibodies (in, e.g., animal models). This new approach could allow for easier manufacture (including scaling) as well as bringing additional discriminatory power to tests.

Nearly all current LFDs use antibodies (lateral flow immunoassays) as the stationary phase (as well as the mobile phase) or use proteins that are functionalized with other ligands, such as nucleic acids, in the stationary phase. These approaches lead to three fundamental challenges. First, the molecular weight of the test-line conjugate must be large enough to attach to the surface, with absorption ability decreasing with decreasing molecular weight, limiting scope to very high-molecular-weight macromolecules.^17,18^ This limit can be overcome by increasing the surface area of the stationary phase membrane, although this limits the choice of stationary phase membrane material.^19^ Second, bovine serum albumin (BSA) or other proteins must be used as “anchors” to immobilize small capture agents such as nucleotides or glycans onto the surface of an LFD; this is further limited by the small number of easy-to-use chemical conjugations available to functionalize carrier proteins. Moreover, the chemical conjugation approaches used do not provide a clear picture of the number of capture units per protein. For example, when using glycan-functionalized BSA, a range of degrees of glycosylation are obtained, with the number of glycans differing by glycan used too.^20^ Third, the temperature instability of many protein-based LFDs above 30–40 °C may prevent devices from being deployed in various low-resource settings that lack established health infrastructures and cold chains.^21,22^ This is especially problematic, as more expensive lab-based diagnostic techniques are also not applicable, as exemplified by the COVID-19 crisis, creating a clear health inequality between higher- and lower-income countries.^23^

When considering test-line design, all test lines used in LFDs must be sufficiently hydrophobic to remain immobilized on the surface of the LFD as the mobile phase passes by, but must also be hydrophilic enough to dissolve in water for application to the stationary phase (many organic solvents can damage stationary phase materials). It is also common practice when designing LFDs to use a series of proteins or polymers such as bovine serum albumin, casein, or poly(vinylpyrrolidone) (PVP) as blocking agents (i.e., substances that coat (“block”) the surface of the stationary phase, to prevent nonspecific binding of the mobile phase to the stationary phase).^17,18^ Blocking agents are either applied to the stationary phase as a pretreatment before the LFD is run or contained within the buffer of the LFD and run as a component of the mobile phase. PVP is an interesting case, as it is widely used in LFDs as a blocking agent, is hydrophilic enough to dissolve in water but hydrophobic enough to be immobilized onto nitrocellulose (reflected by vinylpyrrolidone’s logP of ∼0.37),^24^ is widely used in biomedical applications,^25^ and is a synthetic polymer allowing for chemical modification. Therefore, it seemed an ideal candidate to prove the principle that a universal polymeric anchor for LFDs could be discovered.

Herein, we explore the use of capture-agent-functionalized PVP as a test line in flow-through assays, lateral flow assays, and lateral flow glycoassays,^13^ as the first example (to the best of our knowledge) of creating a synthetic polymer test line. The performance of the test line was investigated using biotin-functionalized PVP with streptavidin-functionalized AuNPs (as the mobile phase) in a flow-through assay as well as free streptavidin and biotin-functionalized AuNPs in a lateral flow assay. Further exemplification is provided using glycosylated PVP to detect a lectin in a lateral flow glycoassay. Crucially, the polymer molecular weight can be tuned to impact the final output, providing a unique fine-tuning tool, not possible with current technologies. The polymer approach is also highly modular, as shown here. This new approach to immobilizing ligands onto the test line will help develop the next generation of LFDs and simplify workflows.

## Results and Discussion

The primary aim of this work was to synthesize and test the first generation of fully synthetic, protein-free test lines for use in LFD devices, to facilitate the development pipeline of new LFDs, using robust polymeric anchoring agents. Poly(vinylpyrrolidone), PVP, was chosen as the polymeric anchor, as it is widely used in LFDs as a blocking agent—it is flowed over the nitrocellulose stationary phase to reduce nonspecific interactions of analytes or media components. Hence, if it is blocking nonspecific binding, we reasoned that PVP must be sufficiently hydrophobic to interact/coat the nitrocellulose while also being hydrophilic enough to dissolve in water,^26,27^ which is an essential criterion for test-line printing from aqueous solution.

**Figure 1:**
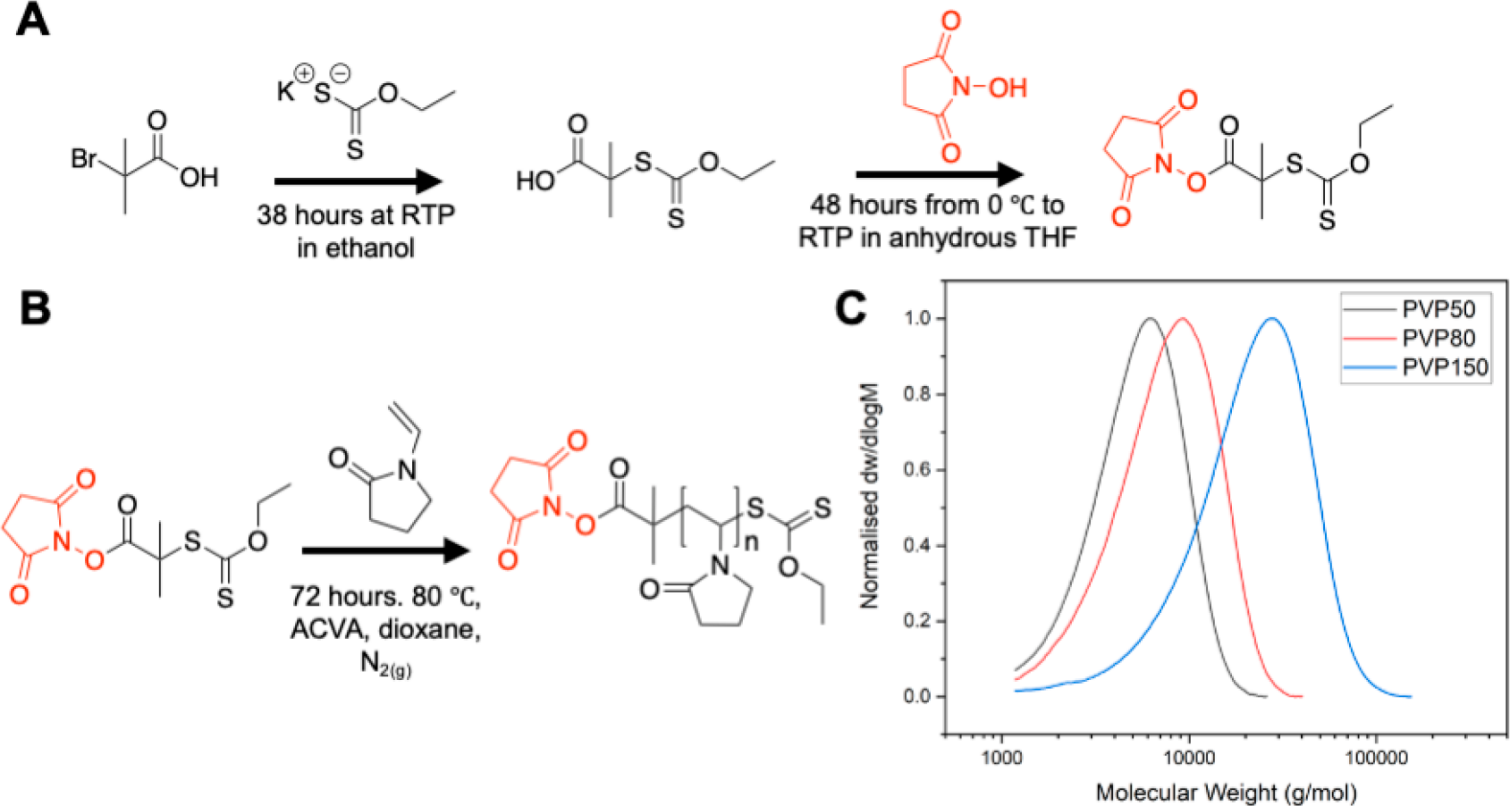
Polymer synthesis. (A) Synthesis of MADIX chain-transfer agent (CTA); (B) polymerization of *N*-vinylpyrrolidone (NVP); (C) normalized molecular weight distributions from size exclusion chromatography of PVP polymers from Table 1.

Reversible addition–fragmentation chain-transfer (RAFT) polymerization was employed, as it enables the synthesis of polymers with defined chain lengths and control over end-groups (crucial to add the binding ligand of interest). Furthermore, RAFT or MADIX (macromolecular design by the interchange of xanthates, a specific type of RAFT) is compatible with less-activated monomers (LAMs) such as NVP (*N*-vinylpyrrolidone) or VAc (vinyl acetate), which are more challenging than, for example, (meth)acrylates to polymerize.^28−30^ A xanthate chain-transfer agent (CTA) of 2-(ethoxycarbonothioylthio)-2-methylpropanoic acid *N*-hydroxysuccinimide ester was designed^31^ and synthesized with a *N*-hydroxysuccinimide (NHS) end-group that could be substituted by primary amines as shown in Figure 1A. Displacement of the NHS end-group could also be tracked using ^1^H NMR analysis.

Three chain lengths of PVP telechelic homopolymers (DP = 50, 80, and 150) were synthesized (as determined by ^1^H NMR end-group analysis) via thermally initiated RAFT polymerization using 4,4′-azobis (4-cyanovaleric acid) (ACVA) as a radical initiator (Figure 1B). Due to low conversions, which are typically observed in the polymer synthesis of LAMs,^32^ monomer to CTA ratios were higher than the target DPs ([M]:[CTA] = 200, 300, and 500) of 50, 80, and 150, respectively. Polymerization was also stopped at less than 100% conversion to maximize the retention of end-groups. Size exclusion chromatography (SEC) analysis in DMF with 5 mM NH_4_BF_4_ revealed monomodal molecular weight distribution peaks with relatively low dispersities (*Đ*_M_ ≤ 1.7) (Figure 1C and Table 1).

**Table 1:** PVP Polymers Prepared for the Detection of Streptavidin

polymer	[M]:[CTA]	*M*_n(target)_ (g mol^–1^)^a^	*M*_n(SEC)_ (g mol^–1^)^b^	*M*_n(NMR)_ (g mol^–1^)^c^	*Đ*_M_^b^
PVP_50_	200	22 500	4500	5900	1.33
PVP_80_	300	33 600	6000	9200	1.47
PVP_150_	500	55 900	15 100	17 000	1.72

a Determined from the feed ratio of the monomer to chain-transfer agent assuming 100% conversion.

b Calculated against poly(methyl methacrylate) standards using 5 mM NH_4_BF_4_ in DMF as an eluent.

c Determined from ^1^H NMR end-group analysis by comparing the integrations of the −CH_2_ signals (δ 2.8 ppm) of the NHS end-group with those of the corresponding signals of the polymer.

**Figure 2:**
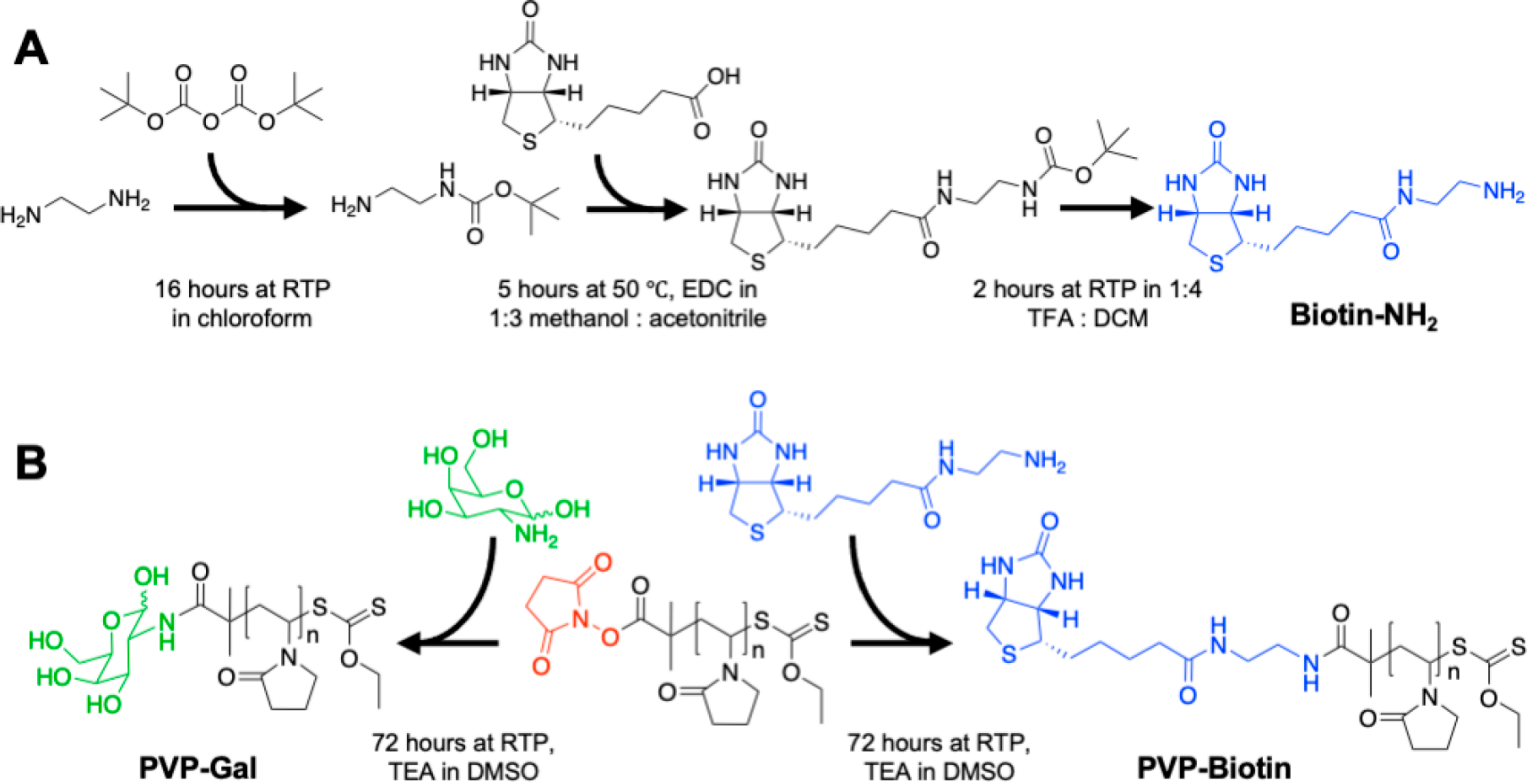
Synthesis of biotin-functionalized and galactosamine-functionalized PVP polymers. (A) Synthesis of biotin derivative; (B) synthesis of biotin-functionalized PVP polymers and galactosamine-functionalized PVP polymers.

To determine if PVP provided a suitable anchor, a model flow-through system was devised using a biotin-end-group, which has well-characterized and strong binding to streptavidin to test the capture principle. [Note, flow-through is distinct from full lateral flow, which has analyte and functionalized gold particles in the mobile phase, which is evaluated in full later.] An amino-biotin derivative was synthesized in three steps from ethylenediamine and biotin following procedures from Eisenführ et al.^33^ and Kaufman et al. (Figure 2A).^34^ A mono-*t*-Boc-protected diamine was synthesized (*N*-Boc-ethylenediamine) and conjugated with biotin. The Boc protecting group was then removed using TFA to produce “biotin-NH_2_”, a biotin derivative with amine functionality.^34^ The biotin-NH_2_ was characterized by ^1^H and ^13^C NMR, FTIR, and ESI mass spectrometry (Supporting Information). Functionalization of the PVP polymers (Figure 2B) was confirmed by the loss of the NHS protons in the ^1^H NMR spectra and the addition of biotin-NH_2_ protons.

**Figure 3:**
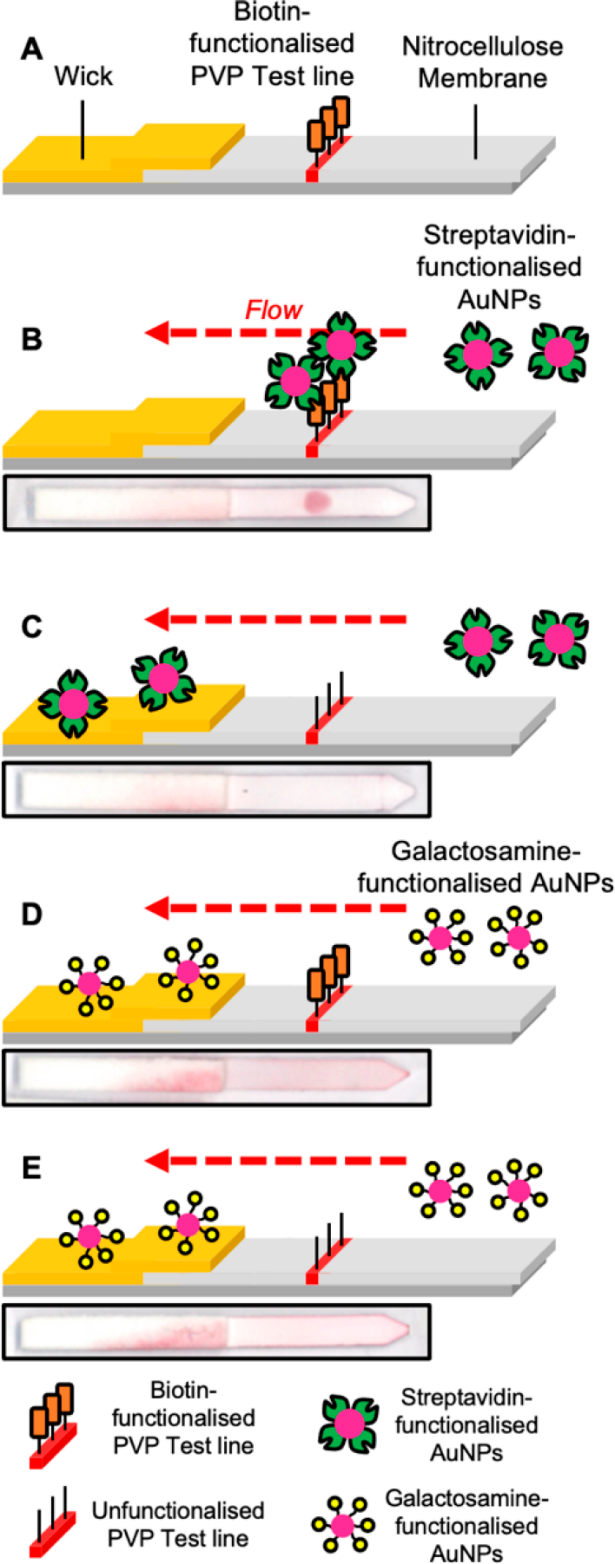
Schematic of dipstick flow-through assay and example dipsticks. (A) Design of dipstick; (B) flow-through with biotin-functionalized PVP test line where streptavidin-functionalized AuNPs flow and engage the test line, resulting in signal generation; C) flow-through with unfunctionalized PVP test line where streptavidin-functionalized AuNPs flow and do not engage the test line, resulting in no signal generation; (D) flow-through with biotin-functionalized PVP test line where Gal-functionalized AuNPs flow and do not engage the test line, resulting in no signal generation; (E) flow-through with unfunctionalized PVP test line where Gal-functionalized AuNPs flow and do not engage the test line, resulting in no signal generation.

The biotin-functionalized PVP and an unfunctionalized PVP control were deposited onto the nitrocellulose dipsticks as test lines, in triplicate, of varying concentrations in water (20, 10, and 1 mg·mL^–1^) and then dried at 37 °C. It is noteworthy that all dipsticks run in this work were run in triplicate, image analyzed, and the average (mean) taken. A (commercial) gold nanoparticle (AuNP, 40 nm) functionalized with streptavidin was flowed down the surface of the dipstick to determine if the biotin-functionalized PVP sequestered the streptavidin-functionalized particles. As a negative mobile phase, a previously reported galactosamine-functionalized poly(hydroxyethyl acrylamide) (PHEA_72_) gold nanoparticle (16 nm) system (Gal–PHEA_72_@AuNP_16_) was used, which has no affinity to biotin (Figure 3).^13^

**Figure 4:**
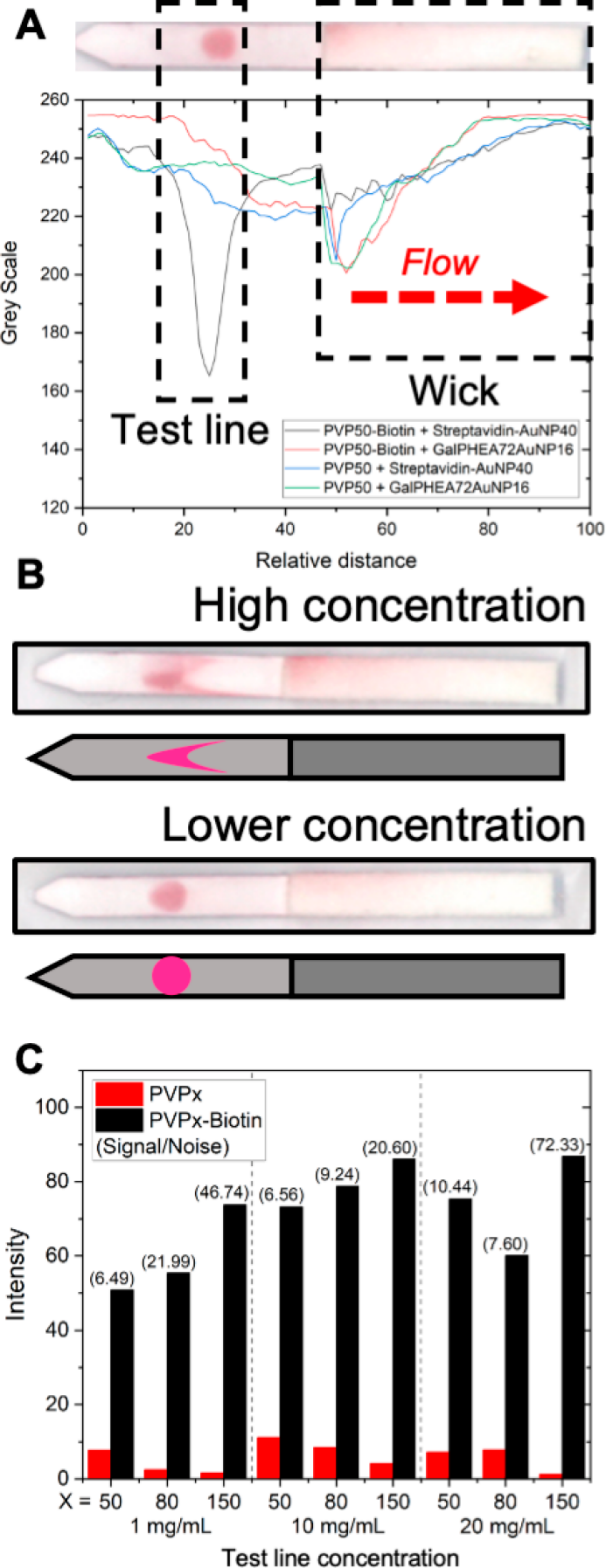
Analysis of flow-through dipstick assays. (A) Analysis of PVP_50_–biotin and unfunctionalized PVP_50_ (1 mg·mL^–1^) versus streptavidin-functionalized AuNPs and galactosamine-functionalized AuNPs, with example dipstick of PVP_50_–biotin versus streptavidin–AuNP_40_; (B) representative example dipsticks and graphical representation of test-line “bleeding” effect at high (top, 20 mg·mL^–1^) and lower test-line concentrations (bottom, 1 mg·mL^–1^); (C) intensity of PVP_*x*_ and PVP_*x*_–biotin at varying concentrations versus streptavidin-functionalized AuNP_40_ (signal-to-noise ratio (PVP_*x*_–biotin intensity/PVP_*x*_ intensity) is provided in brackets).

All dipsticks that used a test line of biotin-functionalized PVPs successfully bound the streptavidin AuNPs at all concentrations of the applied test line. Example dipsticks and the surface image analysis are provided in Figure 4A. Images of all dipsticks and analysis are provided in the Supporting Information (Tables S4–S6 and Figures S23–S25). No nonspecific binding was observed to any of the triplicate controls at 20 mg·mL^–1^ (except perhaps weak binding in one PVP_50_ test strip to the streptavidin–AuNP_40_), although a “bleeding” effect (smearing of the test spot) was observed at higher test-line concentrations (10 and 20 mg·mL^–1^), indicating that the test-line concentration impacts binding and likely saturates the nitrocellulose membrane (Figure 4B). Interestingly the best polymer system, i.e., the system that provided the highest signal response, varied by concentration of test line applied, although all gave a positive signal in all triplicates run, with no observable off-target binding to the unfunctionalized PVP test line seen in the 10 or 1 mg·mL^–1^ triplicates. This was first determined visually and then measured by digitally analyzing the image (Figure 4A) and signal-to-noise ratios determined (Figure 4C). The PVP_80_–biotin system had the highest signal response at 10 mg·mL^–1^ but the lowest at 20 mg·mL^–1^, while the PVP_50_–biotin system had the highest response at just 1 mg·mL^–1^, while PVP_80_–biotin and PVP_150_–biotin were comparable. This indicates that the systems produced require tuning to find the correct test line and concentration for the application; this additional tunability gained from varying polymer chain length is another benefit of the polymeric system versus protein-based systems.

**Figure 5:**
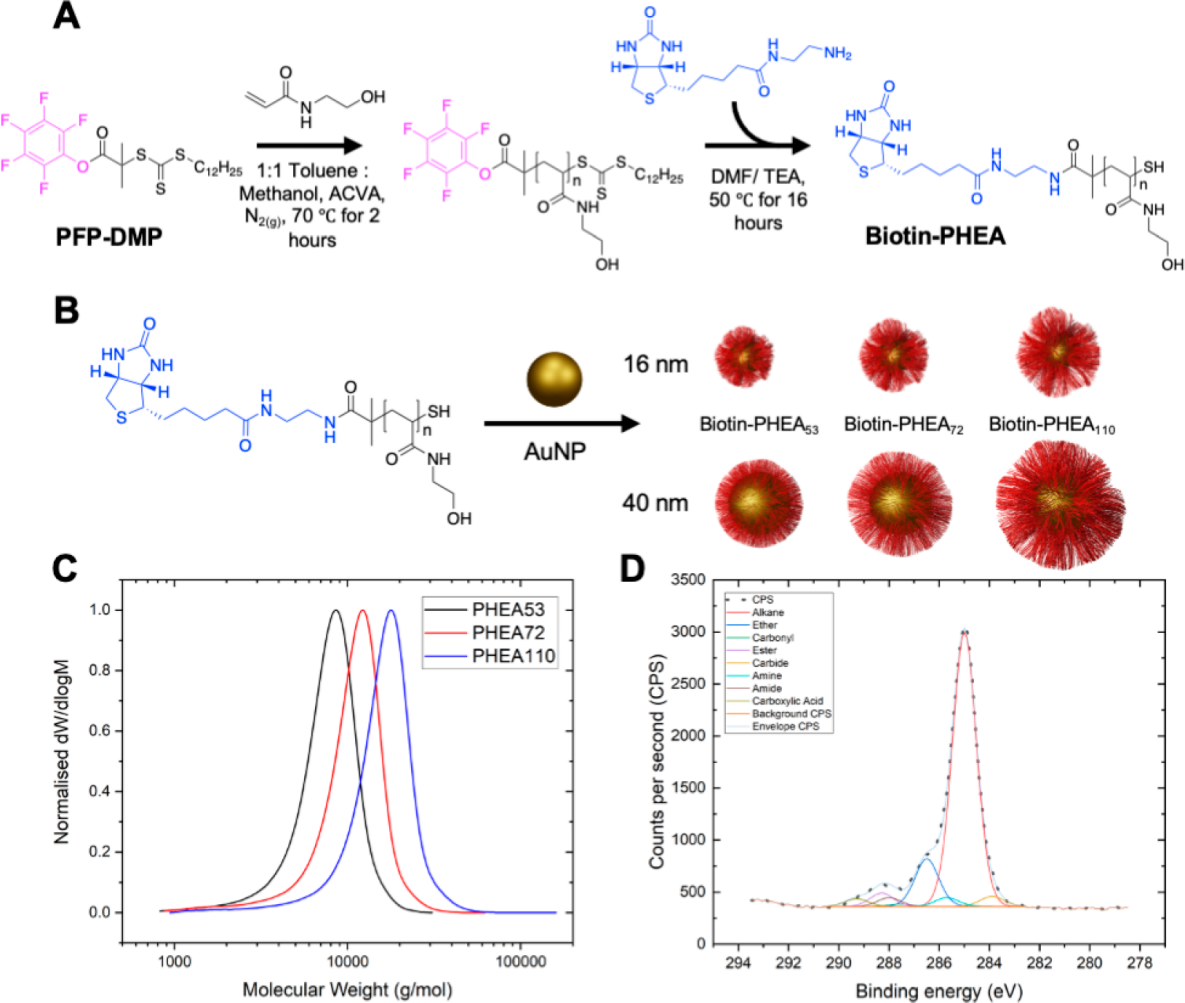
Synthesis of PHEA polymers and AuNPs. (A) Polymerization of *N*-hydroxyethyl acrylamide (HEA) and postfunctionalization with a biotin derivative; (B) synthesis of polymer-functionalized AuNPs; (C) normalized size exclusion chromatography analysis of PHEA polymers from Table 2; (D) C 1s XPS scan of biotin–PHEA_72_@AuNP_40._.

Following the successful demonstration of a flow-through system with biotin-functionalized PVPs as a test line, the next step was to create a lateral flow setup that sensed for free streptavidin in solution, which requires biotin-functionalized AuNPs, coated with a noninteracting water-soluble polymer. Therefore, a series of biotin-functionalized poly(*N*-hydroxyethyl acrylamide)s (PHEA) were synthesized and immobilized on 16 and 40 nm gold nanoparticles (Figure 5A,B). PHEA was chosen because of its colloidal stability,^35−37^ solubility, and its established use to functionalize gold nanoparticles for lateral flow and flow-through devices.^13^ Using a pentafluorophenyl-2-dodecylthiocarbonothioylthio)-2-methylpropanoate (PFP-DMP) chain-transfer agent (CTA) (see Supporting Information for a detailed synthetic procedure), a series of PHEA homopolymers were prepared (DP = 53, 72, 110, as determined by SEC, Figure 5C and Table 2) according to a previously described protocol.^13^ Biotin installation as the end-groups of PHEA homopolymers was achieved by the reaction of the pentafluorophenol (PFP) end-group at the α-terminus with biotin-NH_2_. The functionalized polymers were characterized by ^1^H and ^19^F NMR and FTIR with successful conjugation of biotin-NH_2_ confirmed by loss of the PFP fluorine peaks in ^19^F NMR. The gold nanoparticles produced were characterized by UV–vis, DLS (Supporting Information Figures S13–S22 and Table S3), and X-ray photoelectron spectroscopy (XPS) (Figure 5D and Supporting Information Figures S35–S43 and Tables S20 and S21). The increase in the N 1s/C 1s ratios in the XPS spectra for the polymer-coated particles and the increased presence of amine and amide in the C 1s spectra compared to the citrate-stabilized (“naked”) nanoparticles confirmed the presence of polymers on the nanoparticles, alongside a shift in the UV–vis spectra. The library-based design approach to synthesizing AuNP systems for lateral flow and flow-through assays has been established by Baker et al.^13^ as a method to find the appropriately sized polymer-coated gold particle for the intended diagnostic application.

**Table 2:** PHEA Polymers Prepared for the Detection of Streptavidin

polymer	[M]:[CTA]	*M*_n(target)_ (g mol^–1^)^a^	*M*_n(SEC)_ (g mol^–1^)^b^	*M*_n(NMR)_ (g mol^–1^)^c^	*Đ*_M_^b^
PHEA_53_	28	3800	6600	6000	1.24
PHEA_72_	40	5100	8900	8600	1.28
PHEA_110_	70	8600	13 000	14 000	1.27

a Determined from the feed ratio of the monomer to chain-transfer agent.

b Calculated against poly(methyl methacrylate) standards using 5 mM NH_4_BF_4_ in DMF as an eluent.

c Determined from ^1^H NMR end-group analysis by comparing the integrations of the −CH_3_ signals (δ 0.92 ppm) of the dodecyl end-group with those of the corresponding signals of the polymer.

The DLS (dynamic light scattering) analysis of the biotin-functionalized 16 nm gold particles indicated some aggregation at all polymer lengths. This was observed in the dipsticks, run in triplicate, where the particles aggregated at the solvent front and on the PVP test lines even when no analyte and off-target protein (UEA, *Ulex europaeus* agglutinin I) at 0.05 mg·mL^–1^ was present (Supporting Information Tables S7–S9). However, greater aggregation at the solvent front was observed in systems containing streptavidin, indicating affinity toward streptavidin; this was observed visually by more intense coloration at the solvent front, decreased background along the strips, and decreased coloration in the wick—indicating that fewer AuNPs passed the solvent front. Note, a PVP test concentration of 10 mg·mL^–1^ was chosen to decrease the bleeding effect observed in the flow-through devices.

**Figure 6:**
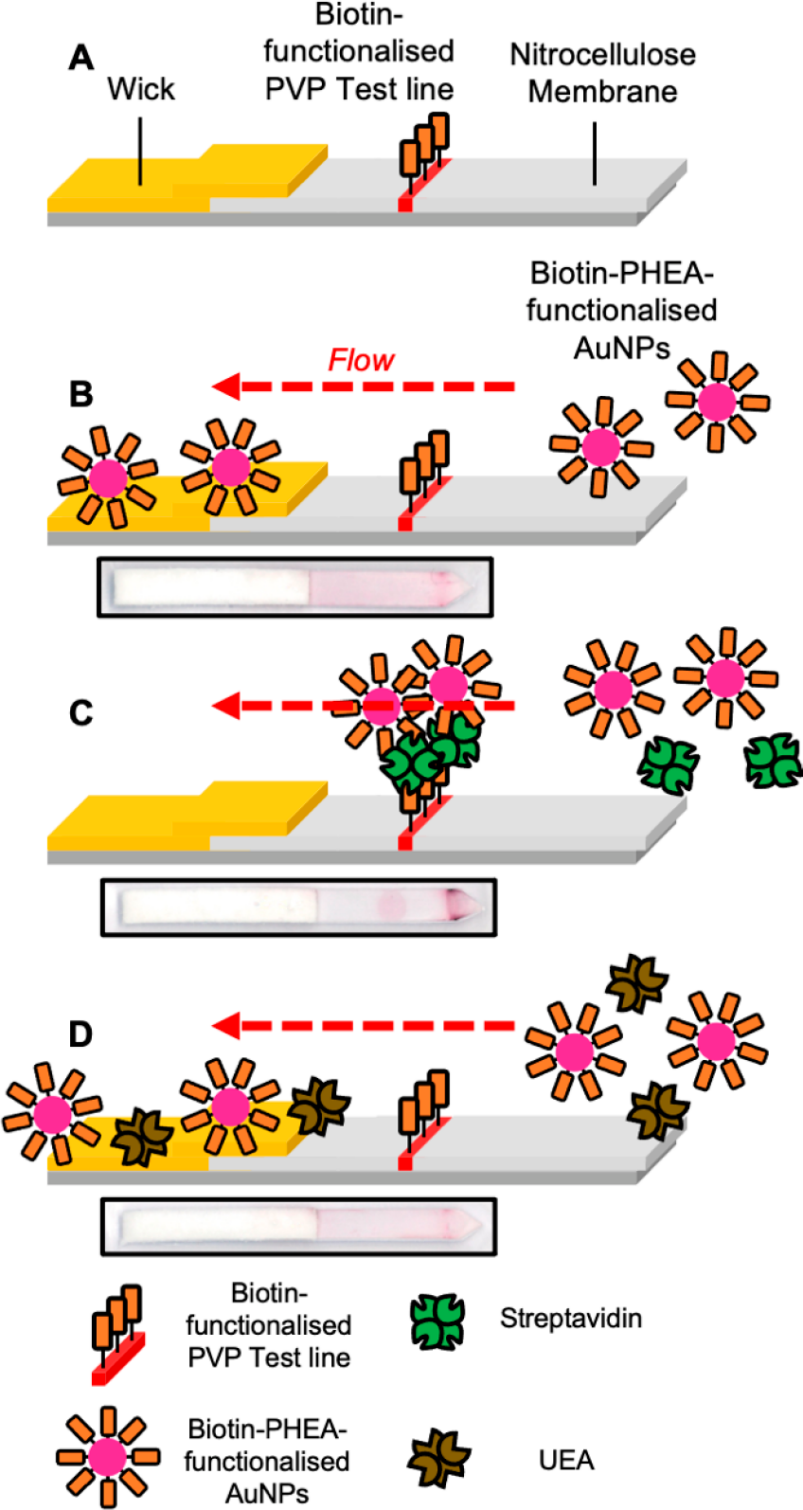
Schematic of dipstick lateral flow assay and example dipsticks. (A) Design of dipstick; (B) lateral flow with biotin-functionalized PVP test line with no analyte in solution, and biotin–PHEA-functionalized AuNPs flow and do not engage the test line, resulting in no signal generation; (C) lateral flow with biotin-functionalized PVP test line with streptavidin (0.05 mg·mL^–1^) in solution, and biotin-functionalized AuNPs flow and do engage the test line, resulting in signal generation; (D) lateral flow with biotin-functionalized PVP test line with UEA (0.05 mg·mL^–1^) in solution, and biotin-functionalized AuNPs flow and do engage the test line, resulting in no signal generation.

**Figure 7:**
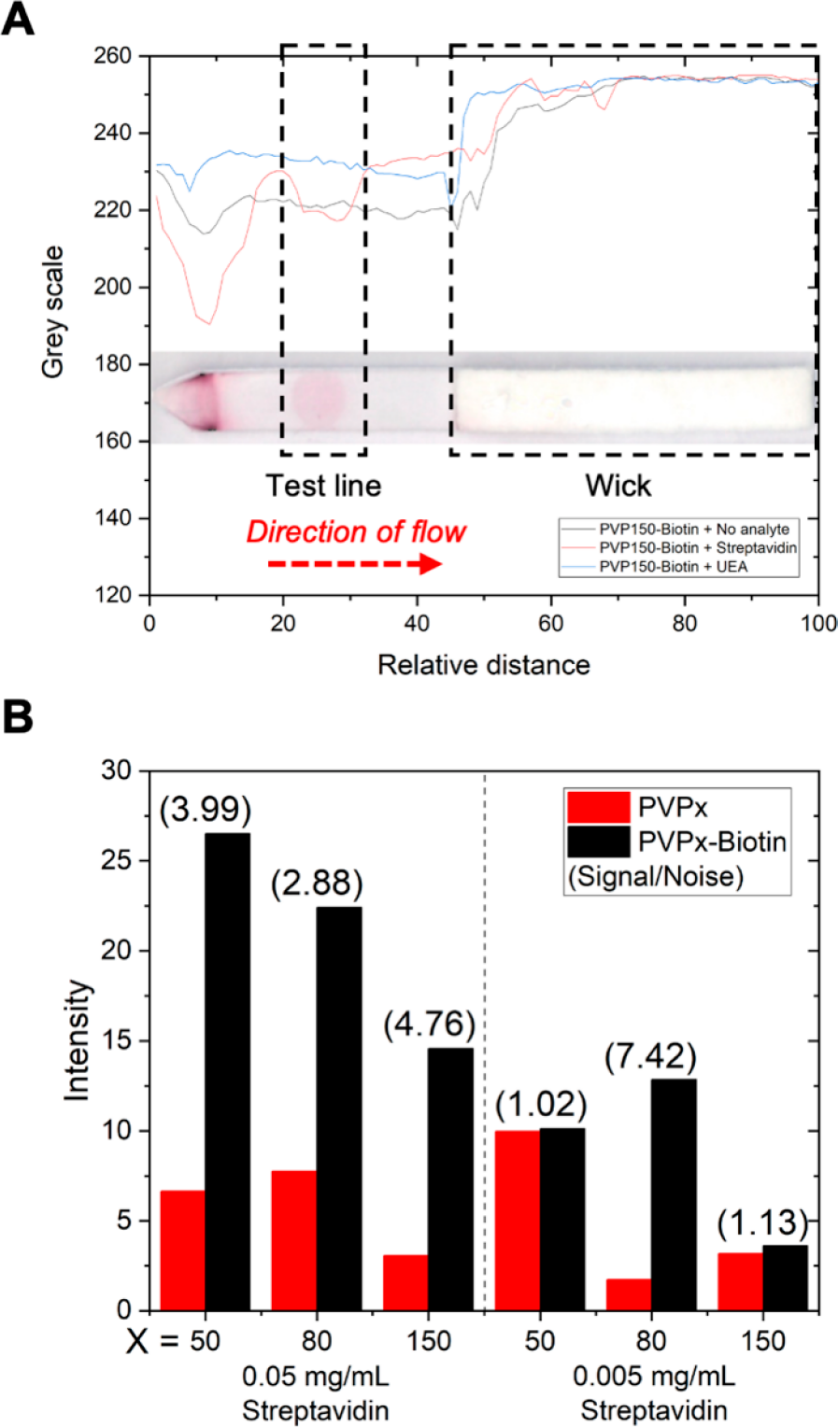
Analysis of scanned lateral flow strips using test lines of PVP_150_–biotin. (A) PVP_150_–biotin (1 mg·mL^–1^) versus either no analyte, streptavidin (0.05 mg·mL^–1^), or UEA (0.05 mg·mL^–1^) used with biotin–PHEA_110_@AuNP_40_ (inset example dipstick from PVP_150_–biotin versus streptavidin); (B) intensity of PVP_*x*_ (1 mg·mL^–1^) and PVP_*x*_–biotin (1 mg·mL^–1^) versus streptavidin of varying concentrations using with biotin–PHEA_110_@AuNP_40_ (signal-to-noise ratio (PVP_*x*_–biotin intensity/PVP_*x*_ intensity) is provided in brackets).

The biotin–PHEA-functionalized 40 nm gold particles were more stable in solution than the 16 nm particles. However, aggregation at the solvent front and with streptavidin at the solvent front was observed in the biotin–PHEA_72_@AuNP_40_ system but less so in the biotin–PHEA_110_@AuNP_40_ system (Supporting Information Tables S10–12 and Figures S26–28). Furthermore, off-target binding to the 10 mg·mL^–1^ PVP–biotin test lines was observed in all biotin–PHEA_110_@AuNP_40_ systems. Hence, the concentration of the test line was decreased to 1 mg·mL^–1^. At this concentration, the biotin–PHEA_110_@AuNP_40_ system bound to streptavidin at a protein concentration of 0.05 mg·mL^–1^, and this AuNP–analyte complex was successfully bound by the PVP_150_–biotin test line (in all triplicates) with minimal nonspecific binding observed in the UEA or no analyte system (Figures 6 and 7, Supporting Information Table 3 and Figure S29). Notably, aggregation of the AuNP system with streptavidin was observed at the solvent front likely reducing signal and leading to increased background in the controls. This experiment confirmed that functionalized PVP test lines could be used successfully in LFDs.

To confirm it is the biotin that the streptavidin specifically binds in the test lines; streptavidin at 0.05 mg·mL^–1^ with biotin–PHEA_110_@AuNP_40_ particles was tested against biotin-functionalized and unfunctionalized PVP test lines at a test-line concentration of 1 mg·mL^–1^ (Supporting Information Table S14 and S30). While weak binding was observed to the unfunctionalized PVP_50_ test line, binding was far stronger to the PVP_50_–biotin test line and all other biotin-functionalized test lines versus their unfunctionalized equivalents, with no binding to the unfunctionalized PVP_150_ test line observed in any of the triplicates. It is notable that signal intensity decreased with PVP chain length—likely because relative biotin concentration on the test line decreases as polymer chain length increases (as test-line concentration is measured by mass not molarity), although the decrease in off-target binding to unfunctionalized PVP_150_ led to a high signal-to-noise ratio for the PVP_150_–biotin system (Figure 7B). Attempts to use a lower concentration of streptavidin (0.005 mg·mL^–1^) and the PVP_150_–biotin test line were unsuccessful, with a signal-to-noise ratio of ∼1. However, binding to the PVP_80_–biotin was observed at this concentration (0.005 mg·mL^–1^ streptavidin) versus unfunctionalized PVP_80_ (signal-to-noise ratio of >7), likely due to decreased aggregation at the solvent front between the particles and streptavidin (Figure 7B, Supporting Information Tables S15 and S31), indicating the need to tune the AuNP system for the target analyte and test line used in a finished device.

In comparison to antibody-based lateral flow immunoassays that often have limits of detection ranging from micrograms to nanograms per milliliter,^38^ this system when targeting streptavidin has a limit of detection (LOD) of ∼0.05–0.005 mg·mL^–1^ or ∼0.8–0.08 nmol·mL^–1^ for the PVP_150_–biotin and PVP_80_–biotin systems. This is higher than many commercially available lateral flow immunoassays but is comparable to commercial pregnancy test LFDs with molar LODs of ∼0.7–0.07 nmol·mL^–1^.^39^

**Figure 8:**
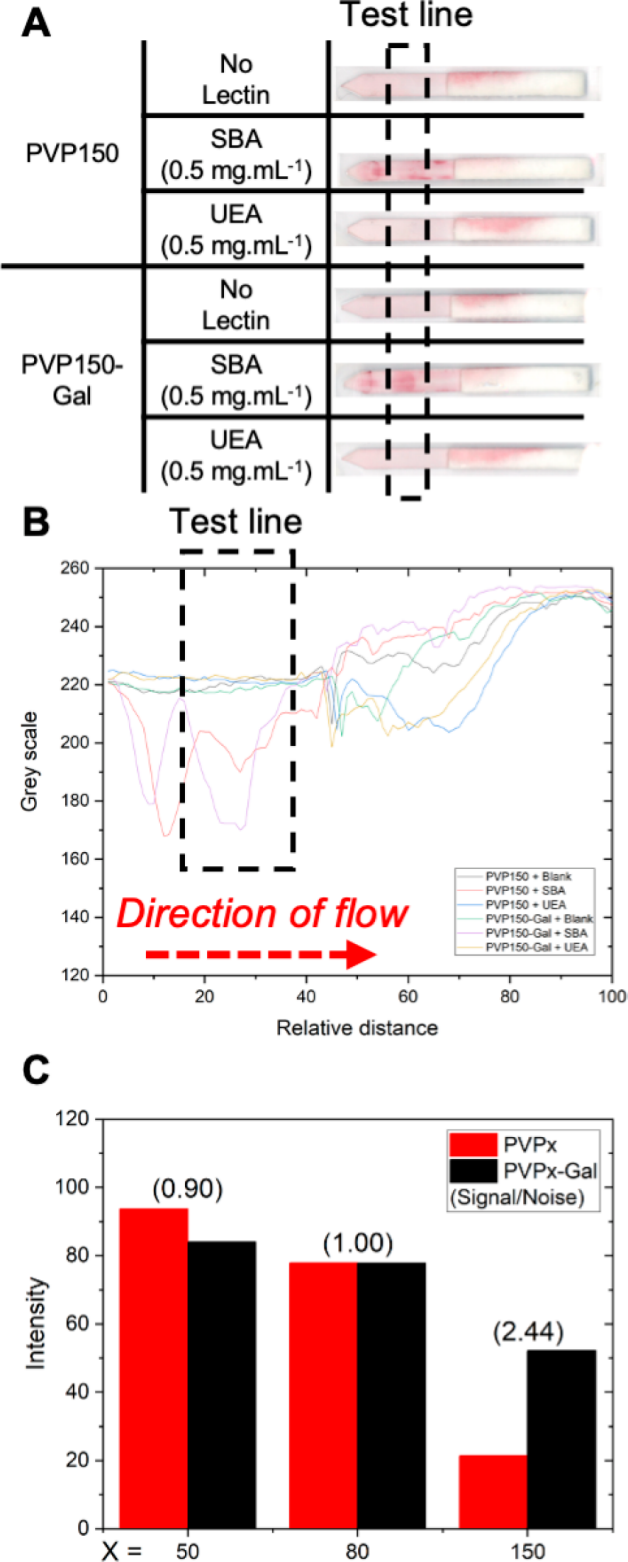
Lateral flow strips and analysis using test lines of PVP_150_–Gal and PVP_150_ (20 mg·mL^–1^). (A) Example lateral flow strips using test lines of PVP_150_–Gal and PVP_150_ (20 mg·mL^–1^) versus no analyte, SBA (0.5 mg·mL^–1^), and UEA (0.5 mg·mL^–1^), using Gal–PHEA_72_@AuNP_16_; (B) analysis of scanned lateral flow strips using test lines of PVP_150_–Gal and PVP_150_ (20 mg·mL^–1^) versus either no analyte, SBA (0.5 mg·mL^–1^), and UEA (0.5 mg·mL^–1^); (C) intensity of PVP_*x*_ (20 mg·mL^–1^) and PVP_*x*_–Gal (20 mg·mL^–1^) versus SBA (0.5 mg·mL^–1^) (signal-to-noise ratio (PVP_*x*_–Gal intensity/PVP_*x*_ intensity) is provided in brackets).

While biotin–streptavidin is an excellent model system, its low *K*_d_ (∼10^–14^ mol·dm^–3^)^40^ is not representative of many analyte-capture agent scenarios that have lower affinity (higher *K*_d_). Therefore, soybean agglutinin (SBA), a lectin with a known affinity for galactosamine, was chosen as an analyte. We have previously designed and validated an appropriate gold nanoparticle system (Gal–PHEA_72_@AuNP_16_) to sense specifically for SBA in an LFD device using protein agents to immobilize the glycan to the stationary phase.^13^ It was anticipated that the PVP test lines functionalized with galactosamine may not perform as well as their glycan–BSA counterpart (Galα1–3Galβ1–4GlcNAc–BSA). This is likely due to the loss of the cluster glycoside effect (the glycan–BSA used carried >20 glycans per BSA protein as reported by the manufacturer) and the use of galactosamine (with free anomeric position) as the binding glycan in the PVP system. Initial attempts, in triplicate, to use 20 mg·mL^–1^ galactosamine-functionalized PVPs and an SBA concentration in solution of 0.05 mg·mL^–1^ proved unsuccessful with no binding observed to the SBA (Supporting Information Table S16). However, no off-target binding was observed to either the no-lectin, UEA, or unfunctionalized PVP systems (in any test), which was promising. A higher concentration of SBA (0.5 mg·mL^–1^) was therefore chosen for the lateral flow glycoassay (Supporting Information Table S17 and Figure S32). While this concentration of SBA did lead to nonspecific binding of the SBA–particle complex to the unfunctionalized PVP test line in all cases and in all triplicates; stronger signals were observed in the PVP_150_–Gal system (Figure 8), with the PVP_150_–Gal system (signal) versus the unfunctionalized PVP_150_ system (noise) having a signal-to-noise ratio of 2.44 (Figure 8C). This indicates that the limit of detection (LOD) of SBA is between ∼0.5–0.05 mg·mL^–1^. This compares well to a system using the same nanoparticles in a setup against a test line of Galα1–3Galβ1–4GlcNAc–BSA (1 mg·mL^–1^), with an LOD of ∼0.02 mg·mL^–1^.^100^ Considering the PVP does not (likely) benefit from the cluster glycoside effect to the same extent as a multivalent protein surface,^41^ the LOD achieved is promising. Although, it is possible that the lectins can bind multiple PVP chains, depending on their exact orientation on the surface. Notably, the PVP-based system is not as sensitive as antibody-based LFDs, such as those for ricin (LOD ≈ 20 ng·mL^–1^)^42^ or a concanavalin A (LOD ∼ 0.1 μg·mL^–1^),^16^ but it does indicate the potential for the integration of polymer systems into LFDs.

Decreasing the concentration of the PVP test-line systems was attempted but yielded mixed results (Supporting Information Tables S18 and 19 and Figures S33 and 34), indicating that the 20 mg·mL^–1^ PVP_150_–Gal system is the optimum for this particular particle system and analyte. Interestingly, this is different from the concentration used in the biotin-functionalized PVP lateral flow system and the optimum chain length in some of the flow-through assays. This indicates the need to tune each system depending on the application, again highlighting the tunability benefits of polymer chemistry over protein-based systems. Furthermore, the background could be improved by adjusting the buffer, tuning the AuNP system, or treating the membrane. Meanwhile, the signal could be improved by printing the test line, rather than using “by hand” deposition of a test spot or using a more complex glycan with greater affinity for SBA. These sorts of modifications were however beyond the scope of this work that focuses on a proof of concept for polymeric test lines.

## Conclusions

Here, the concept of a fully synthetic, protein-free, polymeric lateral flow test-line is validated and explored for the first time. It is shown to be a promising alternative to the established protein-based anchoring reagents. Poly(vinylpyrrolidone), PVP, was identified as promising immobilization agent, based on its widespread use as a “blocking agent”, which is sufficiently hydrophobic to adhere to nitrocellulose stationary phases but still being water-soluble, which is essential for production/printing of the test line. PVP was synthesized by RAFT/MADIX polymerization using an *N*-hydroxy-succinimide (NHS)-functionalized chain-transfer agent, which allowed subsequent installation of a glycan or biotin, as a capture ligand. The polymer anchor was shown to allow capture in flow-through and lateral flow systems, leading to specific binding with limited off-target (nonspecific) binding. A key observation was that the chain length of the PVP (as well as the concentration applied) was crucial to optimize the signal generation and specificity. For example, in the flow-through system when targeting streptavidin-functionalized particles in the mobile phase, the best PVP–biotin chain length varied with the concentration of the test line used. Meanwhile, in the lateral flow system when targeting streptavidin, a 1 mg·mL^–1^ test line of PVP_150_–biotin was best, and in the lateral flow glycoassay, when targeting SBA, a 20 mg·mL^–1^ PVP_150_–Gal test line was best.

We anticipate that the polymeric system discussed (PVP) could be used as a multifunctional scaffold or platform to present other capture agents such as short amino acid or nucleotide sequences and enable a wider range of end-group functionality beyond amide chemistry (i.e., click chemistry approaches). The ability to tune the molecular weight of a polymeric test line will allow further fine-tuning, in contrast to protein-based anchors. Furthermore, the addition of multivalency to the system could also be explored while maintaining synthetic control over the number of capture agents per polymer anchor unit. Plus, there exists many thousands of potential (co)polymer structures, which provide further opportunities to refine the polymer test-line approach. In summary, the PVP scaffolds presented and validated here provide the first examples of a tunable and multifunctional polymeric test-line capture system for lateral flow devices and further epitomize the potential of applying polymer chemistry to LFDs.

## Experimental Section

### Materials

All chemicals were used as supplied unless otherwise stated. *N-*Hydroxyethyl acrylamide (97%), 4,4′-azobis(4-cyanovaleric acid) (ACVA, 98%), 4-(dimethylamino)pyridine (DMAP, >98%), mesitylene (reagent grade), triethylamine (TEA, >99%), sodium citrate tribasic dihydrate (>99%), gold(III) chloride trihydrate (99.9%), potassium phosphate tri basic (≥98%, reagent grade), *N*,*N*′-diisopropylcarbodiimide (DIC, 99%), 1-vinyl-2-pyrrolidone (≥98.0% for synthesis), DMSO (ACS reagent, ≥99.9%), deuterated DMSO (DMSO-*d*_6_, ≥99%), deuterium oxide (D_2_O, 99.9%), deuterated chloroform (CDCl_3_, 99.8%), deuterated methanol (CD_3_OD, (≥99.8%), diethyl ether (≥99.8%, ACS reagent grade), methanol (≥99.8%, ACS reagent grade), toluene (≥99.7%,), di-*tert*-butyl dicarbonate (≥98.0%), Tween-20 (molecular biology grade), HEPES, PVP40 (poly(vinylpyrrolidone)_400_ (average *M*_w_ ≈ 40 000), carbon disulfide (≥99.8%), acetone (≥99%), 1-dodecanethiol (≥98%), biotin (≥99%, HPLC lyophilized powder), 40 nm gold nanoparticles (OD1 in citrate buffer), streptavidin–gold (40 nm) from *Streptomyces avidinii*, pentafluorophenol (≥99%, reagent plus), *N*-hydroxysuccinimide (98%), ethylenediamine (≥99.5%), ethyl acetate (≥99.5%), trifluoroacetic acid (TFA, ≥99%, reagent plus), sodium azide (≥99.5%, reagent plus), and potassium permanganate (≥99%) were purchased from Sigma-Aldrich. Potassium ethyl xanthate (98%) was purchased from Alfa Aesar. DMF (>99%) and 2-bromo-2-methyl-propionic acid (98%) were purchased from Acros Organics. Galactosamine HCl and 1-ethyl-3-(3-(dimethylamino)propyl)carbodiimide hydrochloride (EDCI, >98%) were purchased from Carbosynth. Hexane fraction from petrol (lab reagent grade), DCM (99% lab reagent grade), sodium hydrogen carbonate (≥99%), ethyl acetate (≥99.7%, analytical reagent grade), sodium chloride (≥99.5%), calcium chloride, 40–60 petroleum ether (lab reagent grade), hydrochloric acid (∼37%, analytical grade), glacial acetic acid (analytical grade) and magnesium sulfate (reagent grade), THF (HPLC), chloroform (≥99%), Molecular Sieve type 4 Å nominal pore size (general purpose grade), and 1,4-dioxane (≥99%) were purchased from Thermo Fisher Scientific. Ethanol absolute was purchased from VWR International. Nitrocellulose Immunopore RP 90–150 s/4 cm 25 mm was purchased from GE Healthcare. Lateral flow backing cards, 60 by 301.58 mm (KN-PS1060.45 with KN211 adhesive), were purchased from Kenosha Tapes. Cellulose fiber wick material, 20 cm by 30 cm by 0.825 mm (290 gsm and 180 mL/min) (Surewick CFSP223000) was purchased from EMD Millipore. Soybean agglutinin and *Ulex Europaeus* Agglutinin I were purchased from Vector Laboratories. Spectra/Por 7 Dialysis Membrane Pretreated RC (regenerated cellulose) Tubing MWCO: 1 kDa was purchased from Spectrum Laboratories. Streptavidin lyophilized was purchased from Stratech Scientific. Ultrapure water used for buffers was Milli-Q grade, 18.2 mΩ·cm resistance.

### Synthetic Methods

#### MADIX Agent Synthesis: 2-(Ethoxycarbonothioylthio)-2-methylpropanoic Acid *N*-Hydroxysuccinimide Ester (MADIX1)

10.27g (61.50 mmol) of 2-bromo-2-methyl-propionic acid was dissolved in 60 mL of ethanol. 15.00 g (93.57 mmol) of potassium *O*-ethyl xanthate was added, and the mixture was stirred for 38 h at RTP. The reaction mixture was filtered under gravity, and the filtrate was diluted with 400 mL of diethyl ether. The organic layer was washed with water (200 mL × 3), and the aqueous layers were combined and acidified with 6 M HCl. The aqueous layers were extracted with diethyl ether (200 mL × 3) and combined with all organic layers. The solution was dried with MgSO_4_ and filtered under gravity. The solvent was removed under vacuum to form a yellow oil.

8.83 g (42.45 mmol) of crude product (2-((ethoxycarbonothioyl)thio)-2-methylpropanoic acid) and 9.50 g (82.54 mmol) of *N*-hydroxysuccinimide were added to an empty RBF and purged with nitrogen before 40 mL of anhydrous THF was added; the solution was then degassed for a further 20 min. The solution was cooled to 0 °C, and 8 mL (9.93 g, 78.65 mmol) of *N*,*N*-diisopropyl carbodiimide was added dropwise over 10 min. The flask was put under positive nitrogen pressure and stirred for 48 h. The solution was filtered under gravity, and the filtrate solvent was removed under vacuum. The crude solid was dissolved in 100 mL of diethyl ether and 100 mL of saturated NaHCO_3_ solution. The organic layer was washed with water (100 mL × 3) and 100 mL of brine once. The organic layer was dried with MgSO_4_ and filtered under gravity. The solvent was then removed from the filtrate under vacuum. The crude product was recrystallized in ethyl acetate overnight at −8 °C, washed with cold hexane, and dried to give yellow crystals (25.2%). δ_H_ (300 MHz, CDCl_3_) 4.69 (2H, q, *J* 7.0, OCH_2_), 2.85–2.81 (4H, m, C(O)CH_2_CH_2_C(O)), 1.76 (6H, s, C(CH_3_)_2_), 1.37 (3H, t, *J* 7.0, CH_2_C*H*_3_). δ_C_ (300 MHz, CDCl_3_) 208.92 (1C, SC(S)S), 171.43 (1C, OC(O)), 168.82 (2C, NC(O), 71.00 (1C, OCH_2_), 52.41 (1C, *C*(CH_3_)_2_), 26.15 (2C, C(O)*C*H_2_*C*H_2_C(O)), 25.73 (2C, C(*C*H_3_)_2_), 13.07 (1C, CH_2_*C*H_3_). *m*/*z* calculated as 305.36; found for ESI [M + Na]^+^ 328.1. FTIR (cm^–1^) 2989.32 and 2940.46 (methyl or methylene), 1779.80 (ester carbonyl), 1731.34 (amide), 1462 (methyl), 1202.06 (C=S), 1038.06 (S–C(S)–O).

#### Representative Polymerization of *N*-Vinylpyrrolidone (PVP80)

5.65 mL (5.43 g, 48.88 mmol) of *N*-vinylpyrrolidone, 0.010 g (0.036 mmol) of ACVA, and 0.0523 g (0.171 mmol) of MADIX1 were added to 8.5 mL of dioxane and degassed with nitrogen for 20 min. The reaction was stirred at 80 °C for 3 days. The solvent was removed under vacuum, and the solid was dialyzed using 0.5–1 kDa cellulose ester tubing in water. The dialyzed product was freeze-dried overnight to give a white powder. δ_H_ (300 MHz, CDCl_3_) 4.06–3.48 (80H, m, NCH_2_), 3.47–2.98 (184H, m, NC(O)CH_2_) 2.85–2.77 (4H, m, C(O)CH_2_CH_2_C(O)), 2.58–2.13 (253H, m, NC(O)CH_2_), 2.13–1.84 (206H, m, NCH_2_C*H*_*2*_), 1.84–1.03 (204H, m, (CH_3_)_2_ & NCHC*H*_*2*_ & OCH_2_C*H*_*3*_). FTIR (cm^–1^) 2926 (alkyl stretch), 1655 (lactam amide), 1422 (CH_2_).

PVP50 δ_H_ (300 MHz, CDCl_3_) 4.16–3.45 (50H, m, NCH_2_), 3.51–2.96 (100H, m, NC(O)CH_2_) 2.86–2.74 (4H, m, C(O)CH_2_CH_2_C(O)), 2.71–2.14 (129H, m, NC(O)CH_2_), 2.14–1.85 (111H, m, NCH_2_C*H*_*2*_), 1.85–1.01 (159H, m, (CH_3_)_2_ & NCHC*H*_*2*_ & OCH_2_C*H*_*3*_).

PVP150 δ_H_ (300 MHz, CDCl_3_) 4.11–3.46 (150H, m, NCH_2_), 3.46–2.92 (305H, m, NC(O)CH_2_) 2.85–2.75 (4H, m, C(O)CH_2_CH_2_C(O)), 2.69–2.12 (428H, m, NC(O)CH_2_), 2.12–1.84 (320H, m, NCH_2_C*H*_*2*_), 1.84–1.17 (306H, m, (CH_3_)_2_ & NCHC*H*_*2*_ & OCH_2_C*H*_*3*_).

#### Representative Poly(*N*-vinylpyrrolidone) (PVP80) Glycan Functionalization

26.6 mg (2.8 μmol) of polymer and 21.2 mg (0.099 mmol) of galactosamine HCl were dissolved in the minimum amount of DMSO and 37.5 μL of TEA, stirred for 3 days at RTP, and dialyzed using 0.5–1 kDa regenerated cellulose membrane tubing in water. The dialyzed product was freeze-dried overnight to give a pale-yellow powder (23.5 mg).

δ_H_ (300 MHz, CDCl_3_) 5.35–4.75 (anomeric 1H, m, C(O)OH), 4.04–3.51 (84H, m, CHN & glycan protons), 3.38–2.96 (184H, m, NCH_2_ & glycan protons), 2.51–2.11 (176H, m, NC(O)CH_2_ & glycan protons), 2.11–1.84 (172H, m, NCH_2_C*H*_*2*_), 1.84–1.01 (215H, m, (CH_3_)_2_ & NCHC*H*_*2*_ & OCH_2_C*H*_*3*_). FTIR (cm^–1^) 2920, 2877 (alkyl stretch) 1655 (lactam amide), 1422 (CH_2_)

#### Representative Poly(*N*-vinylpyrrolidone) (PVP80) Biotin Functionalization

A 6.5 mg (0.7 μmol) portion of polymer, 5 mg (17.46 μmol) of amino-functionalised biotin, and 27.5 μL of TEA were dissolved in the minimum volume of DMSO and stirred at RTP for 72 h. The reaction mixture was dialyzed using 1 kDa regenerated cellulose membrane in water and freeze-dried to give a white solid (5.6 mg). δ_H_ (300 MHz, CDCl_3_) 4.08–3.52 (82H, m, CHN & C(O)NHC*H*_2_), 3.42–2.97 (167H, NCH_2_, CHC*H*S, C*H*_2_NH_2_, CHCH*H*S, CHC*H*HS), 2.55–2.12 (226H, NC(O)CH_2_ & C*H*_2_C(O)NH), 2.12–1.85 (180H, NCH_2_C*H*_*2*_), 1.85–1.07 (193H, m, (CH_3_)_2_, NCHC*H*_*2*_, OCH_2_C*H*_*3*_, SCHC*H*_2_CH_2_C*H*_2_ & SCHCH_2_C*H*_2_CH_2_). FTIR (cm^–1^) 1634 (lactam amide)

### Lateral Flow Strip Running Protocol and Analysis

*A more detailed summary of dipstick manufacture, running, and analysis can be found in the*Supporting Information, *summarized here*. Test lines were added and dried onto the dipsticks; in flow-through, the analyte was deposited in place of a test line. 50 μL of running buffer (either with or without analyte) was agitated on a roller for 5 min. 45 μL of running buffer was added to a PCR tube, and a dipstick was added to the tube, so the dipstick protrudes from the top and the immobile phase (1 cm from nonwick end) is not below the solvent line. There was one test per tube, and each test was run for 20 min before drying at room temperature for 5 min. All tests were run in triplicate. All strips were scanned and exported to pdf before conversion to a jpeg file. The jpeg files were analyzed using ImageJ 1.51^43^ using the plot profile function to create a data set exported to Microsoft Excel for Mac. The data was exported to Origin 2019 64Bit, aligned, and averaged (mean). The data was then reduced by number of groups to 100 data points (nitrocellulose and wick) and plotted as gray value (scale) vs relative distance along the 100 data points.

## Supporting Information Available

Details of materials and characterization techniques used; polymer, glycan, and gold nanoparticle synthesis and characterization (DLS, UV–vis, NMR, SEC, FTIR, mass spectroscopy, TEM, and XPS); details of lateral flow dipstick and cassette construction, running and analysis methods used; raw scans of the lateral flow devices, analyzed data and signal-to-noise calculations (PDF)

## Notes

Data Access Statement: The research data supporting this publication can be found at http://wrap.warwick.ac.uk, and all images of test strips are in the Supporting Information.
