# Supplementary material for: End-Functionalized Poly(vinylpyrrolidone) for Ligand Display in Lateral Flow Device Test Lines
Source: ACS Polym Au. 2021 Nov 12;2(2):69–79. doi: 10.1021/acspolymersau.1c00032 (PMC7612620; doi:10.1021/acspolymersau.1c00032)
Supplement: Supplementary file 1 — lg1c00032_si_001.pdf [file lg1c00032_si_001.pdf]

Supporting Information

End-Functionalized Poly(Vinyl Pyrrolidone) for Ligand Display in Lateral Flow Device Test Lines

*Alexander N. Baker,[a] Thomas R. Congdon,[a,b] Sarah-Jane Richards,[a] Panagiotis G. Georgiou,[a] Marc Walker,[c] Simone Dedola,[d] Robert A. Field [d,e] and Matthew I. Gibson[a,b]**

[a] Department of Chemistry, University of Warwick, Gibbet Hill Road, CV4 7AL, Coventry, UK

[b] Warwick Medical School, University of Warwick, Gibbet Hill Road, CV4 7AL, Coventry, UK

[c] Department of Physics, University of Warwick, Gibbet Hill Road, CV4 7AL, Coventry, UK

[d] Iceni Diagnostics Ltd, Norwich Research Park, Norwich, NR4 7GJ, UK

[e] Department of Chemistry and Manchester Institute of Biotechnology, University of Manchester, Manchester, M1 7DN, UK

*<u>*Corresponding Author*:</u> m.i.gibson@warwick.ac.uk

Contents

Contents 2

Physical and Analytical Methods 5

NMR Spectroscopy 5

Mass Spectrometry 5

FT-IR Spectroscopy 5

Size Exclusion Chromatography 5

X-ray Photoelectron Spectroscopy (XPS) 6

Dynamic Light Scattering 6

UV-vis Spectroscopy 7

Transmission Electron Microscopy 7

Image Collection of Lateral Flow Dipsticks and Devices 7

Solvent drying 7

Materials 8

Synthetic Methods 10

Synthesis of 2-(dodecylthiocarbanothionylthio)-2-methyl propionic acid (DMP) 10

Synthesis of pentafluorophenyl-2-dodecylthiocarbonothioylthio)-2-methylpropanoate (PFP-DMP) 11

Representative Polymerization of 2-hydroxyethyl acrylamide (PHEA72) 12

Representative Poly(N-hydroxyethyl acrylamide) (PHEA72) Glycan Functionalization 13

Representative Poly(N-hydroxyethyl acrylamide) (PHEA72) Biotin Functionalization 14

MADIX Agent Synthesis. 2-(ethoxycarbonothioylthio)-2-methylpropanoic acid N-hydroxsuccinimide ester (MADIX1) 15

Representative Polymerization of N-vinyl pyrrolidone (PVP80) 17

Representative Poly(N-vinyl pyrrolidone) (PVP80) Sugar Functionalization 18

Representative Poly(N-vinyl pyrrolidone) (PVP80) Biotin Functionalization 18

Synthesis of diamine t-BOC[3] 19

Synthesis of biotin diamine t-BOC[3] 20

Synthesis of biotin-NH_2[4] 21

Citrate-Stabilized 16 nm Gold Nanoparticle Synthesis[5] 22

Gold Nanoparticle Polymer Coating Functionalization – 16 nm 22

Gold Nanoparticle Polymer Coating Functionalization – 40 nm 22

Lateral Flow Strip Production, Running and Analysis Protocols 23

Protocol for Manufacturing Lateral Flow Strips 23

Protocol for Test Line Addition to the Lateral Flow Strips 23

Protocol for Running Lateral Flow Test Without Target Analyte in Buffer 24

Protocol for Running Lateral Flow Test with Target Analyte in Buffer 24

Standard Protocol for Lateral Flow Strip Analysis 25

Lateral Flow Assay Buffer - 10× HEPES buffer (10% PVP_{400}) in 100 mL H_2O 26

Intensity Calculations 26

Signal-to-Noise Calculations 26

Additional Data and Figures 27

AuNP Data 33

DLS and UV-vis Data....33

Flow-Through Dipsticks and Analysis – Biotin and Streptavidin....44

Lateral Flow Dipsticks and Analysis – Biotin and Streptavidin....50

Lateral Flow Dipsticks and Analysis – Galactosamine and SBA....65

XPS Data....72

References....83

Physical and Analytical Methods

NMR Spectroscopy

^{1}H-NMR, ^{13}C-NMR and ^{19}F-NMR spectra were recorded at 300 MHz or 400 MHz on a Bruker DPX-300 or DPX-400 spectrometer respectively, with chloroform-*d* ($CDCl_3$), deuterated DMSO (DMSO-d_6), deuterated methanol (MeOD) or deuterium oxide (D_2O) as the solvent. Chemical shifts of protons are reported as δ in parts per million (ppm) and are relative to either $CDCl_3$ (7.26), DMSO-d_6 (2.50), MeOD (4.87, 3.31) or D_2O (4.79).

Mass Spectrometry

Low resolution mass spectra (LRMS) were recorded on a Bruker Esquire 2000 spectrometer using electrospray ionisation (ESI). M/z values are reported in Daltons.

FT-IR Spectroscopy

Fourier Transform-Infrared (FT-IR) spectroscopy measurements were carried out using an Agilent Cary 630 FT-IR spectrometer, in the range of 650 to 4000 cm^{-1}.

Size Exclusion Chromatography

Size exclusion chromatography (SEC) analysis was performed on an Agilent Infinity II MDS instrument equipped with differential refractive index (DRI), viscometry (VS), dual angle light scatter (LS) and variable wavelength UV detectors. The system was equipped with 2 x PLgel Mixed D columns (300 x 7.5 mm) and a PLgel 5 µm guard column. The mobile phase used was DMF (HPLC grade) containing 5 mM NH_4BF_4 at 50 °C at flow rate of 1.0 $mL.min^{-1}$. Poly(methyl methacrylate) (PMMA) standards (Agilent EasyVials) were used for calibration between 955,000 – 550 $g.mol^{-1}$. Analyte samples were filtered through a nylon membrane with 0.22 µm pore size before injection. Number average molecular weights (M_n), weight average

molecular weights (M_w) and dispersities ($Đ_M = M_w/M_n$) were determined by conventional calibration using Agilent GPC/SEC software.

X-ray Photoelectron Spectroscopy (XPS)

The samples were attached to electrically-conductive carbon tape, mounted on to a sample bar and loaded into a Kratos Axis Ultra DLD spectrometer which possesses a base pressure below 1×10^{-10} mbar. XPS measurements were performed in the main analysis chamber, with the sample being illuminated using a monochromated Al Kα x-ray source. The measurements were conducted at room temperature and at a take-off angle of 90° with respect to the surface parallel. The core level spectra were recorded using a pass energy of 20 eV (resolution approx. 0.4 eV), from an analysis area of 300 μm x 700 μm. The spectrometer work function and binding energy scale of the spectrometer were calibrated using the Fermi edge and $3d_{5/2}$ peak recorded from a polycrystalline Ag sample prior to the commencement of the experiments. In order to prevent surface charging the surface was flooded with a beam of low energy electrons throughout the experiment and this necessitated recalibration of the binding energy scale. To achieve this, the C-C/C-H component of the C 1*s* spectrum was referenced to 285.0 eV. The data was analyzed in the CasaXPS package, using Shirley backgrounds and mixed Gaussian-Lorentzian (Voigt) lineshapes. For compositional analysis, the analyser transmission function has been determined using clean metallic foils to determine the detection efficiency across the full binding energy range.

Dynamic Light Scattering

Hydrodynamic diameters (D_h) and size distributions of particles were determined by dynamic light scattering (DLS) using a Malvern Zetasizer Nano ZS with a 4 mW He-Ne 633 nm laser module operating at 25 °C. Measurements were carried out at an angle of 173° (back scattering), and results were analyzed using Malvern DTS 7.03 software. All determinations

were repeated 5 times with at least 10 measurements recorded for each run. D_h values were calculated using the Stokes-Einstein equation where particles are assumed to be spherical.

UV-vis Spectroscopy

Absorbance measurements were recorded on an Agilent Cary 60 UV-Vis Spectrophotometer and on a BioTek Epoch microplate reader.

Transmission Electron Microscopy

Dry-state stained TEM imaging was performed on a JEOL JEM-2100Plus microscope operating at an acceleration voltage of 200 kV. All dry-state samples were diluted with deionized water and then deposited onto formvar-coated copper grids.

Image Collection of Lateral Flow Dipsticks and Devices

All devices were scanned using a Kyocera TASKalfa 5550ci printer to a pdf file that was converted to a jpeg. The jpeg was analyzed in ImageJ 1.51.[1] None of the images in this ESI have been image adjusted i.e. no changes/enhancements have been made from the original scan images. The main paper images may have been enhanced cropped to improve clarity.

Solvent drying

4Å molecular sieves were activated either by heat or using microwave energy (600W). A 20% w/v. of sieves:solvent was used, the solvent was degassed with nitrogen for 30 minutes with the sieves present and then left overnight before the solvent was used.

Materials

All chemicals were used as supplied unless otherwise stated. *N*-Hydroxyethyl acrylamide (97%), 4,4'-azobis(4-cyanovaleric acid) (ACVA, 98%), 4-dimethylaminopyridine (DMAP, > 98%), mesitylene (reagent grade), triethylamine (TEA, > 99%), sodium citrate tribasic dihydrate (> 99 %), gold(III) chloride trihydrate (99.9%), potassium phosphate tri basic (≥ 98%, reagent grade), *N*,*N*'-diisopropylcarbodiimide (DIC, 99%), 1-vinyl-2-pyrrolidone (≥ 98.0% for synthesis), DMSO (ACS reagent, (≥ 99.9%), deuterated DMSO (DMSO-d_6, ≥ 99%), deuterium oxide (D_2O, 99.9%), deuterated chloroform ($CDCl_3$, 99.8%), deuterated methanol (CD_3OD, (≥ 99.8%), diethyl ether (≥ 99.8%, ACS reagent grade), methanol (≥ 99.8%, ACS reagent grade), toluene (≥ 99.7%,), di-*tert*-butyl dicarbonate (≥ 98.0%), Tween-20 (molecular biology grade), HEPES, PVP40 (poly(vinyl pyrrolidone)$_{400}$ (Average Mw ~40,000)), carbon disulphide (≥ 99.8%), acetone (≥ 99%), 1-dodecane thiol (≥ 98%), biotin (≥ 99%, HPLC lyophilized powder), 40nm gold nanoparticles (OD1 in citrate buffer), streptavidin-gold (40nm) from *Streptomyces avidinii*, pentafluorophenol (≥ 99%, reagent plus), *N*-hydroxysuccinimide (98%), ethylenediamine (≥ 99.5%), ethyl acetate (≥ 99.5%), trifluoroacetic acid (TFA, ≥ 99%, reagent plus), sodium azide (, ≥ 99.5%, reagent plus) and potassium permanganate (≥ 99%,) were purchased from Sigma-Aldrich. Potassium ethyl xanthate (98%) was purchased from Alfa Aesar. DMF (> 99%), 2-bromo-2-methyl-propionic acid (98%) were purchased from Acros Organics. Galactosamine HCl and 1-Ethyl-3-(3-dimethylaminopropyl)carbodiimide hydrochloride (EDCI, > 98%),was purchased from Carbosynth. Hexane fraction from petrol (lab reagent grade), DCM (99% lab reagent grade), sodium hydrogen carbonate (≥ 99%), ethyl acetate (≥ 99.7%, analytical reagent grade), sodium chloride (≥ 99.5%), calcium chloride, 40-60 petroleum ether (lab reagent grade), hydrochloric acid (~37%, analytical grade), glacial acetic acid (analytical grade) and magnesium sulphate (reagent grade), THF (HPLC), chloroform (≥ 99%), Molecular Sieve type 4Å nominal pore

size (general purpose grade) and 1,4-dioxane (≥ 99%) were purchased from Thermo Fisher Scientific. Ethanol absolute was purchased from VWR International.

Nitrocellulose Immunopore RP 90-150 s/4cm 25mm was purchased from GE Healthcare. Lateral flow backing cards 60mm by 301.58mm (KN-PS1060.45 with KN211 adhesive) was purchased from Kenosha Tapes. Cellulose fibre wick material 20 cm by 30 cm by 0.825 mm (290 gsm and 180 mL/min) (Surewick CFSP223000) was purchased from EMD Millipore.

Soybean agglutinin and *Ulex Europaeus* Agglutinin I were purchased from Vector Laboratories.

Spectra/Por 7 Dialysis Membrane Pre-treated RC (regenerated cellulose) Tubing MWCO: 1 kD was purchased from Spectrum Laboratories.

Streptavidin lyophilized was purchased from Stratech Scientific.

Ultra-pure water used for buffers was MilliQ grade 18.2 mΩ resistance.

Synthetic Methods

Synthesis of 2-(dodecylthiocarbanothionylthio)-2-methyl propionic acid (DMP)

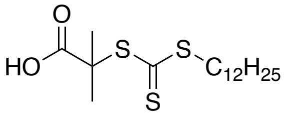

This was synthesized, according to a previously published procedure.[2] 2.00 g (9.88 mmol) of 1-dodecane thiol was added dropwise to stirring 2.10 g (9.89 mmol) of K_3PO_4 in 30mL of acetone at RTP, the mixture was left to stir for 25 minutes to form a white suspension. 2.05 g (26.93 mmol) of carbon disulphide was then added and left for 10 minutes, a yellow solution formed. 1.5 g (8.98 mmol) of 2-bromo-2-methyl-propionic acid was then added and the solution left to stir for 16 hours. The solvent was removed under vacuum. The crude product was dissolved in 100 mL of 1M HCl and extracted with DCM (2×100 mL). The organic layer was washed with 200 mL water and 200 mL brine. The organic layer was dried with $MgSO_4$ and filtered under gravity. The solvent was then removed from the filtrate under vacuum. The crude product was purified using a silica column (40-60 PET:DCM:glacial acetic acid 75:24:1) and recrystalized in n-hexane to give a yellow solid (58%). δ_H (300 MHz, $CDCl_3$) 3.28 (2H, t, *J* 7.5, $SC\mathit{H}_2CH_2$), 1.80 - 1.45 (8H, m, $C(CH_3)_2$ and $SCH_2C\mathit{H}_2$), 1.45 - 1.2 (18H, m, $(C\mathit{H}_2)_9CH_3$), 0.87 (3H, t, *J* 6.0, CH_3). δ_C (400 MHz, $CDCl_3$) 221.0 (1C, SC(S)S), 178.3 (1C, C(O)), 55.7 (1C, $\mathit{C}(CH_3)_2$), 37.7 (1C, SCH_2), 32.1 - 28.0 (9C, $SCH_2(\mathit{C}H_2)_9$), 25.4 (2C, $C(\mathit{C}H_3)_2$), 22.8 (1C, $\mathit{C}H_2CH_3$), 14.3 (1C, $CH_2\mathit{C}H_3$). m/z calculated as 364.16; found for ESI $[M+H]^+$ 365.3 and $[M+Na]^+$ 387.3. FTIR (cm^{-1}) – 2956, 2916.6 & 2850 (methyl and methylene), 1702 (ester C=O), 1459, 1437 & 1413 (methyl and methylene), 1280 ($C(CH_3)_2$), 1064 (S-C(S)-S).

Synthesis of pentafluorophenyl-2-dodecylthiocarbonothioylthio)-2-methylpropanoate (PFP-DMP)

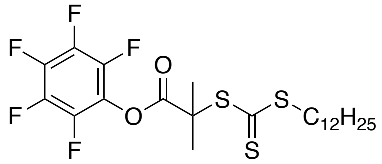

This was synthesized, according to a previously published procedure.[2] 4.06 g (11.13 mmol) of DMP, 3.65 g (19.04 mmol) of EDC and 2.30 g (18.82 mmol) of DMAP were dissolved in 160 mL of DCM and degassed for 30 minutes. 7.28 g (39.55 mmol) of pentaflurophenol was added in 20 mL of DCM and the mixture stirred for 18 hours at RTP. The organic layer was washed with 3 M HCl (200mL), 1 M $NaHCO_3$ (200 mL) and 0.5 M NaCl (200 mL). The organic layer was dried with $MgSO_4$ and filtered under gravity. The solvent was then removed from the filtrate under vacuum. The crude product was recrystallized in ethyl acetate (or hexane) overnight at -8°C and dried to give yellow crystals (90.9%). δ_H (300 MHz, $CDCl_3$) 3.31 (2H, t, *J* 7.5, $SC\mathit{H_2}CH_2$), 1.86 (6H, s, $C(CH_3)_2$), 1.69 (2H, qn, *J* 7.5, SCH_2), 1.48 - 1.16 (18H, m, $C\mathit{H_2}C\mathit{H_2}C\mathit{H_2}C\mathit{H_2}C\mathit{H_2}C\mathit{H_2}C\mathit{H_2}C\mathit{H_2}C\mathit{H_2}CH_3$), 0.94 - 0.82 (3H, m, CH_3). δ_C (300 MHz, $CDCl_3$) 220.1 (1C, SC(S)S), 169.7 (1C, C(O)), 143.1 (2C, meta C), 139.8 (1C, ipso C), 139.6 (1C, para C), 136.3 (2C, Ortho C), 55.5 (1C, $\mathit{C}(CH_3)_2$), 37.3 (1C, SCH_2), 32.0 - 22.8 (10C, $SCH_2(\mathit{C}H_2)_{10}$), 25.4 (2C, $C(\mathit{C}H_3)_2$), 14.1 (1C, $CH_2\mathit{C}H_3$). δ_F (300 MHz, $CDCl_3$) -151.44 - -151.61 (2F, m, $OCC_2\mathit{H_2}C_2H_2CH$), -148.50 (1F, t, *J* 21.5, $OCC_2H_2C_2H_2C\mathit{H}$), -162.23 - -162.47 (2F, m, $OCC_2H_2C_2\mathit{H_2}CH$). m/z calculated as 530.14; found for ESI $[M+Na]^+$ 553.3 and $[M+CH_3CN+Na]^+$ 593.5. FTIR (cm^{-1}) – 2956, 2917 & 2850 (methyl and methylene), 1702 (ester C=O), 1519 (aromatic C=C or C-F), 1460, 1437 & 1413 (methyl and methylene), 1280 ($C(CH_3)_2$), 1068 (S-C(S)-S).

Representative Polymerization of 2-hydroxyethyl acrylamide (PHEA72)

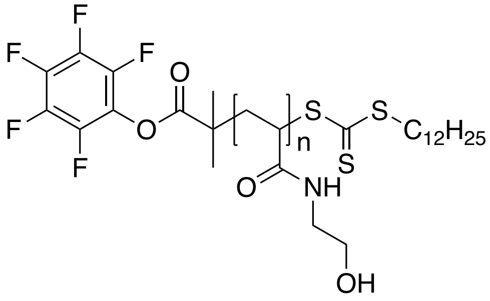

PHEA72 as representative example. 4.38 g (38 mmol) of 2-hydroxyethyl acrylamide, 0.0595 g (0.21 mmol) of ACVA and 0.5008 g (0.91 mmol) of PFP-DMP was added to 22 mL 1:1 toluene:methanol and degassed with nitrogen for 30 minutes. The reaction vessel was stirred and heated to 70 °C for 2 hours. The solvent was removed under vacuum. The crude product was dissolved in the minimum amount of methanol. Diethyl ether cooled in liquid nitrogen was added to the methanol to form a precipitate. The mixture was centrifuged for 2 minutes at 13 krpm and the liquid decanted off. The solid was dissolved in methanol and removed under vacuum to give a yellow crystalline solid. PHEA72 - δ_H (300 MHz, D_2O) 8.30 - 7.96 (34H, m, N*H*), 3.96 - 3.52 (126H, m, NHCH_2), 3.52 - 3.07 (155H, m, CH_2OH & SCH_2), 2.36 - 1.88 (70H, m, CH_2C*H*C(O) & C(CH_3)$_2$), 1.88 - 1.03 (148H, m, CH_2CHC(O) & CH_2CH_2CH_2CH_2CH_2CH_2CH_2CH_2C$H_2$$CH_2CH_3$), 0.82 - 0.70 (5H, m, C$H_2CH_3$). δ_F (300 MHz, D_2O) - 151.0 - -165.0 (5F, m, C_6F_5). FTIR (cm^{-1}) – 3267 (OH, broad), 3088 & 2924 (C(O)NH and NH), 1638 & 1545 (C(O)NH).

PHEA53 - δ_H (300 MHz, D_2O) 8.34 - 7.98 (4H, m, N*H*), 4.01 - 3.56 (90H, m, NHCH_2), 3.56 - 3.07 (91H, m, CH_2OH & SCH_2), 2.40 - 1.90 (47H, m, CH_2C*H*C(O) & C(CH_3)$_2$), 1.90 - 0.99 (123H, m, CH_2CHC(O) & CH_2CH_2CH_2CH_2CH_2CH_2CH_2CH_2C$H_2$$CH_2CH_3$), 0.82 - 0.72 (5H, m, C$H_2CH_3$)

PHEA110 - δ_H (300 MHz, D_2O) 8.24 - 8.02 (28H, m, N*H*), 3.83 - 3.51 (239H, m, NHCH_2), 3.51 - 3.08 (293H, m, CH_2OH & SCH_2), 2.40 - 1.90 (117H, m, CH_2C*H*C(O) & C(CH_3)$_2$), 1.90

- 1.03 (273H, m, C*H*$_2$CHC(O) & C*H*$_2$C*H*$_2$C*H*$_2$C*H*$_2$C*H*$_2$C*H*$_2$C*H*$_2$C*H*$_2$C*H*$_2$CH$_2$CH$_3$), 0.86 - 0.73 (5H, m, C*H*$_2$C*H*$_3$)

Representative Poly(N-hydroxyethyl acrylamide) (PHEA72) Glycan Functionalization

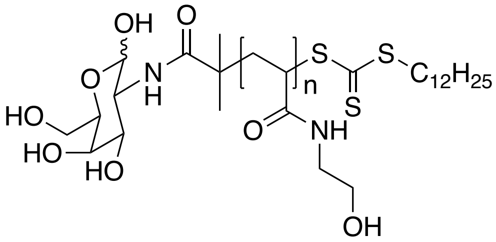

0.2516 g (0.028 mmol) of poly(2-hydroxyethyl acrylamide) and 0.093 g (0.43 mmol) of galactosamine HCl were added to 30 mL of DMF containing 0.05 M TEA. The reaction was stirred at 50°C for 16 hours. Solvent was removed under vacuum. The crude product was dissolved in the minimum amount of methanol at RTP before cooling in a liquid nitrogen bath. Diethyl ether cooled in liquid nitrogen was added to the methanol to form a precipitate. The mixture was centrifuged for 2 minutes at 13 krpm and the liquid decanted off. The solid was dissolved in methanol and removed under vacuum to give an orange/brown crystalline solid. δ_H (400MHz, D_2O) 8.33 - 8.01 (6H, m, NH), 4.95 - 4.89 (6H, anomeric protons), 3.99 - 3.54 (~145H, m, NHC*H*$_2$ & glycan protons), 3.54 - 3.19 (~233H, m, C*H*$_2$OH & SC*H*$_2$ & glycan protons + diethyl ether impurity), 2.37 - 1.87 (~60H, m, CH$_2$C*H*C(O), C(CH$_3$)$_2$ & glycan protons), 1.87 - 1.07 (152H, m, C*H*$_2$CHC(O) & C*H*$_2$C*H*$_2$C*H*$_2$C*H*$_2$C*H*$_2$C*H*$_2$C*H*$_2$C*H*$_2$C*H*$_2$CH$_3$ + diethyl ether impurity), 0.93 - 0.72 (5H, m, CH$_2$CH$_3$). FTIR (cm^{-1}) – 3274 (OH, broad), 3104 & 2929 (C(O)NH and NH), 1638 & 1552 (C(O)NH).

Representative Poly(N-hydroxyethyl acrylamide) (PHEA72) Biotin Functionalization

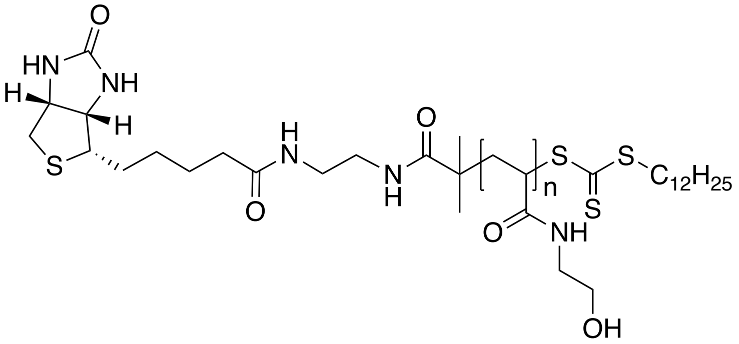

85.5 mg (0.0096 mmol) of PHEA72 and 19.45 mg (0.068 mmol) of biotin-NH_2 was dissolved in 10 mL of DMF containing 100 µL TEA. The reaction was stirred at 50°C for 16 hours. Solvent was removed under vacuum. The crude product was dissolved in the minimum amount of methanol at RTP before cooling in a liquid nitrogen bath. Diethyl ether cooled in liquid nitrogen was added to the methanol to form a precipitate. The mixture was centrifuged for 2 minutes at 13 krpm and the liquid decanted off. The solid was dissolved in methanol and removed under vacuum to give an orange/yellow crystalline solid. δ_H (400MHz, D_2O) 8.00 - 7.91 (4H, C(O)NH), 4.65 - 4.58 (1H, m, C*H*CH_2(S)), 4.47 - 4.39 (1H, m, C*H*CH(S)), 3.90 - 3.55 (85H, m, NHC*H₂*), 3.55 - 3.10 (141H, m, CH_2OH, C(O)NHCH_2CH_2NH, CHC*H*S, C(O)NHCH_2CH_2NH & CHCH*H*S), 2.82 - 2.68 (1H, m, CHC*H*HS), 2.45 - 1.87 (46H, m, CH_2C*H*C(O), $C(CH_3)_2$, CH_2C(O)NH), 1.87 - 1.07 (97H, m, CH_2CHC(O) & $CH_2CH_2CH_2CH_2CH_2CH_2CH_2CH_2CH_2CH_3$, SCH$CH_2CH_2CH_2$ & SCH$CH_2CH_2CH_2$), 0.86 - 0.77 (1H, m, CH_2CH_3). FTIR (cm^{-1}) – 3248 (OH Broad), 1636 (C(O)NH).

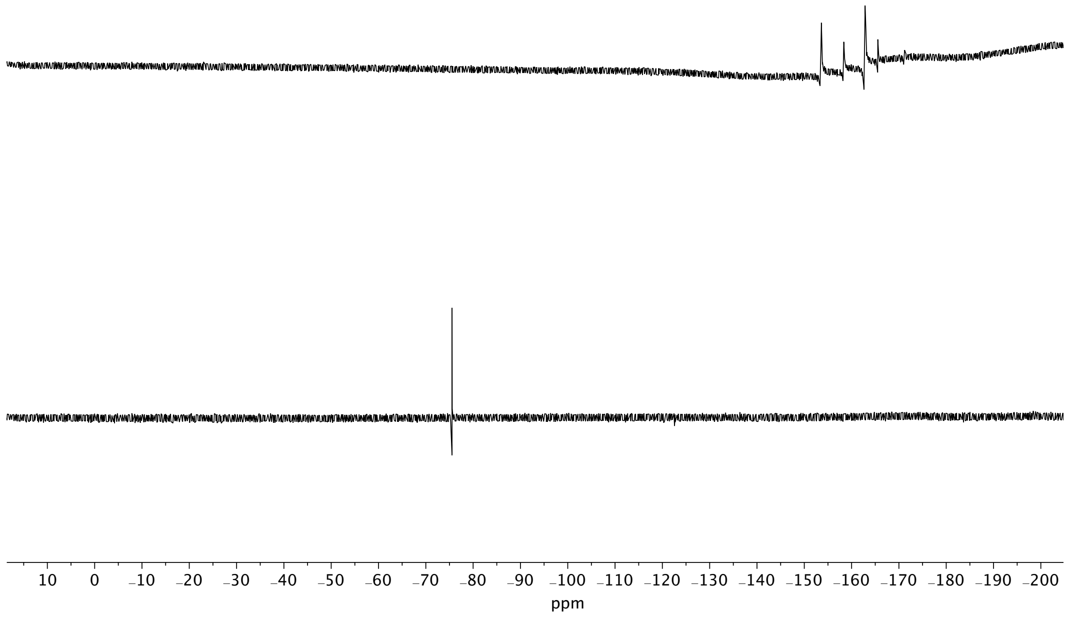

Figure S1. ^{19}F NMR of PHEA72 before (Top) and after (bottom) biotin functionalization

MADIX Agent Synthesis - 2-(ethoxycarbonothioylthio)-2-methylpropanoic acid N-hydroxsuccinimide ester (MADIX1)

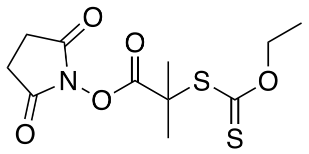

10.27g (61.50 mmol) of 2-bromo-2-methyl-propionic acid was dissolved in 60 mL of ethanol. 15.00 g (93.57 mmol) of potassium *O*-ethyl xanthate was added and the mixture stirred for 38 hours at RTP. The reaction mixture was filtered under gravity and the filtrate was diluted with 400 mL diethyl ether. The organic layer was washed with water (200 mL×3) and the aqueous layers combined and acidified with 6M HCl. The aqueous layers were extracted with diethyl ether (200 mL×3) and combined with all organic layers. The solution was dried with $MgSO_4$ and filtered under gravity. The solvent was removed under vacuum to form a yellow oil.

8.83 g (42.45 mmol) of crude product (2-((ethoxycarbonothioyl)thio)-2-methylpropanoic acid) and 9.50 g (82.54 mmol) of *N*-hydroxy succinimide was added to an empty RBF and purged

with nitrogen before 40mL of anhydrous THF was added, the solution was then degassed for a further 20 minutes. The solution was cooled to 0°C and 8 mL (9.93 g, 78.65 mmol) of *N*-*N*-diisopropyl carbodiimide was added dropwise over 10 minutes. The flask was put under positive nitrogen pressure and stirred for 48 hours. The solution was filtered under gravity and the filtrate solvent removed under vacuum. The crude solid was dissolved in 100 mL diethyl ether and 100 mL saturated $NaHCO_3$ solution. The organic layer was washed with water (100 mL ×3) and 100 mL brine once. The organic layer was dried with $MgSO_4$ and filtered under gravity. The solvent was then removed from the filtrate under vacuum. The crude product was recrystallized in ethyl acetate overnight at -8°C, washed with cold hexane and dried to give yellow crystals (25.2%). δ_H (300MHz, $CDCl_3$) 4.69 (2H, q, *J* 7.0, OCH_2), 2.85-2.81 (4H, m, $C(O)CH_2CH_2C(O)$), 1.76 (6H, s, $C(CH_3)_2$), 1.37 (3H, t, *J* 7.0, CH_2CH_3). δ_C (300MHz, $CDCl_3$) 208.92 (1C, SC(S)S), 171.43 (1C, OC(O)), 168.82 (2C, NC(O), 71.00 (1C, OCH_2), 52.41 (1C, $C(CH_3)_2$), 26.15 (2C, $C(O)CH_2CH_2C(O)$), 25.73 (2C, $C(CH_3)_2$), 13.07 (1C, CH_2CH_3). m/z calculated as 305.36; found for ESI $[M+Na]^+$ 328.1. FTIR (cm^{-1}) – 2989.32 & 2940.46 (methyl or methylene), 1779.80 (ester carbonyl), 1731.34 (amide), 1462 (methyl), 1202.06 (C=S), 1038.06 (S-C(S)-O).

Representative Polymerization of N-vinyl pyrrolidone (PVP80)

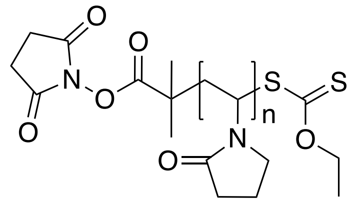

5.65 mL (5.43 g, 48.88 mmol) *N*-vinyl pyrrolidone, 0.010 g (0.036 mmol) ACVA and 0.0523 g (0.171 mmol) MADIX1 were added to 8.5 mL dioxane and degassed with nitrogen for 20 minutes. The reaction was stirred at 80℃ for 3 days. The solvent was removed under vacuum and the solid dialyzed using 0.5-1 kDa cellulose ester tubing in water. The dialyzed product was freeze dried overnight to give a white powder. δ_H (300 MHz, $CDCl_3$) 4.06 - 3.48 (80H, m, NCH_2), 3.47 - 2.98 (184H, m, $NC(O)CH_2$) 2.85 - 2.77 (4H, m, $C(O)CH_2CH_2C(O)$), 2.58 - 2.13 (253H, m, $NC(O)CH_2$), 2.13 - 1.84 (206H, m, NCH_2CH_2), 1.84 - 1.03 (204H, m, $(CH_3)_2$ & $NCHCH_2$ & OCH_2CH_3). FTIR (cm^{-1}) – 2926 (alkyl stretch) 1655 (lactam amide), 1422 (CH_2)

PVP50 - δ_H (300 MHz, $CDCl_3$) 4.16 - 3.45 (50H, m, NCH_2), 3.51 - 2.96 (100H, m, $NC(O)CH_2$) 2.86 - 2.74 (4H, m, $C(O)CH_2CH_2C(O)$), 2.71 - 2.14 (129H, m, $NC(O)CH_2$), 2.14 - 1.85 (111H, m, NCH_2CH_2), 1.85 - 1.01 (159H, m, $(CH_3)_2$ & $NCHCH_2$ & OCH_2CH_3)

PVP150 - δ_H (300 MHz, $CDCl_3$) 4.11 - 3.46 (150H, m, NCH_2), 3.46 - 2.92 (305H, m, $NC(O)CH_2$) 2.85 - 2.75 (4H, m, $C(O)CH_2CH_2C(O)$), 2.69 - 2.12 (428H, m, $NC(O)CH_2$), 2.12 - 1.84 (320H, m, NCH_2CH_2), 1.84 - 1.17 (306H, m, $(CH_3)_2$ & $NCHCH_2$ & OCH_2CH_3)

Representative Poly(N-vinyl pyrrolidone) (PVP80) Sugar Functionalization

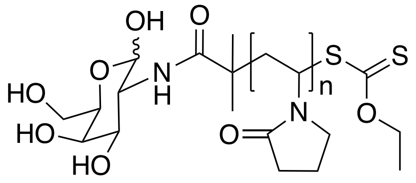

26.6 mg (2.8 μmol) of polymer and 21.2 mg (0.099 mmol) galactosamine HCl were dissolved in the minimum amount of DMSO and 37.5 μL TEA. Stirred for 3 days at RTP and dialyzed using 0.5-1 kDa regenerated cellulose membrane tubing in water. The dialyzed product was freeze dried overnight to give a pale-yellow powder (23.5 mg).

δ_H (300MHz, $CDCl_3$) 5.35 - 4.75 (anomeric 1H, m, C(O)OH), 4.04 - 3.51 (84H, m, CHN & glycan protons), 3.38 - 2.96 (184H, m, NCH_2 & glycan protons), 2.51 - 2.11 (176H, m, NC(O)CH_2 & glycan protons), 2.11 - 1.84 (172H, m, $NCH_2C\mathit{H_2}$), 1.84 - 1.01 (215H, m, $(CH_3)_2$ & $NCHC\mathit{H_2}$ & $OCH_2C\mathit{H_3}$). FTIR (cm^{-1}) – 2920, 2877 (alkyl stretch) 1655 (lactam amide), 1422 (CH_2)

Representative Poly(N-vinyl pyrrolidone) (PVP80) Biotin Functionalization

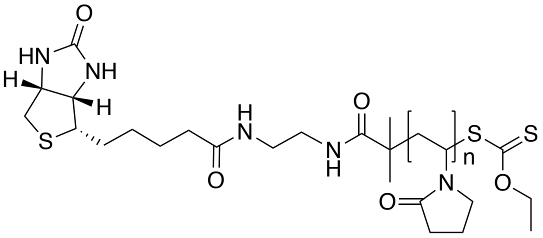

6.5 mg (0.7 μmol) of polymer, 5 mg (17.46 μmol) and 27.5 μL of TEA was dissolved in the minimum volume of DMSO and stirred at RTP for 72 hours. The reaction mixture was dialyzed using 1 kDa regenerated cellulose membrane in water and freeze dried to give a white solid (5.6 mg). δ_H (300MHz, $CDCl_3$) 4.08 - 3.52 (82H, m, CHN & C(O)NHC$\mathit{H_2}$), 3.42 - 2.97 (167H, NCH_2, CHC*H*S, C$\mathit{H_2}$$NH_2$, CHCH*H*S, CHC*H*HS), 2.55 - 2.12 (226H, NC(O)CH_2 &

$CH_2C(O)NH$), 2.12 - 1.85 (180H, NCH_2CH_2), 1.85 - 1.07 (193H, m, $(CH_3)_2$, $NCHCH_2$, OCH_2CH_3, $SCHCH_2CH_2CH_2$ & $SCHCH_2CH_2CH_2$). FTIR (cm^{-1}) – 1634 (lactam amide)

Synthesis of diamine t-BOC[3]

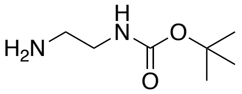

14 mL (200 mmol) of 1,2-diaminethane (ethylene diamine) was added to 200 mL of chloroform and cooled to 0°C. 4.44 g (20 mmol) of di-*tert*-butyl dicarbonate (BOC_2O) was added to 100 mL of chloroform and added dropwise to the ethylene diamine solution over three hours. The reaction mixture was then stirred at RTP for 16 hours. After 16 hours the reaction mixture was washed with brine (3 × 100 mL) and then water (50 mL), before drying with magnesium sulphate. This was filtered to remove the magnesium sulphate and excess solvent was removed under vacuum to give a pale-yellow oil (3.28 g). δ_H (400MHz, $CDCl_3$) 4.97 (1H, s, C(O)NH), 3.14 (2H, q, *J* 5.5, $NHCH_2$), 2.76 (2H, t, J 6.0, CH_2NH_2), 1.42 (9H, s, $C(CH_3)_3$), 1.30 (2H, s, NH_2). δ_c (400MHz, $CDCl_3$) 156.3 (1C, C(O)), 43.5 (1C, $NHCH_2$), 42.0 (1C, CH_2NH_2), 28.5 (3C, $C(CH_3)_3$) - $C(CH_3)_3$ hidden by solvent peak. FTIR (cm^{-1}) – 3357 (amine and amide N-H), 2972, 2931 & 2870 (methyl and methylene), 1684 (amide carbonyl), 1507 (methyl). m/z calculated as 160.12; found for ESI $[M+H]^+$ 161.2 & $[2M+H]^+$ 321.5

Synthesis of biotin diamine t-BOC[3]

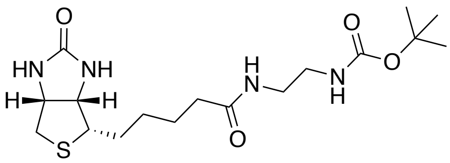

0.253 g (1.04 mmol) of biotin, 0.217 g (1.36 mmol) of diamine *t*-Boc and 0.265g (1.70 mmol) of EDC were added to a mixture of 5 mL methanol and 15 mL acetonitrile under nitrogen. The solution was stirred at 50°C for 5 hours. Excess solvent was removed under vacuum and the crude resuspended in methanol before filtering through a celite plug. The solvent was removed under vacuum and the product purified by silica column chromatography (DCM:MeOH 9:1) using a $KMnO_4$ stain to give a white powder (0.193 g). δ_H (400MHz, MeOD) 7.65-7.55 (1H, m, N*H*C(O)O), 7.45-7.34 (1H, m, CH_2C(O)N*H*), 6.75-6.62 (1H, m, C(O)N*H*CHCH), 6.15-6.07 (1H, m, C(O)N*H*$CHCH_2$), 4.57-4.47 (1H, m, C*H*CH_2(S)), 4.37-4.28 (1H, m, C*H*CH(S)), 3.82 (2H, q, *J* 7, CH_2C(O)NHCH_2), 3.22-3.04 (3H, m, CH_2NHC(O)O & SC*H*), 2.78 (1H, t, *J* 8.5, CHC*H*HC(S)), 2.71 (1H, t, *J* 7, CHCH*H*C(S)), 2.22 (2H, t, J 7.5, CH_2C(O)), 1.72-1.47 (4H, m, C$H_2$$CH_2CH_2$$CH_2$C(O)), 1.43 (9H, s, $OC(CH_3)_3$), 1.30-1.10 (2H, m, C$H_2$$CH_2$$CH_2$C(O)NH). δ_C (400MHz, MeOD) 176.4 (1C, CH_2*C*(O)NH) 166.1 (1C, NHC(O)NH), 80.1 (1C, *C*$(CH_3)_3$), 63.3 (1C, C(O)NH*C*HCH_2), 61.6 (1C, C(O)NH*C*HCH), 56.9 (1C, C(O)NHCH*C*H), 41.03, 41.96 & 40.5 (3C, C(O)NHCH*C*H_2, C(O)NH_2*C*H_2*C*$H_2$$NH_2$), 36.8 (1C, *C*$H_2$C(O)NH), 29.8 & 29.5 (2C, C(*C*H_3)$_3$), 28.8 & 26.8 (2C, SCH*C*H_2*C*H_2). FTIR (cm^{-1}) – 3291 (amine and amide N-H), 2931 & 2864 (methyl and methylene), 1687 & 1647 (amide carbonyls), 1528 (methyl/aromatic). m/z calculated as 386.20; found for ESI $[M+Na]^+$ 409.2 & $[M-H]^-$ 385.2

Synthesis of biotin-NH_2[4]

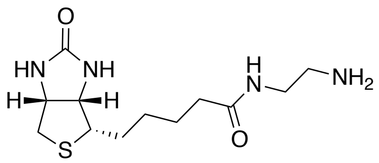

0.1 g (0.26 mmol) of biotin diamine *t*-Boc was added to 6 mL of DCM containing 1.5 mL of TFA. This was stirred for 2 hours at RTP. The reaction mixture solvent was removed under vacuum and the crude solid dissolved in 50 mL of water before washing with diethyl ether (3× 50 mL). The aqueous layer solvent was removed under vacuum and dried to give a clear colourless oil (0.08 g). δ_H (400MHz, D_2O) 4.62 (1H, dd, *J* 7.5, 5.0 C*H*CH_2(S)), 4.44 (1H, dd, *J* 8, 4.5, C*H*CH(S)), 3.51 (2H, t, *J* 6, C(O)NHC*H_2*), 3.39 - 3.32 (1H, m, CHC*H*S), 3.15 (2H, t, *J* 6, C*H_2*NH_2), 3.01 (1H, dd, *J* 13, 5, CHCH*H*S), 2.89 - 2.69 (1H, m, CHC*H*HS), 2.31 (2H, t, *J* 7.5 C*H_2*C(O)NH), 1.81 - 1.52 (4H, m, SCHC*H_2*CH_2C*H_2*), 1.49 - 1.36 (2H, m, SCHCH_2C*H_2*CH_2). δ_C (400MHz, D_2O) 177.9 (1C, CH_2*C*(O)NH), 62.1 (1C, *C*HCHS), 60.3 (1C, *C*HCH_2S), 55.3 (1C, CH*C*HS), 39.6 (1C, CH*C*H_2S) 39.1 (1C, *C*$H_2$$NH_2$), 36.7 (1C, C(O)NH*C*$H_2$), 35.3 (1C, *C*$H_2$C(O)NH$CH_2$), 27.9 & 27.6 (2C, S*C*$H_2$$CH_2$*C*$H_2$), 24.8 (1C, S$CH_2$*C*$H_2$$CH_2$). FTIR ($cm^{-1}$) – 3390, 3272 (amine and amide N-H), 1685 & 1662 (amide carbonyls). m/z calculated as 286.39; found for ESI $[M+H]^+$ 287.1 $[M+Na]^+$ 309.1

Citrate-Stabilized 16 nm Gold Nanoparticle Synthesis[5]

To 500 mL of water was added 0.163 g (0.414 mmol) of gold(III) chloride trihydrate, the mixture was heated to reflux and 14.6 mL of water containing 0.429 g (1.46 mmol) of sodium citrate tribasic dihydrate was added. The reaction was allowed to reflux for 30 minutes before cooling to room temperature over 3 hours. The solution was centrifuged at 13 krpm for 30 minutes and the pellet resuspended in 40 mL of water to give an absorbance at 520 nm of ~1Abs.

Gold Nanoparticle Polymer Coating Functionalization – 16 nm

10 mg of polymer was agitated overnight with 10 mL of 16 nm AuNPs ~1Abs at UV_{max}. The solution was centrifuged at 13 krpm for 30 minutes and the pellet resuspended in 10mL of water, the solution was centrifuged again at 13 krpm for 30 minutes and the pellet resuspended in 1 mL aliquots and centrifuged at 14.5 krpm for 10 minutes. The pellets were combined into a 1 mL solution with an absorbance at 520 nm of ~10 Abs.

Gold Nanoparticle Polymer Coating Functionalization – 40 nm

10 mg of polymer was agitated overnight with 10 mL of 40 nm AuNPs ~1Abs at UV_{max}. The solution was centrifuged at 6 krpm for 10 minutes and the pellet resuspended in 10mL of water, the solution was centrifuged again at 6 krpm for 10 minutes and the pellet resuspended in 1 mL aliquots and centrifuged at 6 krpm for 10 minutes. The pellets were combined into a 1 mL solution with an absorbance at UV_{max} of ~10 Abs.

Lateral Flow Strip Production, Running and Analysis Protocols

The procedure to produce flow-through and lateral flow devices was identical, apart from the deposition of the analyte directly onto the nitrocellulose (flow-through), versus application of tests lines onto the nitrocellulose (lateral flow). This is a truncated protocol from Baker *et al.*, provided for clarity.[6]

Protocol for Manufacturing Lateral Flow Strips

Backing cards were cut to size by removal of 20 mm using a guillotine. Nitrocellulose was added to the backing card by attaching the plastic backing of the nitrocellulose to the self-adhesive on the card. The wick material was then added to the backing card so it overlaps with the nitrocellulose by ~5 mm. The lateral flow strips were cut to size of width 2-3 mm.

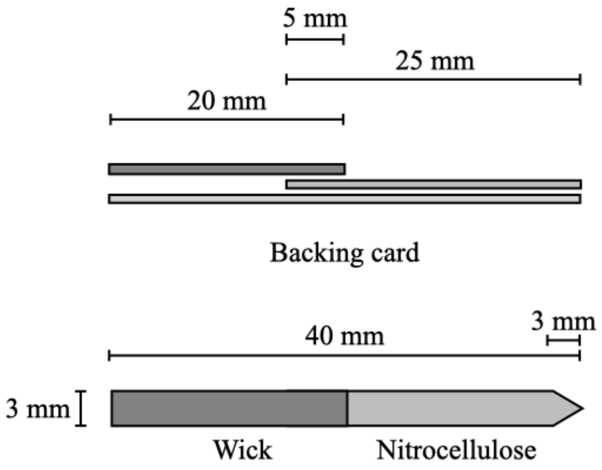

Figure S2. Lateral flow strip dimensions

Protocol for Test Line Addition to the Lateral Flow Strips

1 µL of the test line solution was added to the test strip using a micropipette fitted with 10 µL tip, the test line was spotted ~1 cm from the non-wick end of the strip. The strips were dried at 37 °C in an oven for 30 minutes. The tests strips were allowed to cool to room temperature before testing.

Protocol for Running Lateral Flow Test Without Target Analyte in Buffer

The running buffer of total volume 50 µL was made as follows; 5 µL AuNPs (OD10), 5 µL lateral flow assay buffer – 10 × HEPES buffer, 40 µL water. The running solution was then agitated on a roller for 5 minutes. 45 µL of this solution was added to a 0.2 mL PCR tube, standing vertically.

A small "v" (~3 mm) was cut into the test strips at the non-wick end and the strips added to the PCR tubes, so they protrude from the top and the immobile phase (1 cm from non-wick end) is not below the solvent line. There was one test per tube. All tests were run in triplicate.

The tests were run for 20 minutes before removal from the tubes. The test strips were allowed to dry at room temperature for ~5 minutes. The test strips were mounted test-face down onto a clear and colourless piece of acetate sheeting.

The *Protocol for Running Lateral Flow Test Without Target Analyte in Buffer* was used for the flow-through assays as the target analyte is deposited on the nitrocellulose as a "test line" i.e. the analyte is not in the running buffer.

Protocol for Running Lateral Flow Test with Target Analyte in Buffer

The running buffer of total volume 50 µL was made as follows; 5 µL AuNPs (OD10), 5 µL lateral flow assay buffer – 10 × HEPES buffer, 40 µL of water - x µL, where x is the volume of target analyte added to make the required concentration of the lectin. The running solution was then agitated on a roller for 5 minutes. 45 µL of this solution was added to a 0.2 mL PCR tube, standing vertically.

A small "v" (~3 mm) was cut into the test strips at the non-wick end and the strips added to the PCR tubes, so they protrude from the top and the immobile phase (1 cm from non-wick end) is not below the solvent line. There was one test per tube. All tests were run in triplicate.

The tests were run for 20 minutes before removal from the tubes. The test strips were allowed to dry at room temperature for ~5 minutes. The test strips were mounted test-face down onto a clear and colourless piece of acetate sheeting.

Standard Protocol for Lateral Flow Strip Analysis

The acetate sheets were scanned using a Kyocera TASKalfa 5550ci printer to a pdf file that was converted to a jpeg, scans were taken within 1 hour of strip drying. The jpeg was analyzed in ImageJ 1.51[1] using the plot profile function to create a data set exported to Microsoft Excel for Mac. The data was exported to Origin 2019 64Bit and trimmed to remove pixel data not from the strip surface. The data was aligned and averaged (mean). The data was then reduced by number of groups to 100 data points (nitrocellulose and wick) and plotted as Grey value (scale) vs Relative distance along the 100 data points.

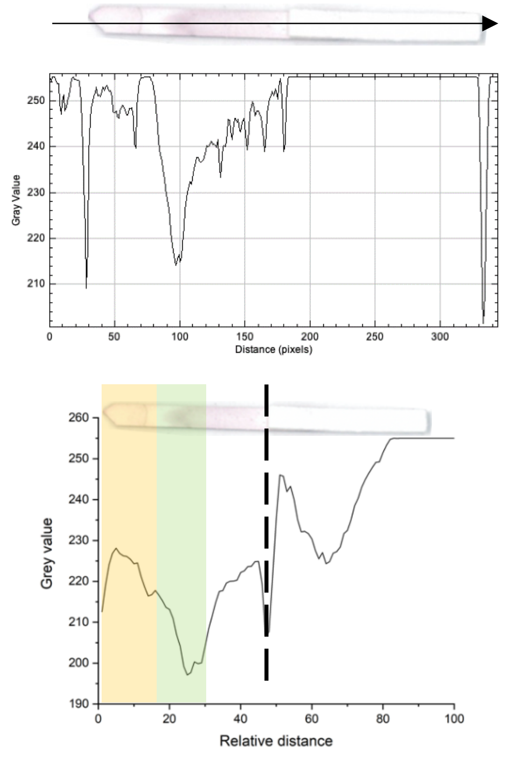

Figure S3. Representative dipstick (Top), raw grey value plot (Middle) and processed grey value plot (Bottom)

Lateral Flow Assay Buffer - 10× HEPES buffer (10% PVP_{400}) in 100 mL H_2O

2.38 g (100 mmol.dm^{-3}) of HEPES, 8.77 g (1.50 mol.dm^{-3}) of NaCl, 0.011 g (1.0 mmol.dm^{-3}) of $CaCl_2$, 0.8 g (0.8% w/v., 123 mmol.dm^{-3}) of NaN_3, 0.5 g (0.5% w/v., 4.07 mmol.dm^{-3}) of Tween-20 and 10 g (10% w/v.) of poly(vinyl pyrrolidone)$_{400}$ (PVP_{400}, Average Mw ~40,000) were dissolved in 100 mL of water. The buffer was not pH adjusted.

Intensity Calculations

The average background was determined by calculating the mean grey value of points between 0 - 60 relative distance units, subtracted from 255 (the grey value of clean nitrocellulose); excluding points from aggregation at the solvent front, points contributing to the signal peak and points in the wick. This average background value was subtracted from the lowest grey value of the signal peak (subtracted from 255) to give intensity.

Signal-to-Noise Calculations

The signal (intensity of test) was then divided by the noise (intensity of control) value to give a signal-to-noise value.

Additional Data and Figures

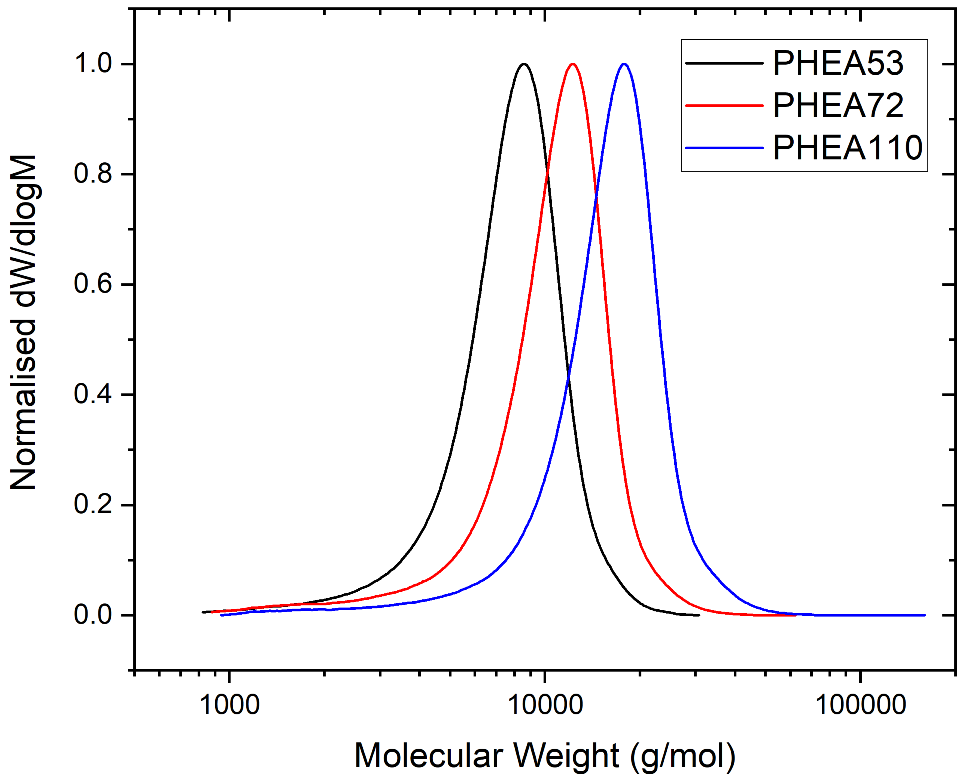

Figure S4. Normalized size exclusion chromatography RI molecular weight distributions of telechelic PHEA obtained in DMF versus PMMA standards.

Table S1. PHEA Polymers prepared for use as AuNP coatings

Polymer	[M]:[CTA]	$M_{n(theo)}$ [g.mol^{-1}]^{a}	$M_{n(SEC)}$ [g.mol^{-1}]^{b}	$M_{n(NMR)}$ [g.mol^{-1}]^{c}	$Đ_M$^{b}
PHEA53	28	3800	6600	6000	1.24
PHEA72	40	5100	8900	8600	1.28
PHEA110	70	8600	13000	14000	1.27

a) Determined from feed ratio of monomer to chain transfer agent; b) Calculated against poly(methyl methacrylate) standards using 5mM NH_4BF_4 in DMF as eluent; c) Determined from ^{1}H NMR end-group analysis.

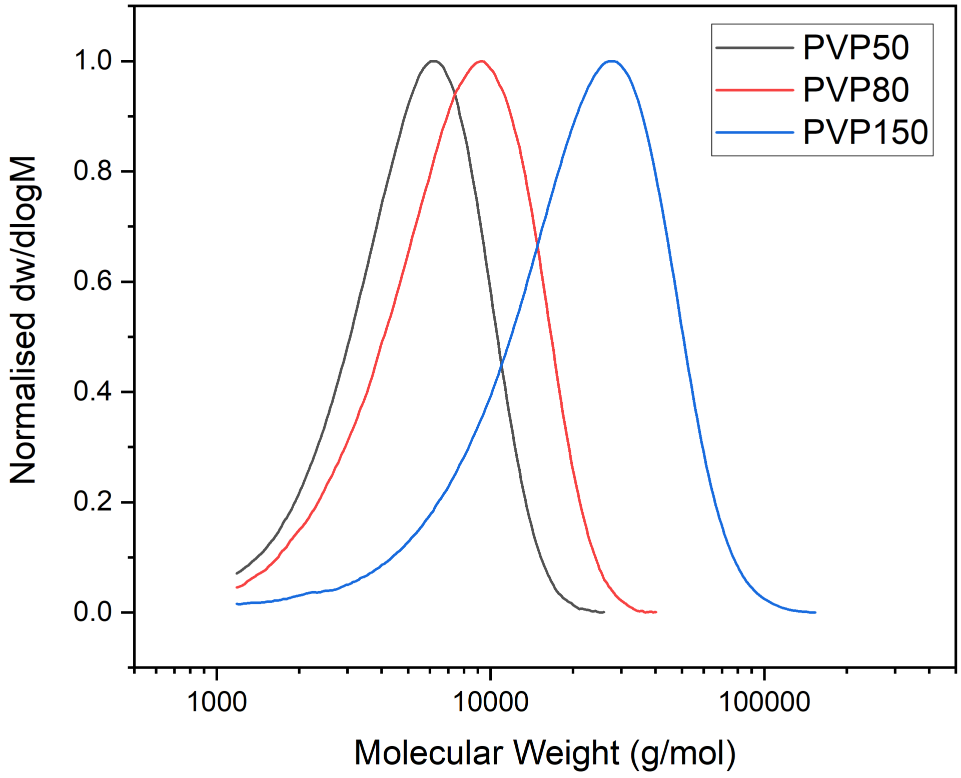

Figure S5. Normalized size exclusion chromatography RI molecular weight distributions of PVP obtained in DMF versus PMMA standards.

Table S2. PVP Polymers for use as test lines

Polymer	[M]:[CTA]	$M_{n(theo)}$ [g.mol^{-1}][a]	$M_{n(SEC)}$ [g.mol^{-1}][b]	$M_{n(NMR)}$ [g.mol^{-1}][c]	$Đ_M$[b]
PVP50	200	22500	4500	5900	1.33
PVP80	300	33600	6000	9200	1.47
PVP150	500	55900	15100	17000	1.72

a) Determined from feed ratio of monomer to chain transfer agent assuming 100 % conversion; b) Calculated against poly(methyl methacrylate) standards using 5mM NH_4BF_4 in DMF as eluent; c) Determined from ^1H NMR end-group analysis.

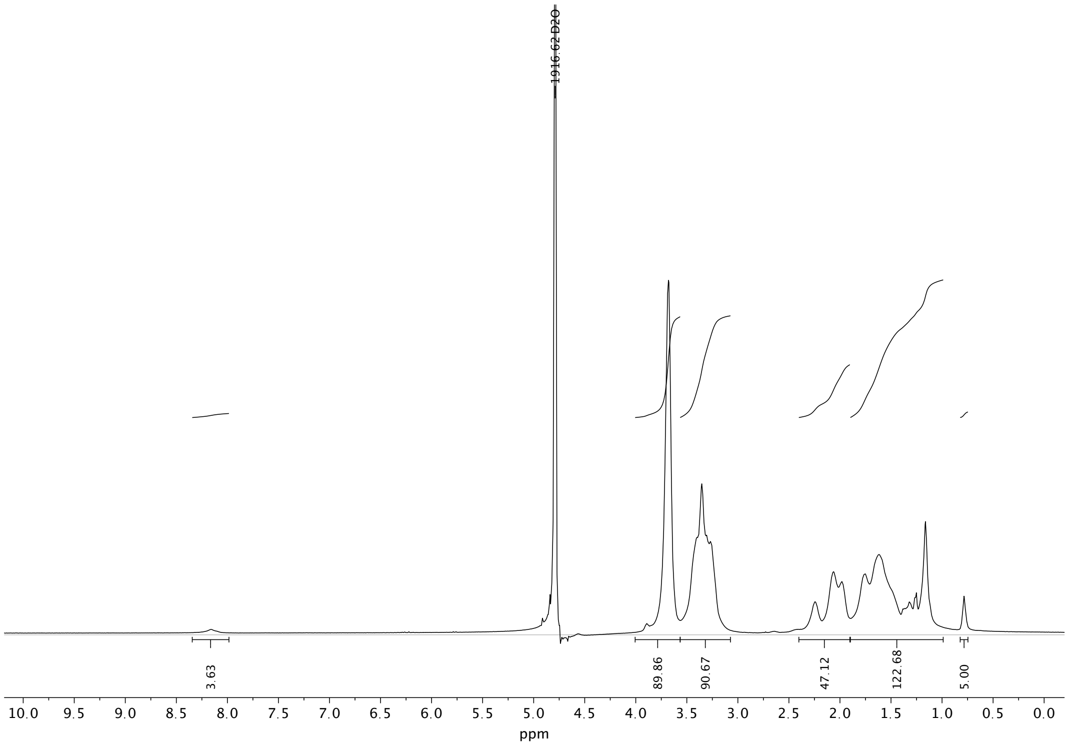

Figure S6. ^{1}H NMR spectrum of PHEA53

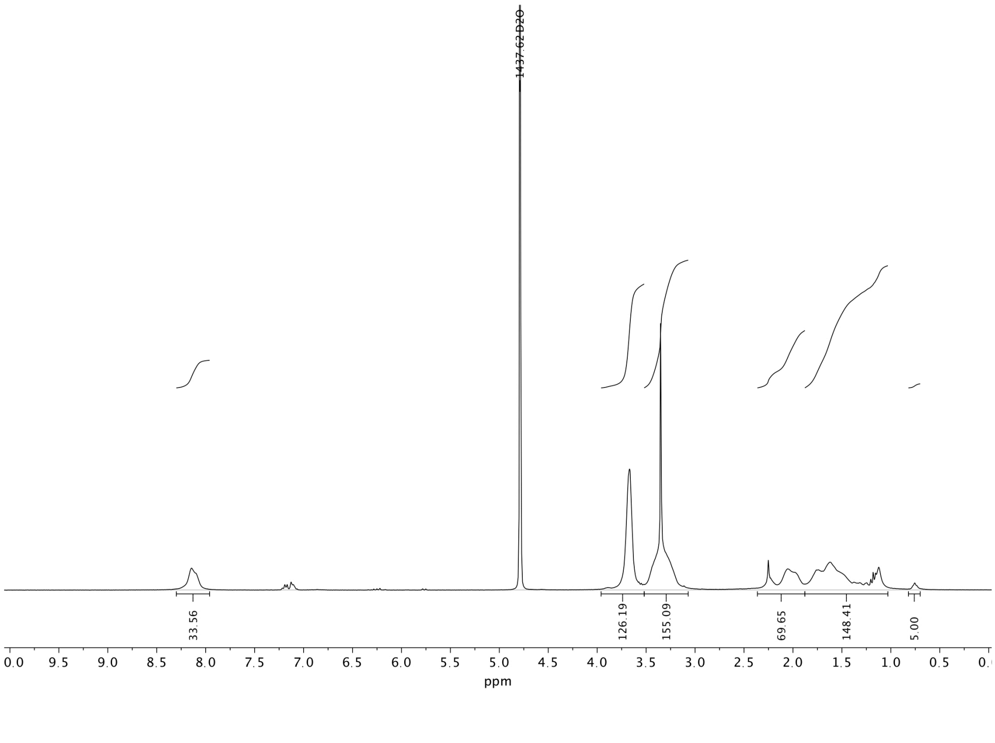

Figure S7. ^{1}H NMR spectrum of PHEA72

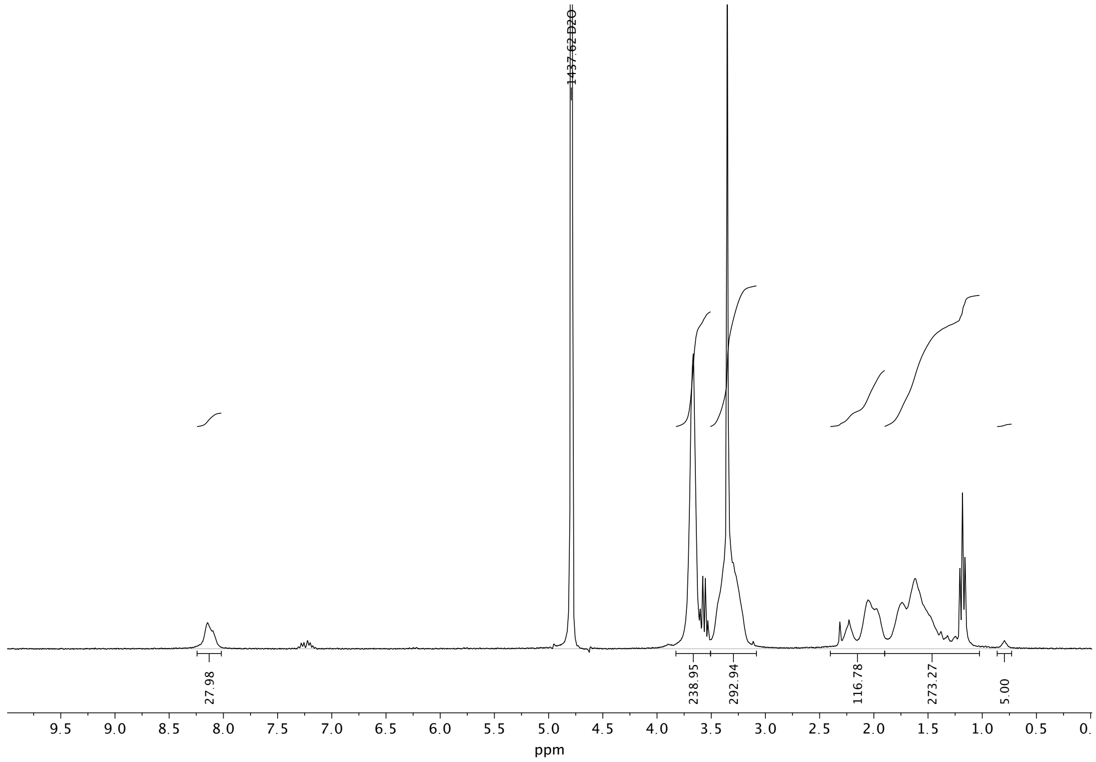

Figure S8. ^{1}H NMR spectrum of PHEA110

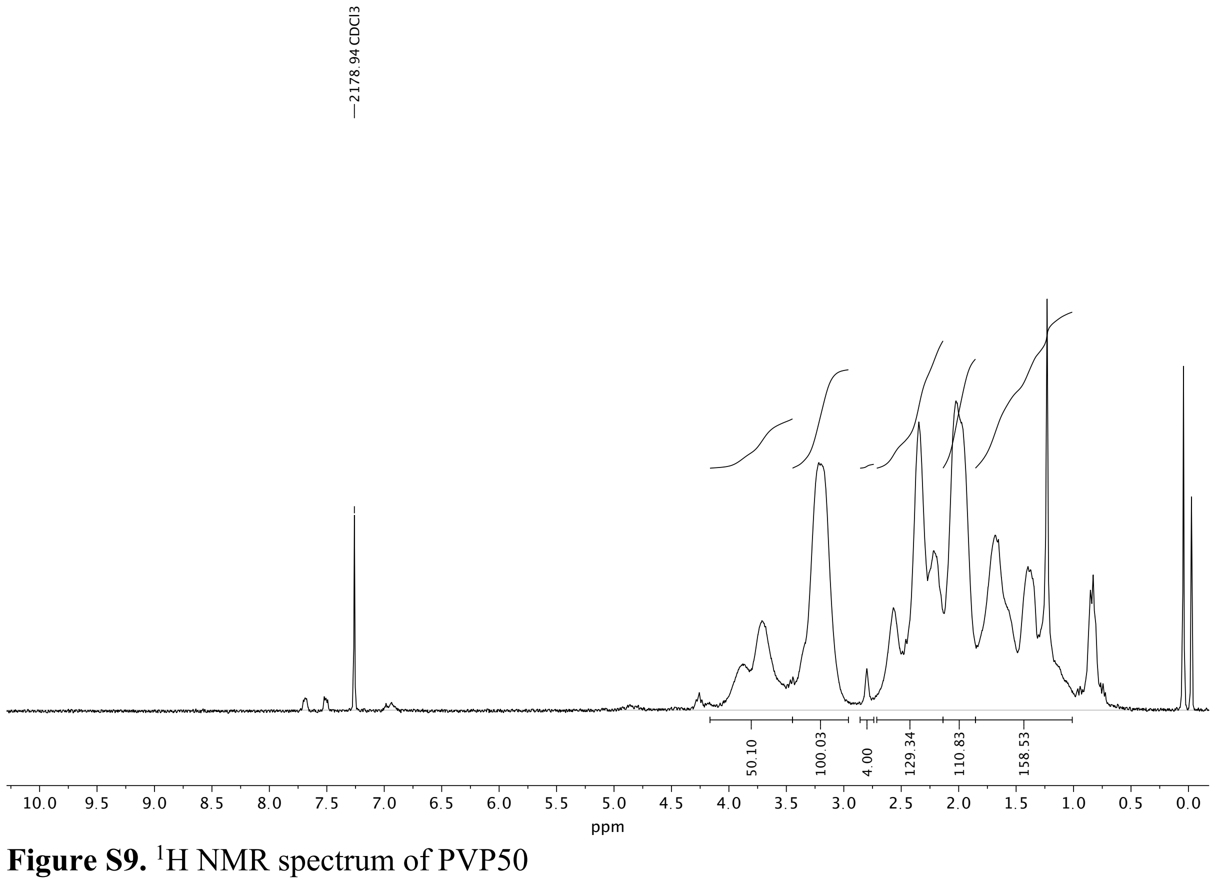

Figure S9. ^{1}H NMR spectrum of PVP50

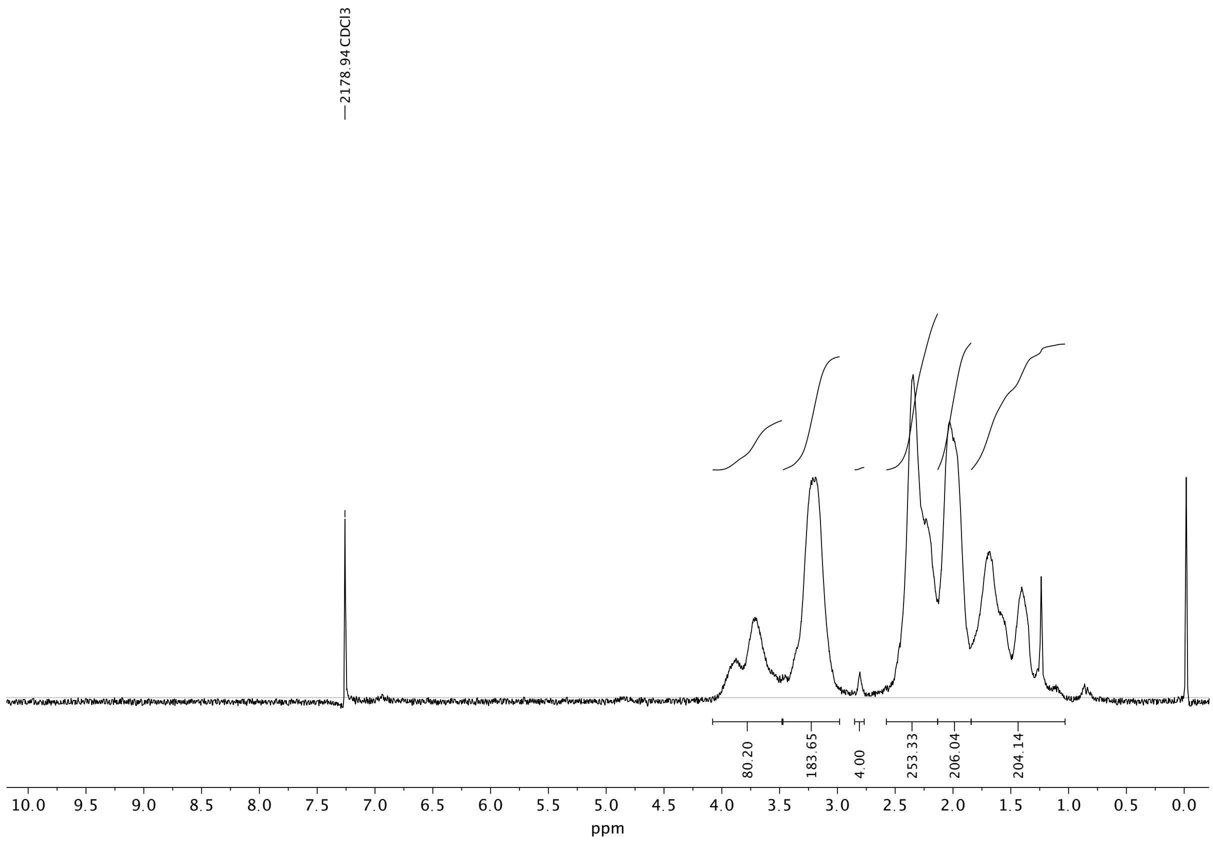

Figure S10. ^{1}H NMR spectrum of PVP80

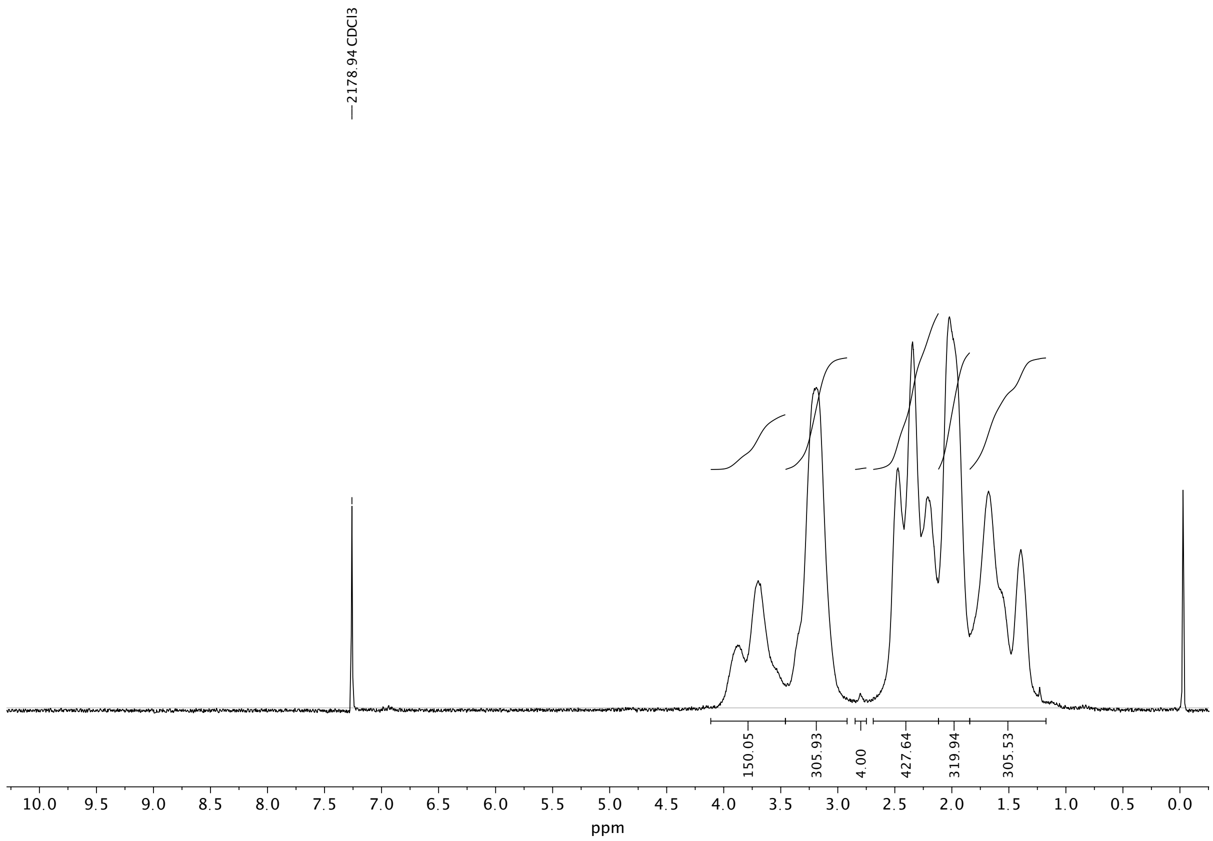

Figure S11. ^{1}H NMR spectrum of PVP150

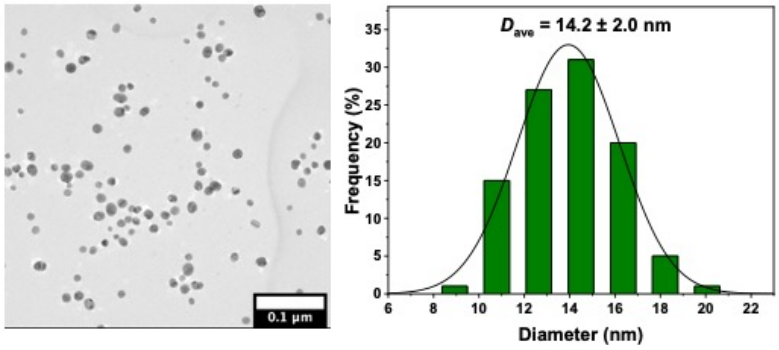

Figure S12. TEM images (Left) and histograms (right) of citrate stabilized 16nm AuNPs. Histograms from analysis of over 100 particles.

AuNP Data

DLS and UV-vis Data

Table S3. Nanoparticles Synthesized and Characterized

**Particle**	**λ_{SPR}[a] (nm)**	**$\lambda_{SPR}/\lambda_{450}$[a] (nm)**	**DLS[b] (nm)**
$AuNP_{16}$	518	1.56	21 ±1
Gal-$PHEA_{72}$@$AuNP_{16}$	524	1.49	38 ±3
Biotin-$PHEA_{53}$@$AuNP_{16}$	545	1.35	113 ±2
Biotin-$PHEA_{72}$@$AuNP_{16}$	545	1.35	120 ± 1
Biotin-$PHEA_{110}$@$AuNP_{16}$	570	1.28	152 ±5
$AuNP_{40}$	527	0.20	32 ±11
Biotin-$PHEA_{53}$@$AuNP_{40}$	Unstable	Unstable	Unstable
Biotin-$PHEA_{72}$@$AuNP_{40}$	530	2.05	73 ±1
Biotin-$PHEA_{110}$@$AuNP_{40}$	528	2.03	59 ±1

[a]Maximum absorption wavelength from the surface plasmon resonance band of the particles and characteristic ratio. [b]Diameter from dynamic light scattering ± standard error from three measurements.

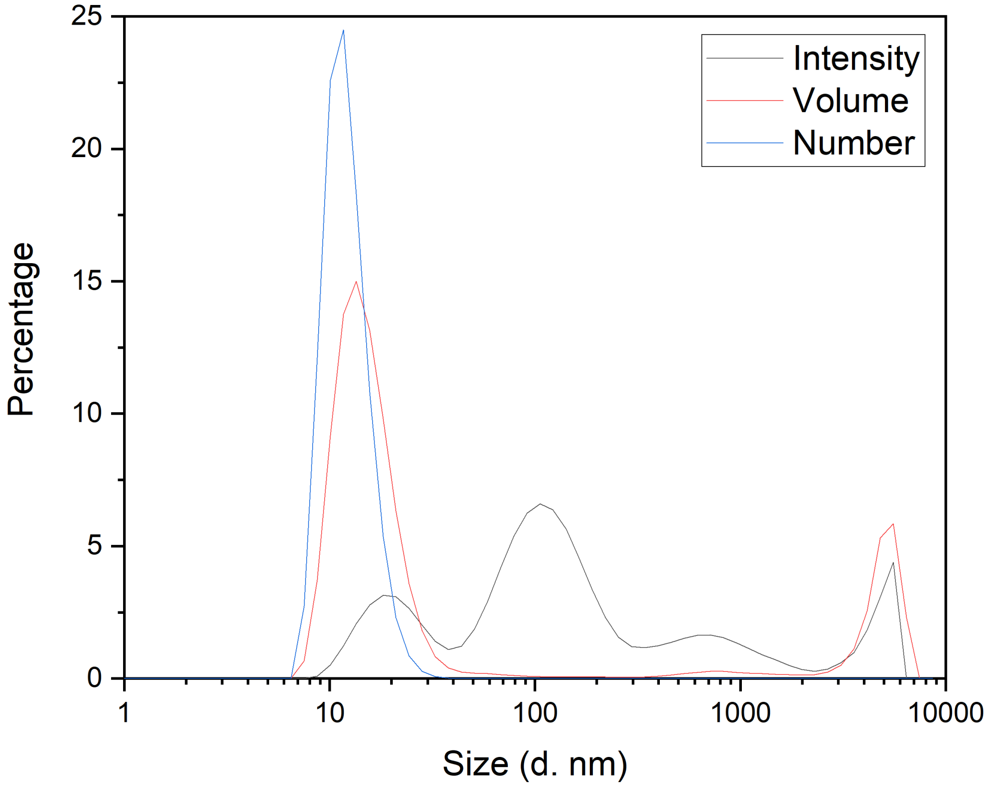

Figure S13. DLS data for citrate-stabilized 16nm AuNP

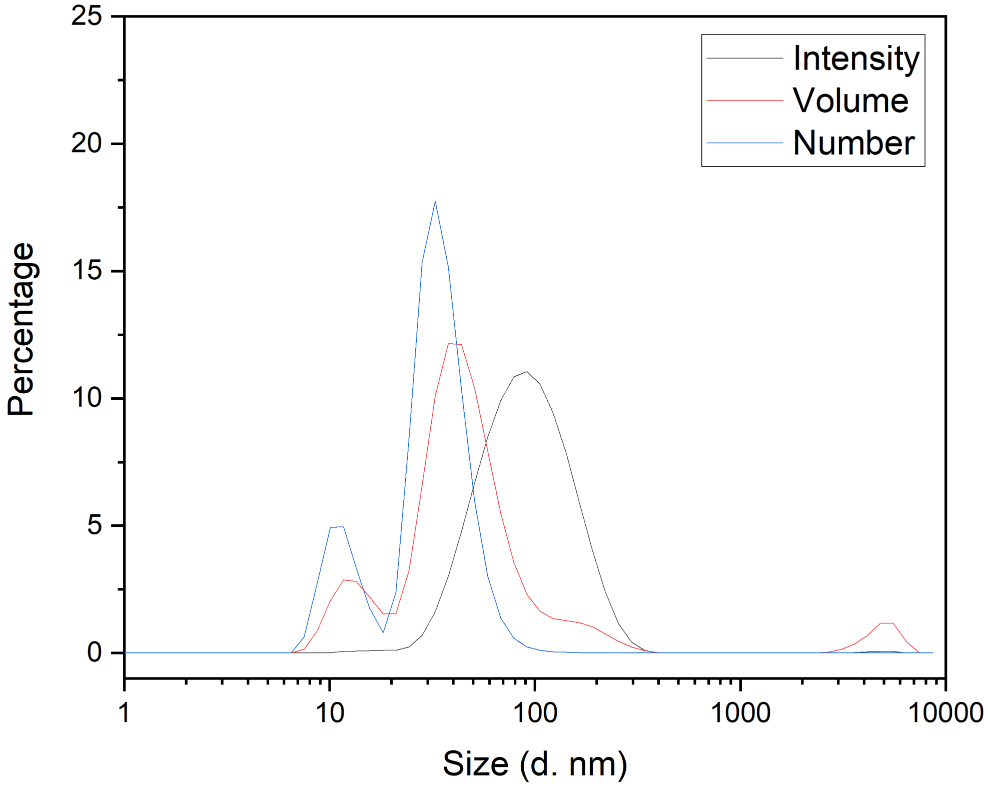

Figure S14. DLS data for citrate-stabilized 40nm AuNP

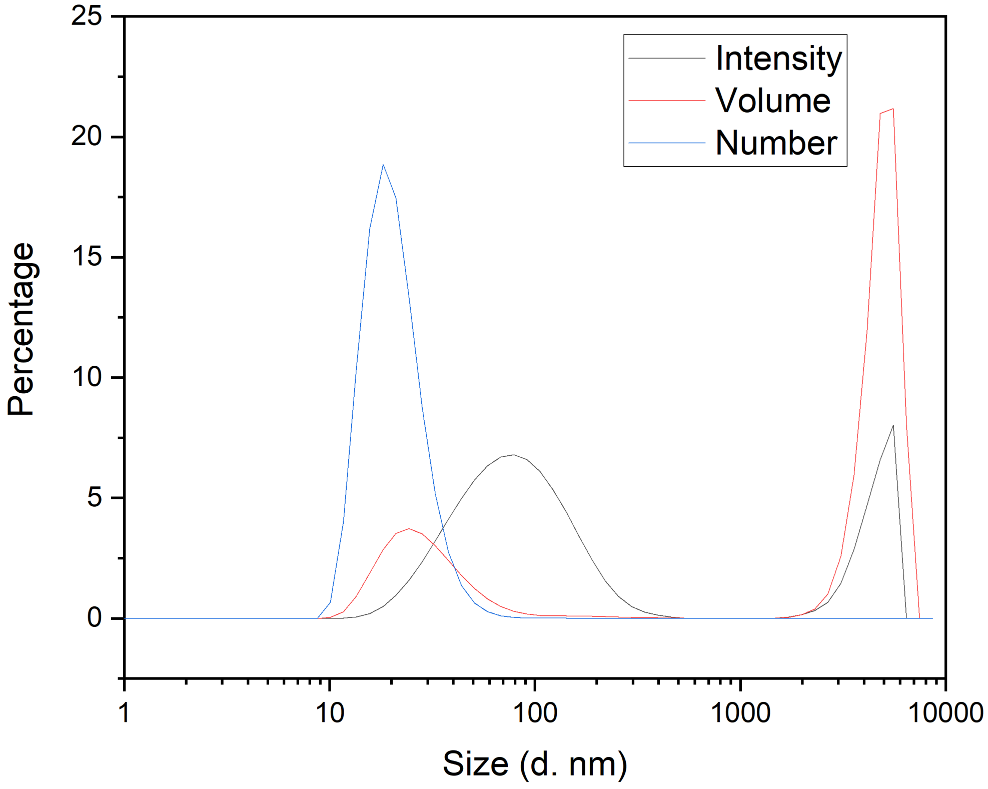

Figure S15. DLS data for Gal-PHEA$_{72}$@AuNP$_{16}$

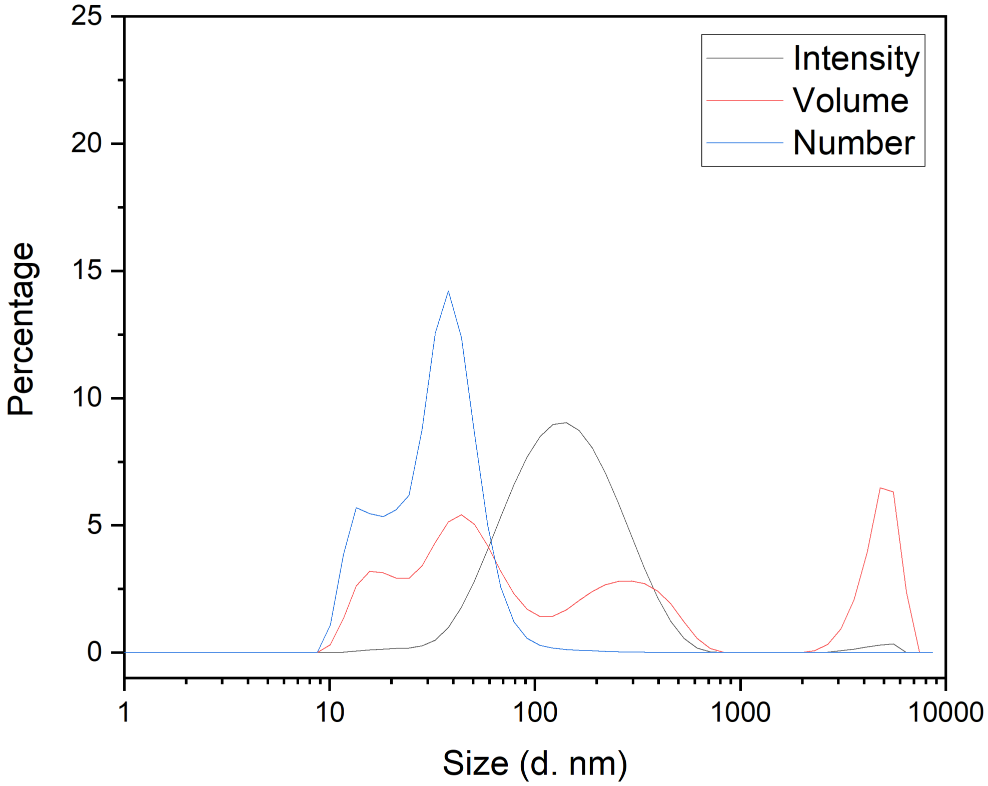

Figure S16. DLS data for Biotin-PHEA$_{53}$@AuNP$_{16}$

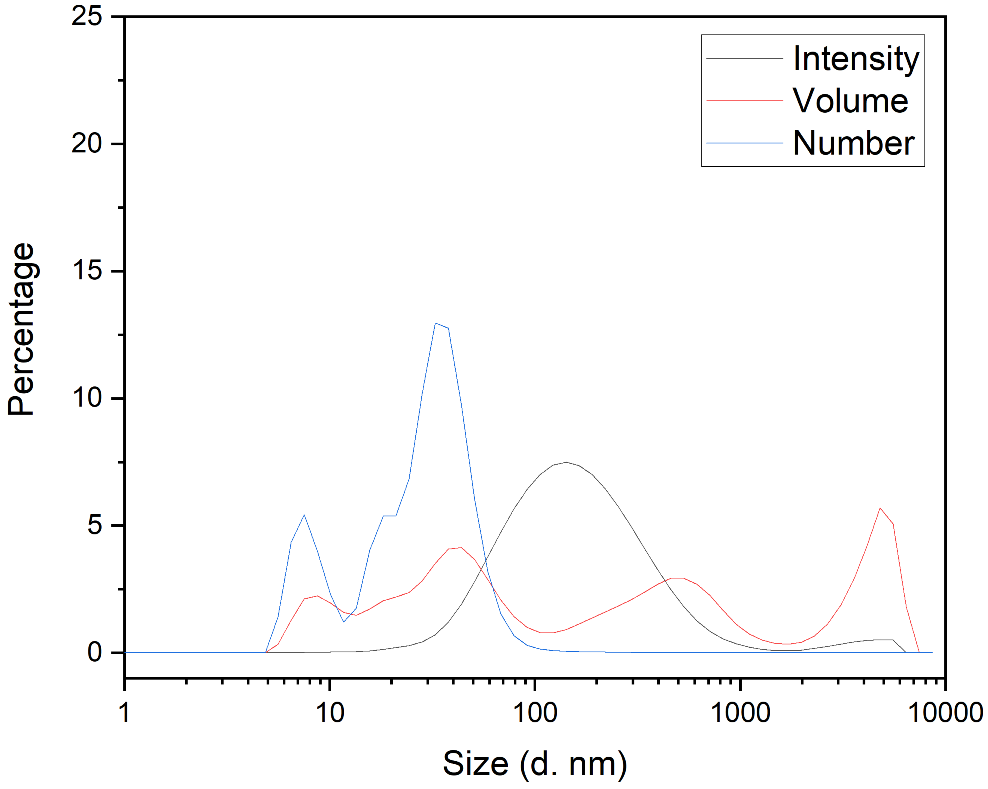

Figure S17. DLS data for Biotin-PHEA$_{72}$@AuNP$_{16}$

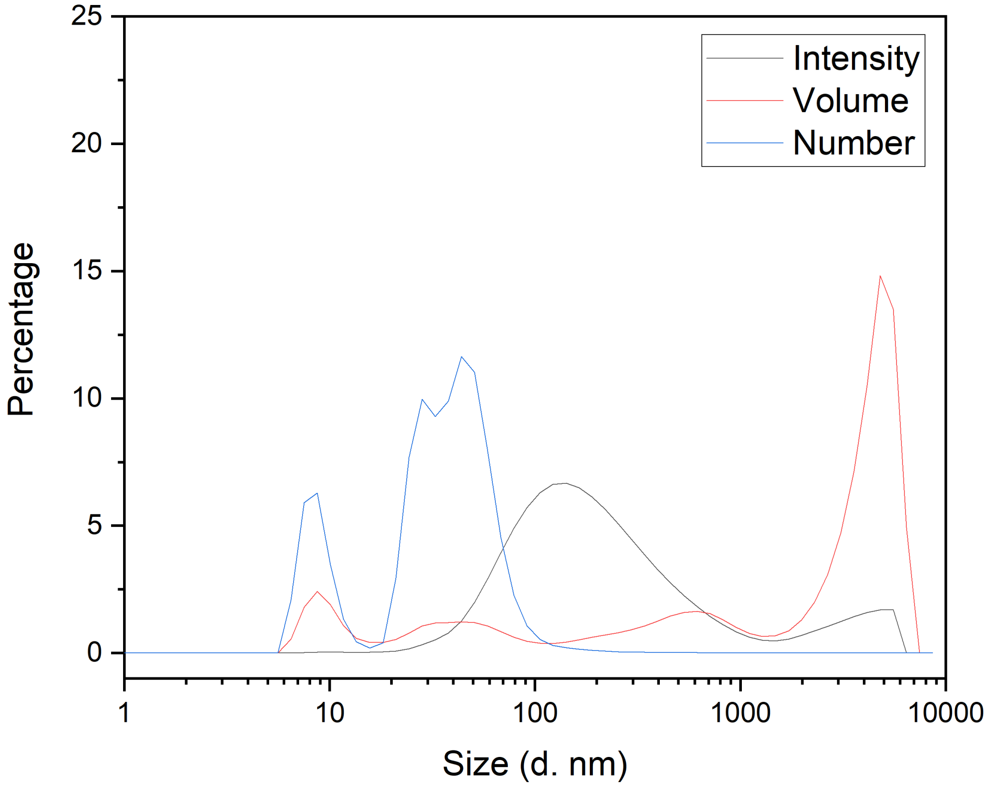

Figure S18. DLS data for Biotin-PHEA$_{110}$@AuNP$_{16}$

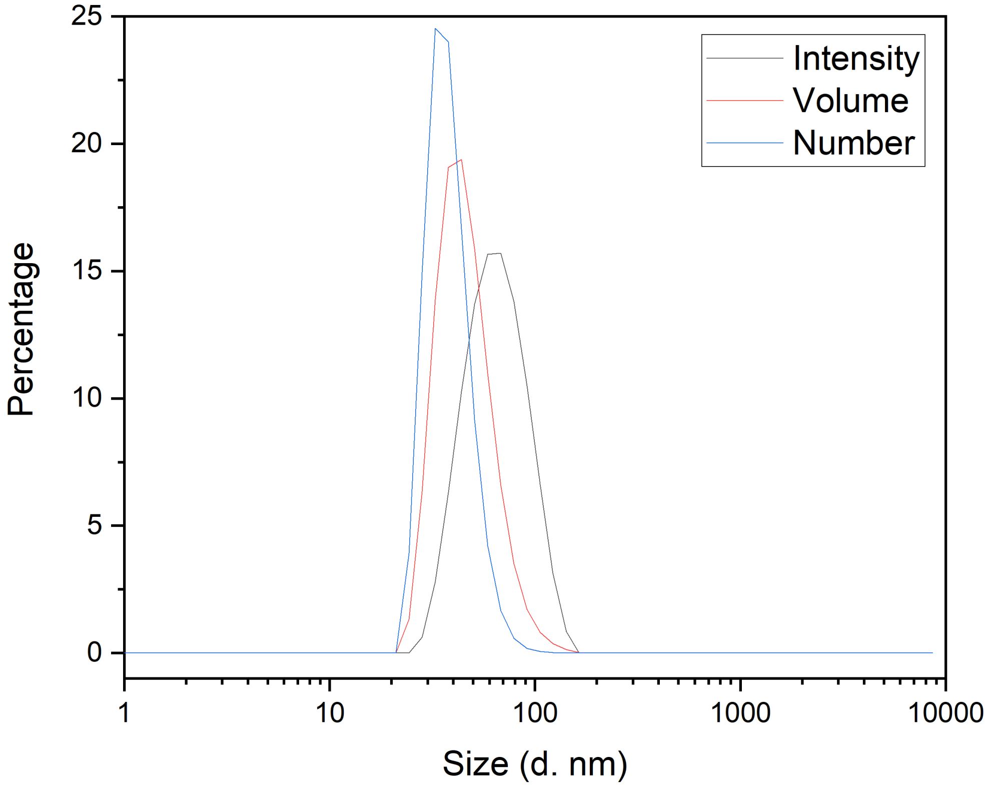

Figure S19. DLS data for Biotin-PHEA$_{72}$@AuNP$_{40}$

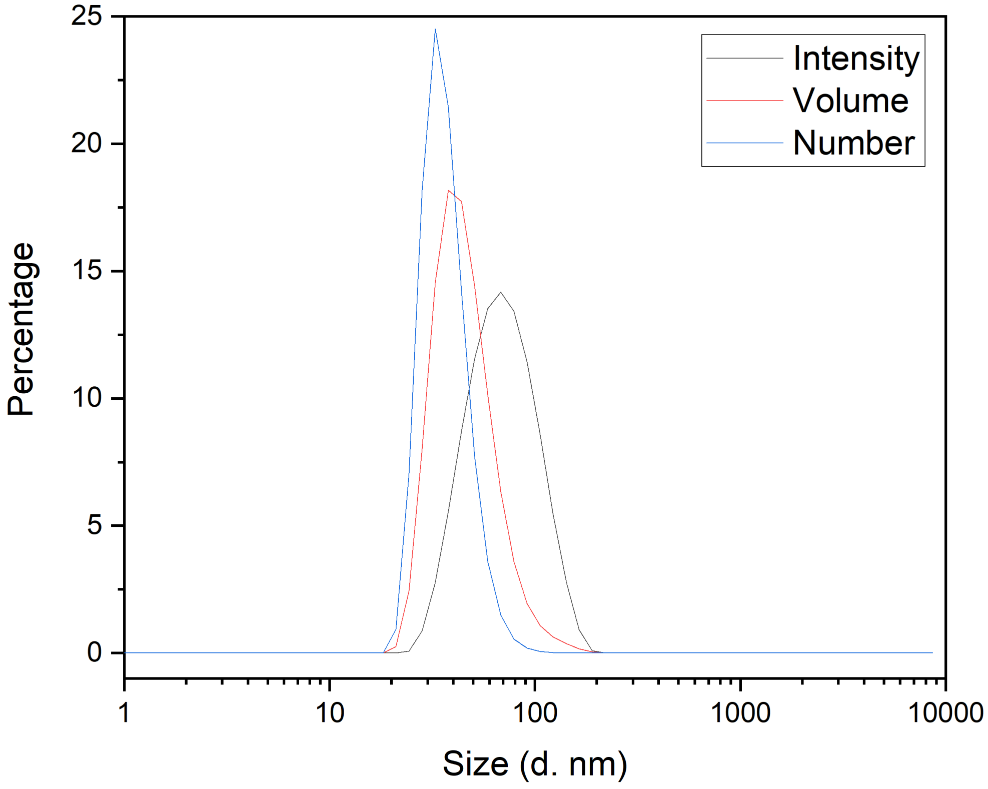

Figure S20. DLS data for Biotin-$PHEA_{110}$@$AuNP_{40}$

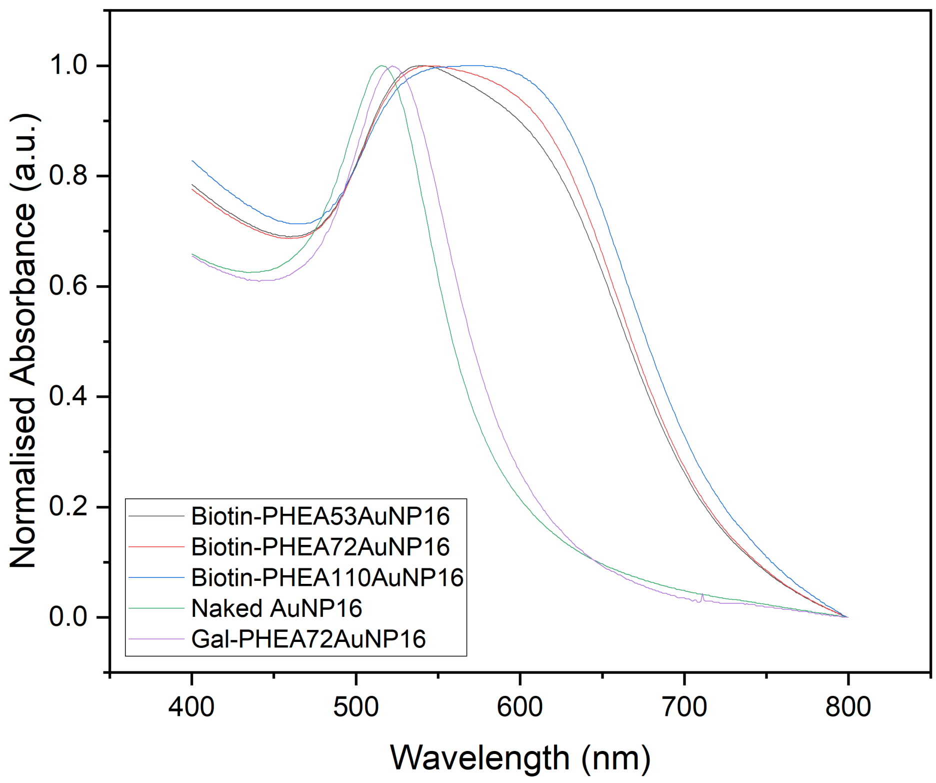

Figure S21. UV-vis data for citrate-stabilized $AuNP_{16}$, Biotin-$PHEA_x$@$AuNP_{16}$ and Gal-$PHEA_{72}$@$AuNP_{16}$

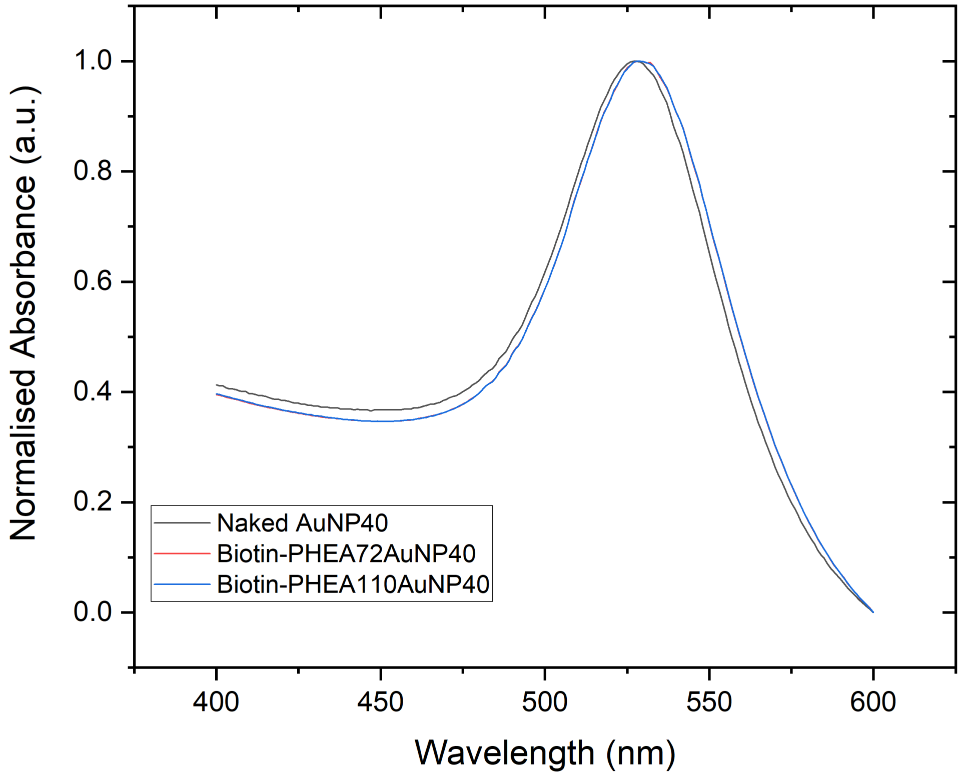

Figure S22. UV-vis data for citrate-stabilized $AuNP_{40}$ and Biotin-$PHEA_x$@$AuNP_{40}$

Flow-Through Dipsticks and Analysis – Biotin and Streptavidin

PVP Test line (20 mg/mL)	Streptavidin-AuNP40	Gal-PHEA72AuNP16
PVP50-Biotin	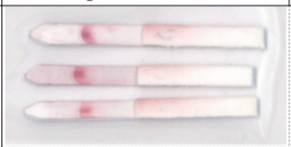	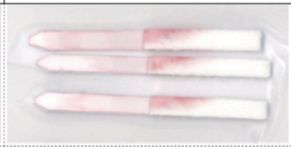
PVP80-Biotin	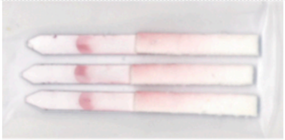	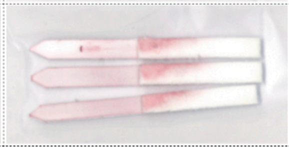
PVP150-Biotin	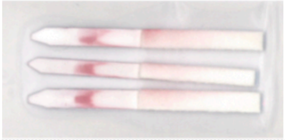	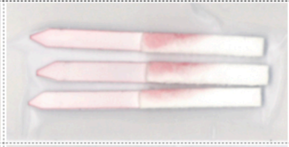
PVP50	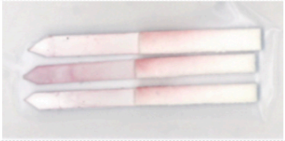	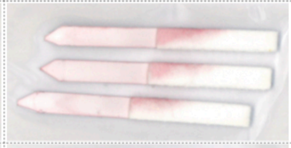
PVP80	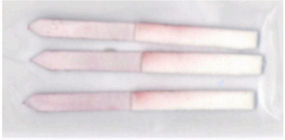	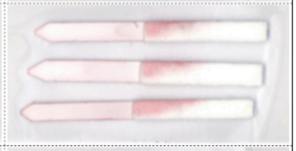
PVP150	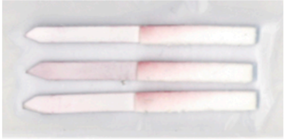	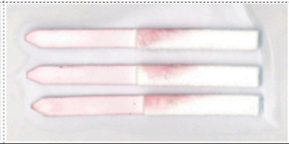

Table S4. Scans of lateral flow strips using test lines of PVP and PVP-Biotin (20 mg/mL) versus either Streptavidin-coated $AuNP_{40}$ or Gal-$PHEA_{72}$@$AuNP_{16}$

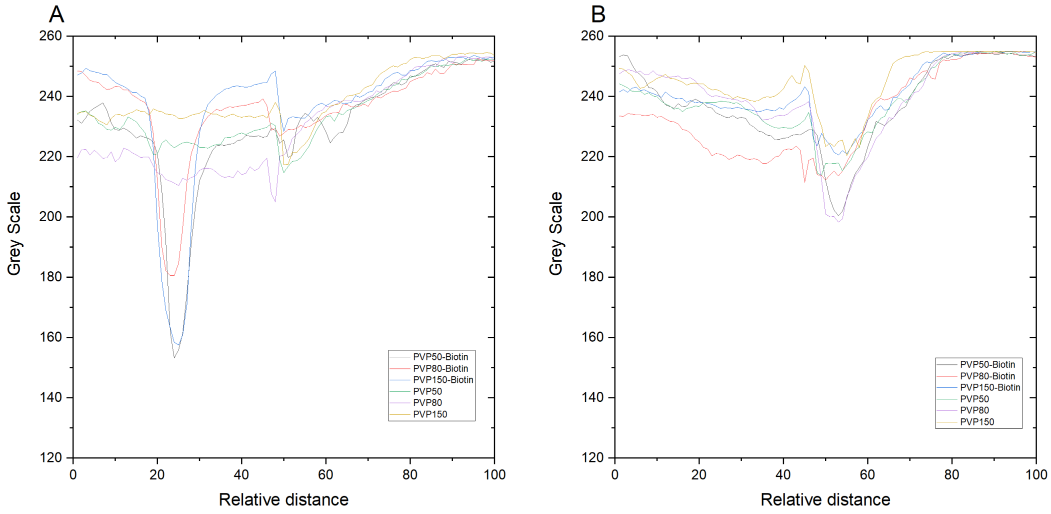

Figure S23. Image analysis of scanned lateral flow strips using test lines of PVP and PVP-Biotin (20 mg/mL) versus either A) Streptavidin-coated $AuNP_{40}$ or B) Gal-$PHEA_{72}$@$AuNP_{16}$

PVP Test line (10 mg/mL)	Streptavidin-AuNP40	Gal-PHEA72AuNP16
**PVP50-Biotin**	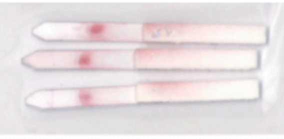	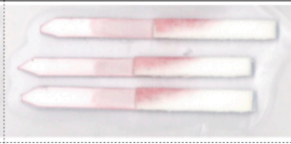
**PVP80-Biotin**	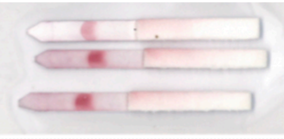	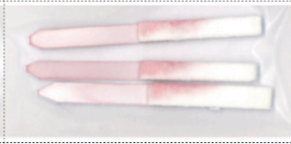
**PVP150-Biotin**	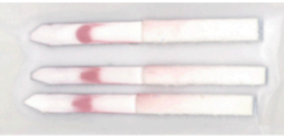	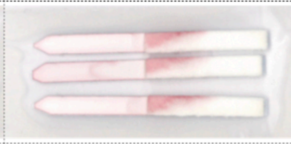
**PVP50**	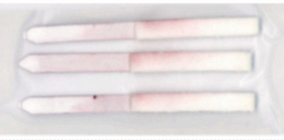	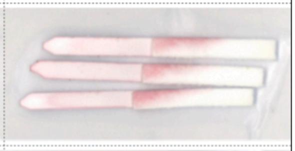
**PVP80**	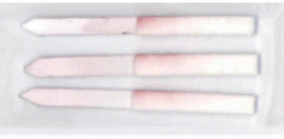	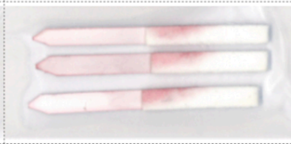
**PVP150**	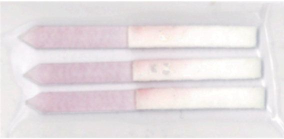	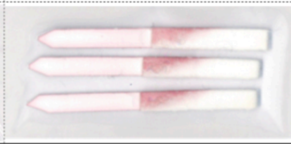

Table S5. Scans of lateral flow strips using test lines of PVP and PVP-Biotin (10 mg/mL) versus either Streptavidin-coated $AuNP_{40}$ or Gal-$PHEA_{72}$@$AuNP_{16}$

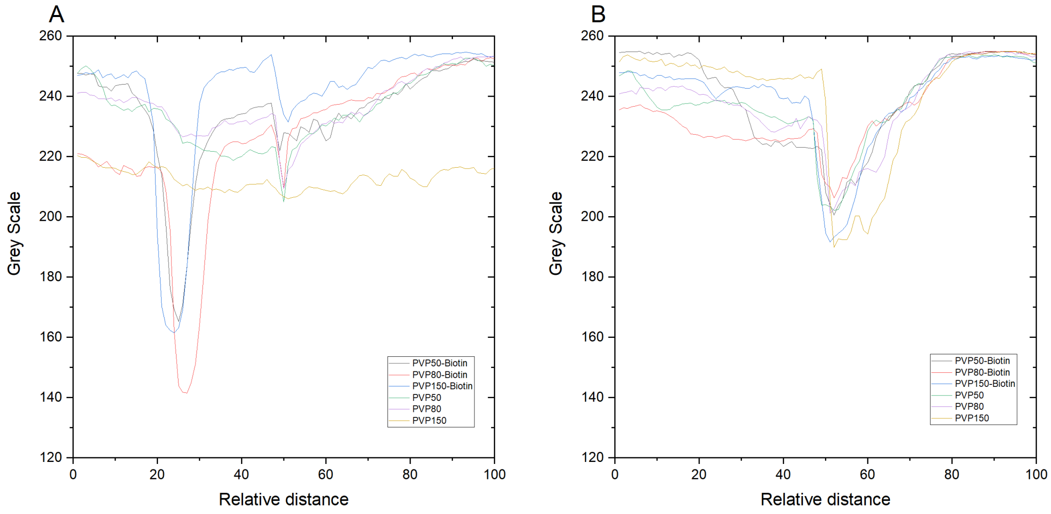

Figure S24. Analysis of scanned lateral flow strips using test lines of PVP and PVP-Biotin (10 mg/mL) versus either A) Streptavidin-coated $AuNP_{40}$ or B) Gal-$PHEA_{72}$@$AuNP_{16}$

PVP Test line (1 mg/mL)	Streptavidin-AuNP40	Gal-PHEA72AuNP16
**PVP50-Biotin**	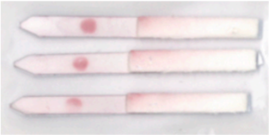	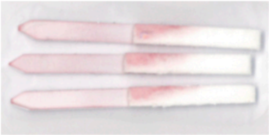
**PVP80-Biotin**	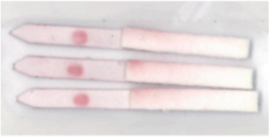	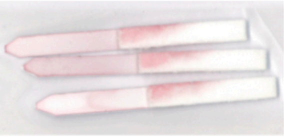
**PVP150-Biotin**	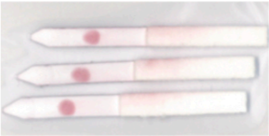	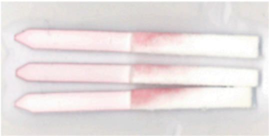
**PVP50**	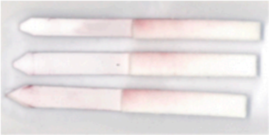	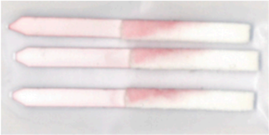
**PVP80**	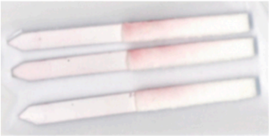	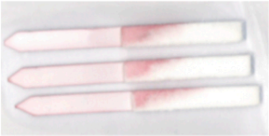
**PVP150**	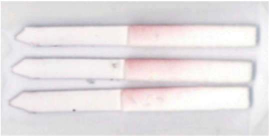	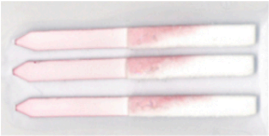

Table S6. Scans of lateral flow strips using test lines of PVP and PVP-Biotin (1 mg/mL) versus either Streptavidin-coated $AuNP_{40}$ or Gal-$PHEA_{72}$@$AuNP_{16}$

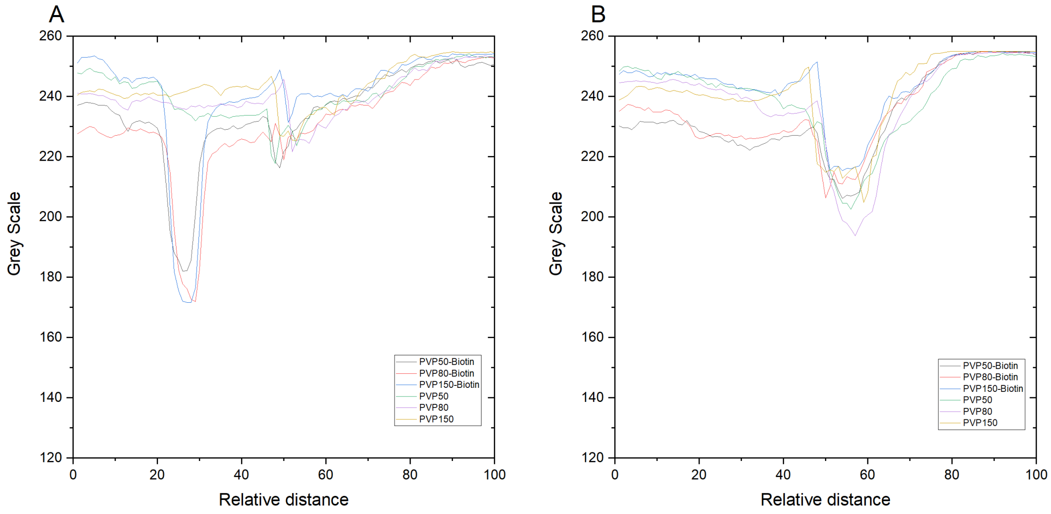

Figure S25. Analysis of scanned lateral flow strips using test lines of PVP and PVP-Biotin (1 mg/mL) versus either A) Streptavidin-coated $AuNP_{40}$ or B) Gal-$PHEA_{72}$@$AuNP_{16}$

Lateral Flow Dipsticks and Analysis – Biotin and Streptavidin

**PVP50-Biotin Test line (10 mg/mL)**	**No Analyte**	**Streptavidin (0.05 mg/mL)**	**UEA (0.05 mg/mL)**
**Biotin-PHEA53AuNP16**	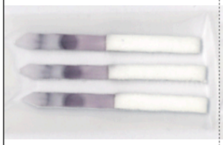	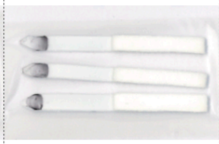	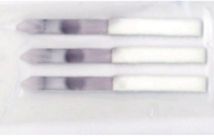
**Biotin-PHEA72AuNP16**	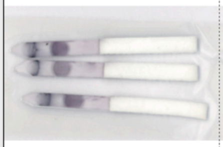	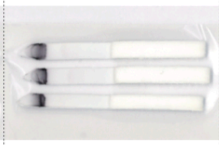	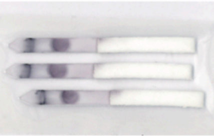
**Biotin-PHEA110AuNP16**	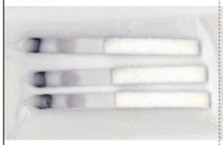	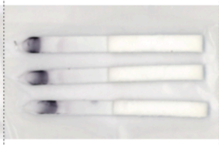	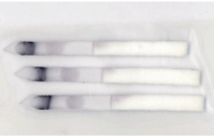

Table S7. Scans of lateral flow strips using a test line of PVP_{50}-Biotin (10 mg/mL) versus no analyte, streptavidin (0.05 mg/mL) and UEA (0.05 mg/mL), using Biotin-$PHEA_{53}$@$AuNP_{16}$, Biotin-$PHEA_{72}$@$AuNP_{16}$ and Biotin-$PHEA_{110}$@$AuNP_{16}$

PVP80-Biotin Test line (10 mg/mL)	No Analyte	Streptavidin (0.05 mg/mL)	UEA (0.05 mg/mL)
**Biotin-PHEA53AuNP16**	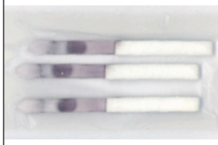	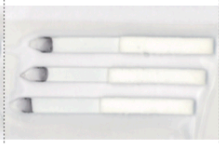	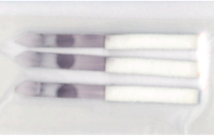
**Biotin-PHEA72AuNP16**	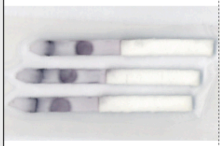	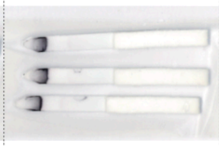	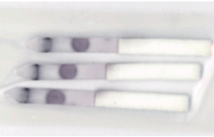
**Biotin-PHEA110AuNP16**	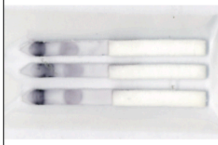	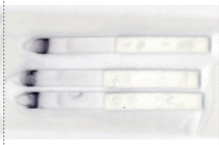	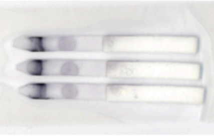

Table S8. Scans of lateral flow strips using a test line of PVP_{80}-Biotin (10 mg/mL) versus no analyte, streptavidin (0.05 mg/mL) and UEA (0.05 mg/mL), using Biotin-$PHEA_{53}$@$AuNP_{16}$, Biotin-$PHEA_{72}$@$AuNP_{16}$ and Biotin-$PHEA_{110}$@$AuNP_{16}$

PVP150-Biotin Test line (10 mg/mL)	No Analyte	Streptavidin (0.05 mg/mL)	UEA (0.05 mg/mL)
**Biotin-PHEA53AuNP16**	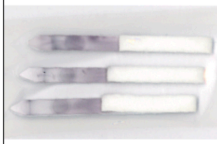	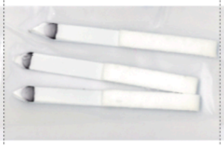	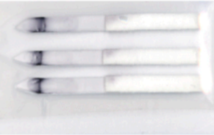
**Biotin-PHEA72AuNP16**	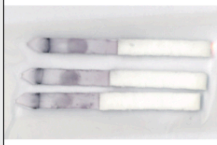	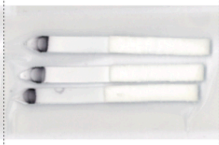	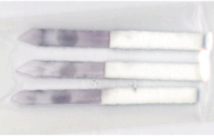
**Biotin-PHEA110AuNP16**	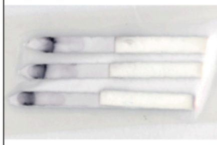	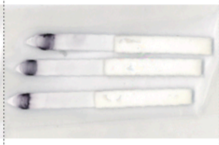	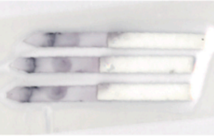

Table S9. Scans of lateral flow strips using a test line of PVP_{150}-Biotin (10 mg/mL) versus no analyte, streptavidin (0.05 mg/mL) and UEA (0.05 mg/mL), using Biotin-$PHEA_{53}$@$AuNP_{16}$, Biotin-$PHEA_{72}$@$AuNP_{16}$ and Biotin-$PHEA_{110}$@$AuNP_{16}$

PVP50-Biotin Test line (10 mg/mL)	No Analyte	Streptavidin (0.05 mg/mL)	UEA (0.05 mg/mL)
**Biotin-PHEA72AuNP40**	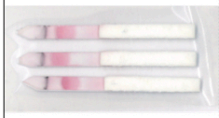	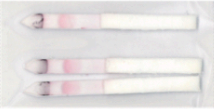	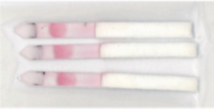
**Biotin-PHEA110AuNP40**	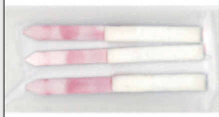	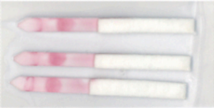	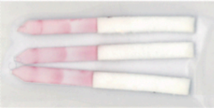

Table S10. Scans of lateral flow strips using a test line of PVP_{50}-Biotin (10 mg/mL) versus no analyte, streptavidin (0.05 mg/mL) and UEA (0.05 mg/mL), using Biotin-$PHEA_{72}$@$AuNP_{40}$ and Biotin-$PHEA_{110}$@$AuNP_{40}$

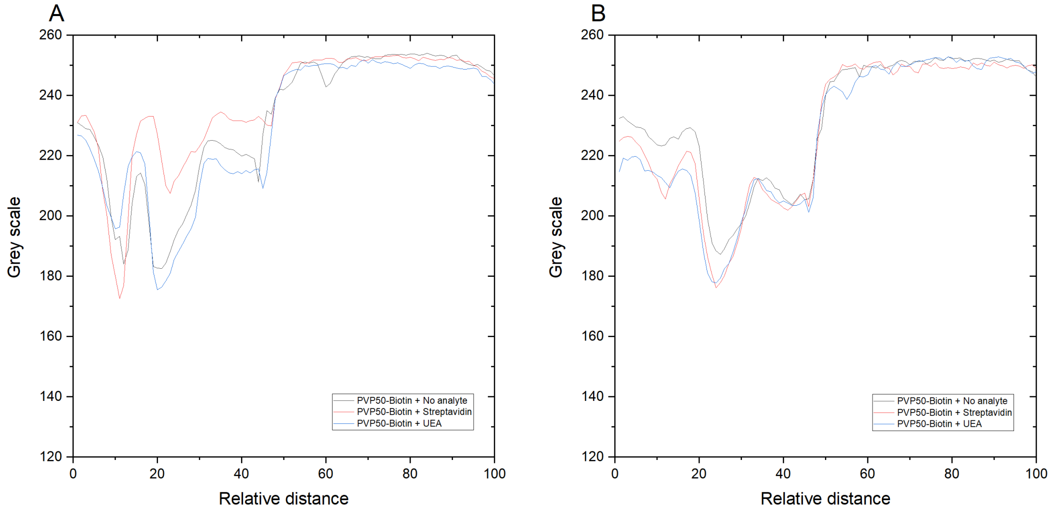

Figure S26. Analysis of scanned lateral flow strips using test lines of PVP_{50}-Biotin (10 mg/mL) versus either no analyte, Streptavidin (0.05 mg/mL) and UEA (0.05 mg/mL), using A) Biotin-$PHEA_{72}$@$AuNP_{40}$ or B) Biotin-$PHEA_{110}$@$AuNP_{40}$

PVP80-Biotin Test line (10 mg/mL)	No Analyte	Streptavidin (0.05 mg/mL)	UEA (0.05 mg/mL)
**Biotin-PHEA72AuNP40**	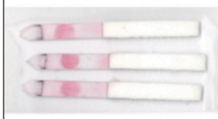	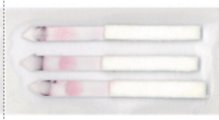	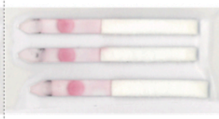
**Biotin-PHEA110AuNP40**	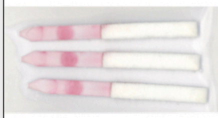	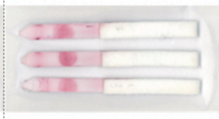	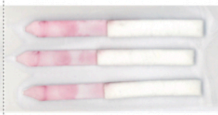

Table S11. Scans of lateral flow strips using a test line of PVP_{80}-Biotin (10 mg/mL) versus no analyte, streptavidin (0.05 mg/mL) and UEA (0.05 mg/mL), using Biotin-$PHEA_{72}$@$AuNP_{40}$ and Biotin-$PHEA_{110}$@$AuNP_{40}$

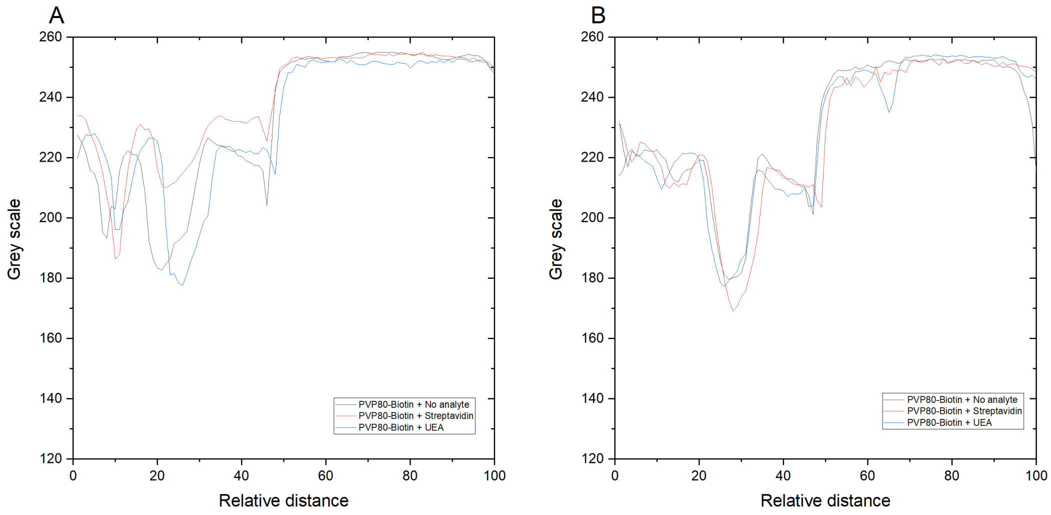

Figure S27. Analysis of scanned lateral flow strips using test lines of PVP_{80}-Biotin (10 mg/mL) versus either no analyte, Streptavidin (0.05 mg/mL) and UEA (0.05 mg/mL), using A) Biotin-$PHEA_{72}$@$AuNP_{40}$ or B) Biotin-$PHEA_{110}$@$AuNP_{40}$

PVP150-Biotin Test line (10 mg/mL)	No Analyte	Streptavidin (0.05 mg/mL)	UEA (0.05 mg/mL)
Biotin-PHEA72AuNP40	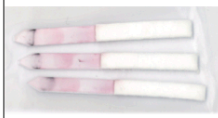	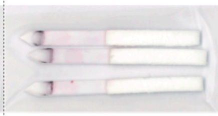	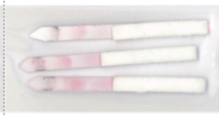
Biotin-PHEA110AuNP40	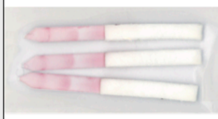	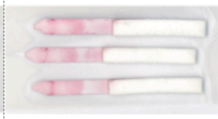	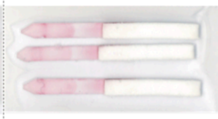

Table S12. Scans of lateral flow strips using a test line of PVP_{150}-Biotin (10 mg/mL) versus no analyte, streptavidin (0.05 mg/mL) and UEA (0.05 mg/mL), using Biotin-$PHEA_{72}$@$AuNP_{40}$ and Biotin-$PHEA_{110}$@$AuNP_{40}$

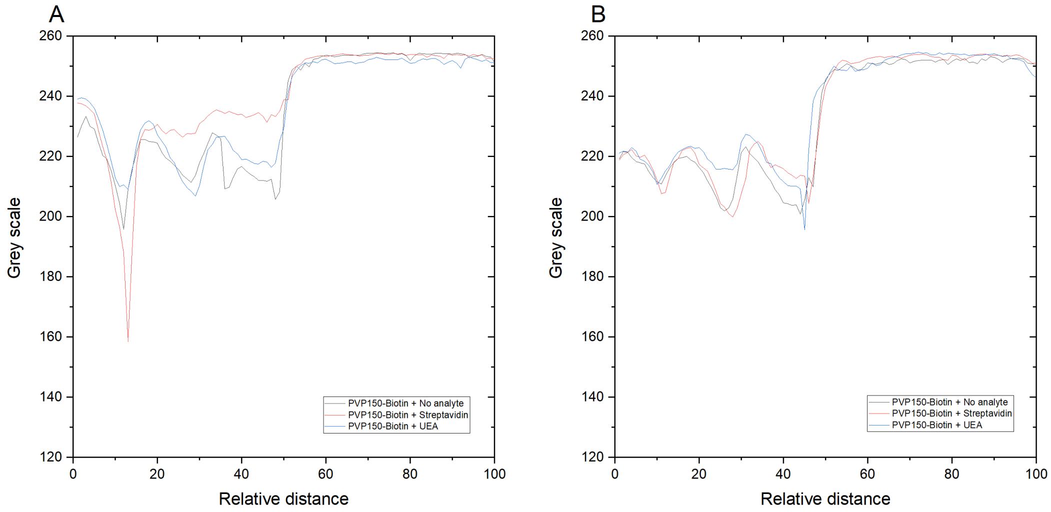

Figure S28. Analysis of scanned lateral flow strips using test lines of PVP_{150}-Biotin (10 mg/mL) versus either no analyte, Streptavidin (0.05 mg/mL) and UEA (0.05 mg/mL), using A) Biotin-$PHEA_{72}$@$AuNP_{40}$ or B) Biotin-$PHEA_{110}$@$AuNP_{40}$

PVP Test line (1 mg/mL)	No Analyte	Streptavidin (0.05 mg/mL)	UEA (0.05 mg/mL)
PVP50-Biotin	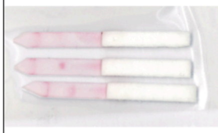	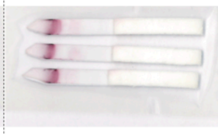	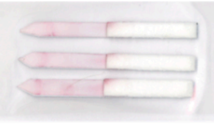
PVP80-Biotin	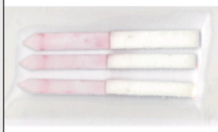	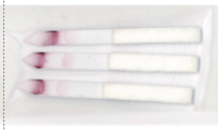	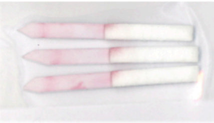
PVP150-Biotin	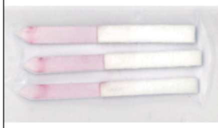	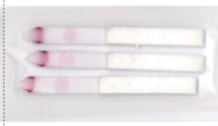	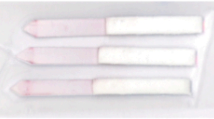

Table S13. Scans of lateral flow strips using test lines of PVP-Biotin (1 mg/mL) versus no analyte, streptavidin (0.05 mg/mL) and UEA (0.05 mg/mL), using Biotin-PHEA$_{110}$@AuNP$_{40}$

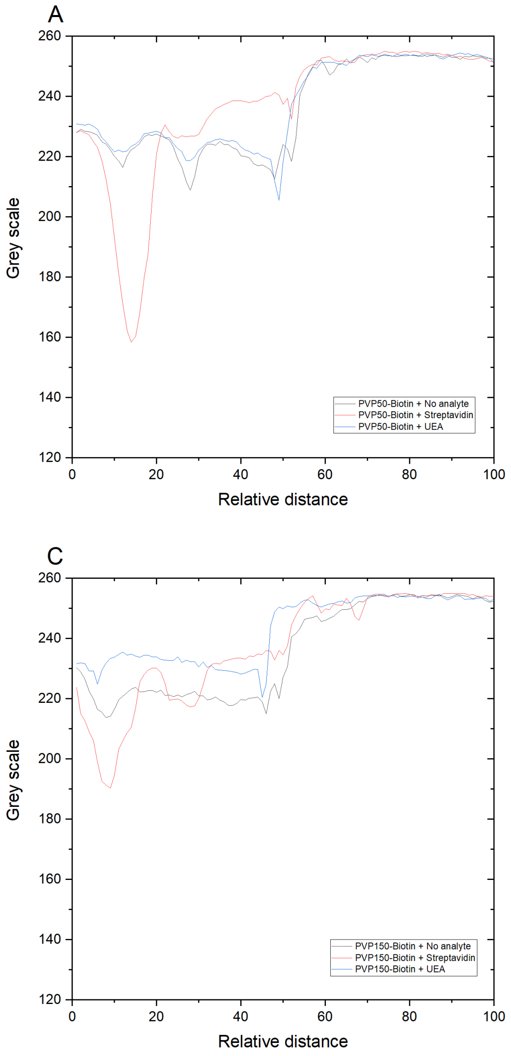

Figure S29. Analysis of scanned lateral flow strips using test lines of PVP-Biotin (1 mg/mL) versus either no analyte, Streptavidin (0.05 mg/mL) and UEA (0.05 mg/mL), using Biotin-PHEA$_{110}$@AuNP$_{40}$. Tests lines used are A) PVP$_{50}$-Biotin, B) PVP$_{80}$-Biotin and C) PVP$_{150}$-Biotin

PVP Test line (1 mg/mL)	Streptavidin (0.05 mg/mL)
**PVP50-Biotin**	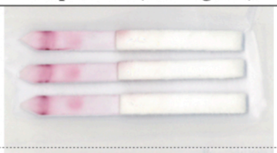
**PVP80-Biotin**	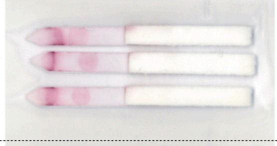
**PVP150-Biotin**	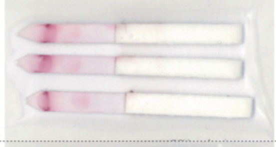
**PVP50**	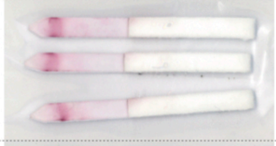
**PVP80**	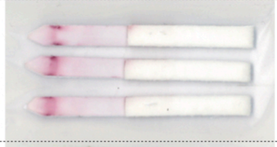
**PVP150**	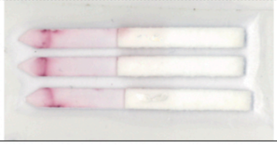

Table S14. Scans of lateral flow strips using test lines of PVP and PVP-Biotin (1 mg/mL) versus Streptavidin (0.05 mg/mL), using Biotin-PHEA$_{110}$@AuNP$_{40}$

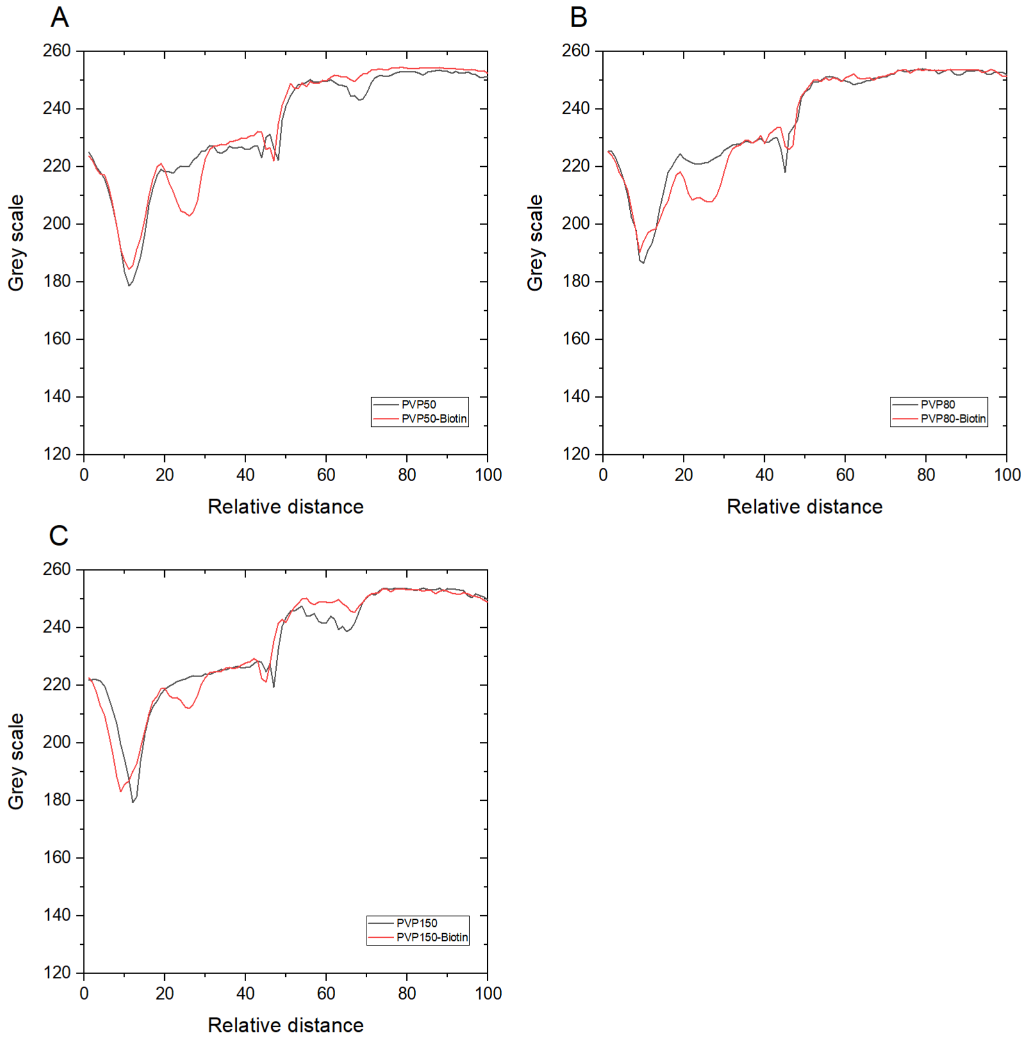

Figure S30. Analysis of scanned lateral flow strips using test lines of PVP and PVP-Biotin (1 mg/mL) versus Streptavidin (0.05 mg/mL) using Biotin-PHEA$_{110}$@AuNP$_{40}$. Tests lines used are A) PVP$_{50}$ & PVP$_{50}$-Biotin, B) PVP$_{80}$ & PVP$_{80}$-Biotin and C) PVP$_{150}$ & PVP$_{150}$-Biotin

PVP Test line (1 mg/mL)	Streptavidin (0.005 mg/mL)
**PVP50-Biotin**	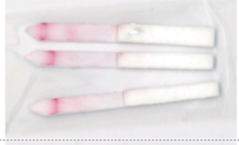
**PVP80-Biotin**	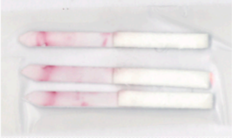
**PVP150-Biotin**	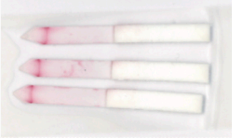
**PVP50**	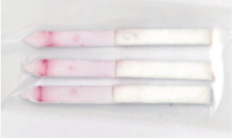
**PVP80**	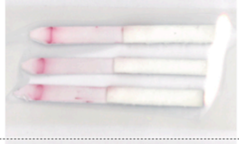
**PVP150**	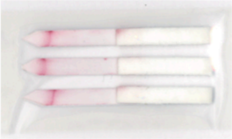

Table S15. Scans of lateral flow strips using test lines of PVP and PVP-Biotin (1 mg/mL) versus Streptavidin (0.005 mg/mL), using Biotin-$PHEA_{110}$@$AuNP_{40}$

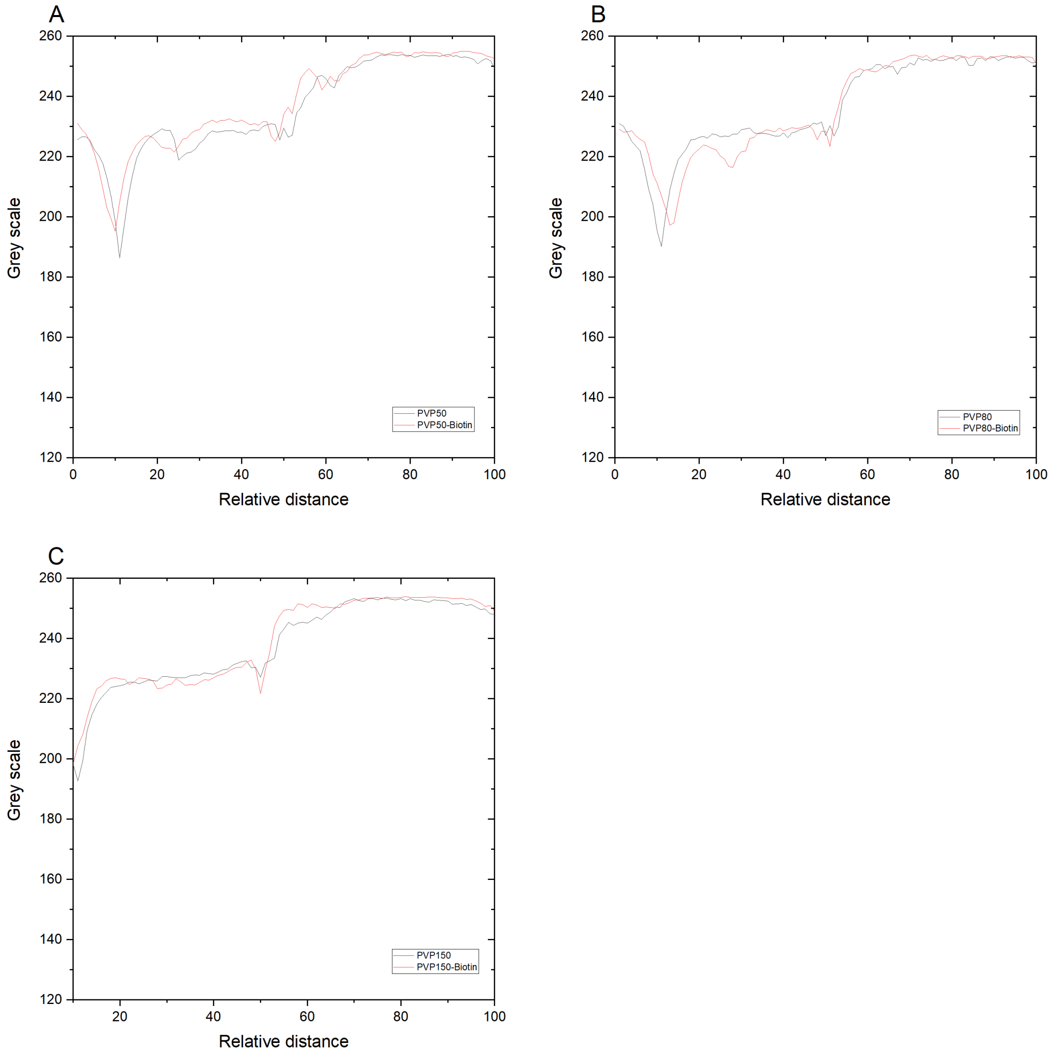

Figure S31. Analysis of scanned lateral flow strips using test lines of PVP and PVP-Biotin (1 mg/mL) versus Streptavidin (0.005 mg/mL) using Biotin-$PHEA_{110}$@$AuNP_{40}$. Tests lines used are A) PVP_{50} & PVP_{50}-Biotin, B) PVP_{80} & PVP_{80}-Biotin and C) PVP_{150} & PVP_{150}-Biotin

Lateral Flow Dipsticks and Analysis – Galactosamine and SBA

PVP Test line (20 mg/mL)	No Lectin	SBA (0.05 mg/mL)	UEA (0.05 mg/mL)
PVP50-Gal	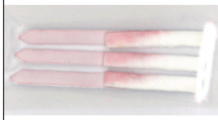	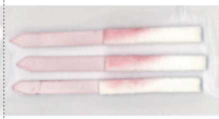	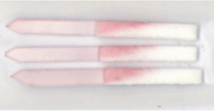
PVP80-Gal	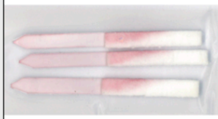	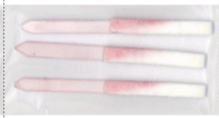	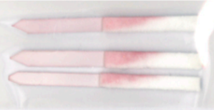
PVP150-Gal	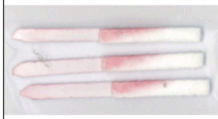	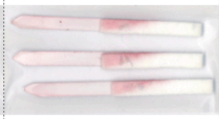	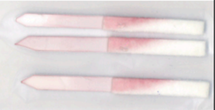
PVP50	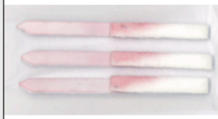	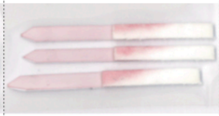	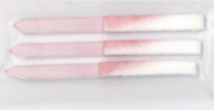
PVP80	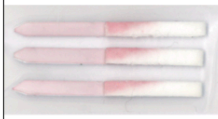	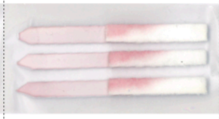	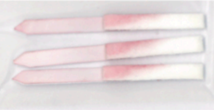
PVP150	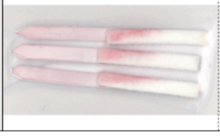	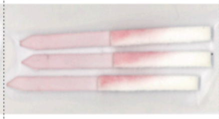	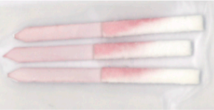

Table S16. Scans of lateral flow strips using test lines of PVP and PVP-Gal (20 mg/mL) versus no lectin, SBA (0.05 mg/mL) and UEA (0.05 mg/mL) using Gal-$PHEA_{72}$@$AuNP_{16}$

PVP Test line (20 mg/mL)	No Lectin	SBA (0.5 mg/mL)	UEA (0.5 mg/mL)
**PVP50-Gal**	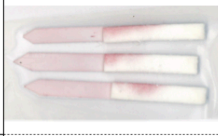	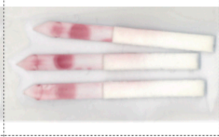	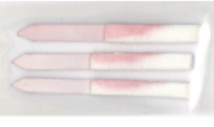
**PVP80-Gal**	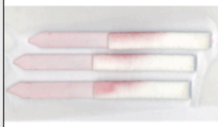	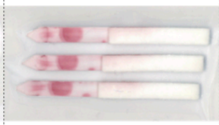	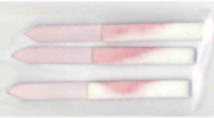
**PVP150-Gal**	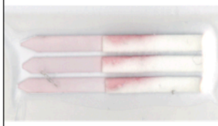	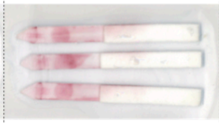	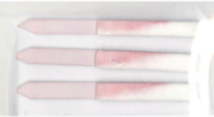
**PVP50**	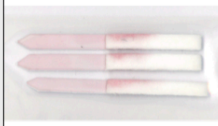	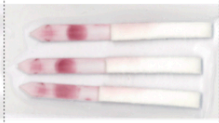	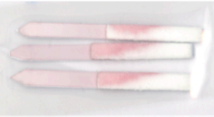
**PVP80**	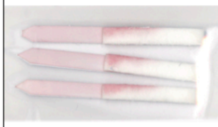	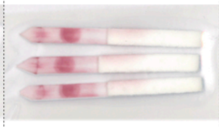	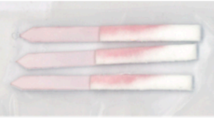
**PVP150**	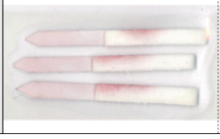	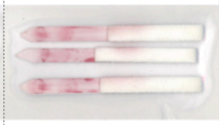	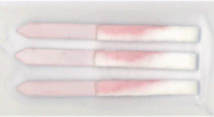

Table S17. Scans of lateral flow strips using test lines of PVP and PVP-Gal (20 mg/mL) versus no lectin, SBA (0.5 mg/mL) and UEA (0.5 mg/mL) using Gal-PHEA$_{72}$@AuNP$_{16}$

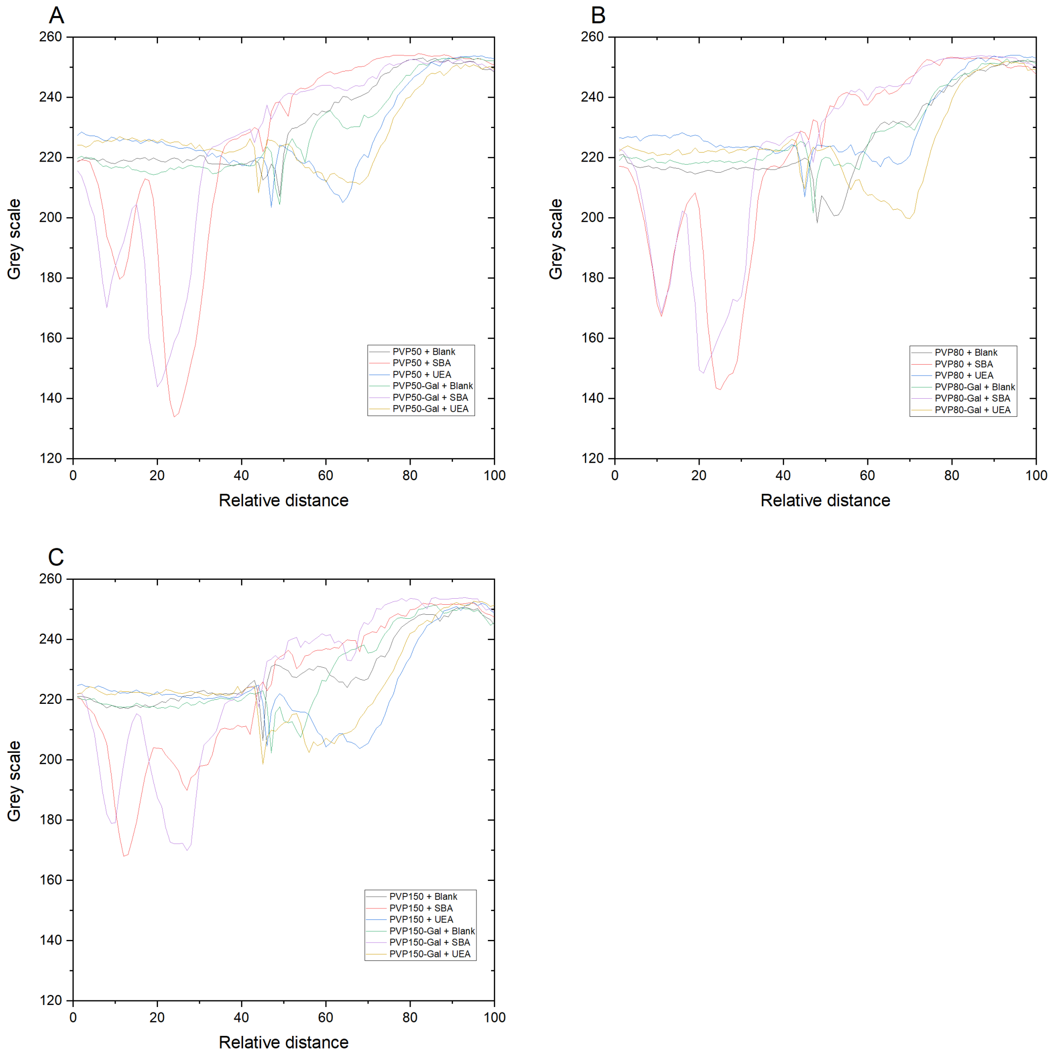

Figure S32. Analysis of scanned lateral flow strips using test lines of PVP and PVP-Gal (20 mg/mL) versus either no analyte, SBA (0.5 mg/mL) and UEA (0.5 mg/mL), using Gal-$PHEA_{72}$@$AuNP_{16}$. Tests lines used are A) PVP_{50} & PVP_{50}-Gal, B) PVP_{80} & PVP_{80}-Gal and C) PVP_{150} & PVP_{150}-Gal

PVP Test line (10 mg/mL)	No Lectin	SBA (0.5 mg/mL)	UEA (0.5 mg/mL)
**PVP50-Gal**	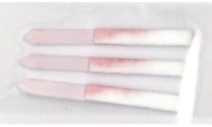	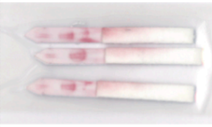	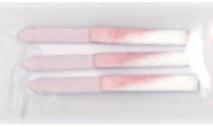
**PVP80-Gal**	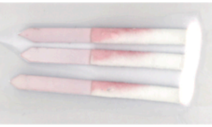	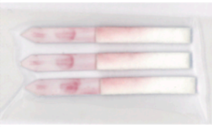	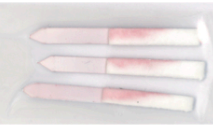
**PVP150-Gal**	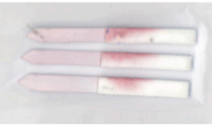	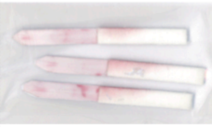	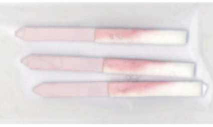
**PVP50**	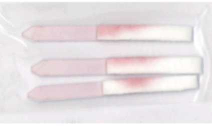	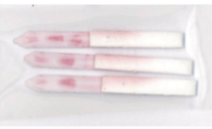	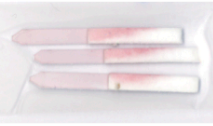
**PVP80**	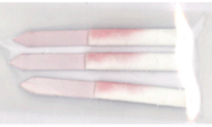	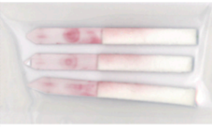	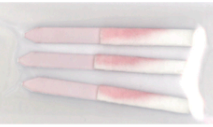
**PVP150**	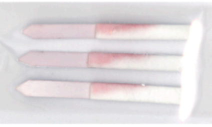	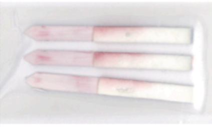	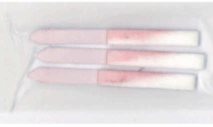

Table S18. Scans of lateral flow strips using test lines of PVP and PVP-Gal (10 mg/mL) versus no lectin, SBA (0.5 mg/mL) and UEA (0.5 mg/mL) using Gal-PHEA$_{72}$@AuNP$_{16}$

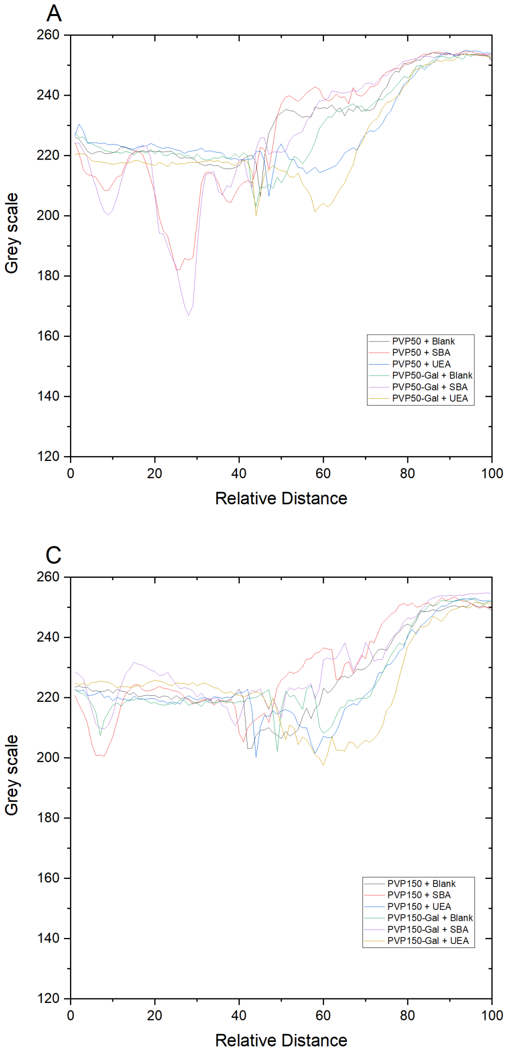

Figure S33. Analysis of scanned lateral flow strips using test lines of PVP and PVP-Gal (10 mg/mL) versus either no analyte, SBA (0.5 mg/mL) and UEA (0.5 mg/mL), using Gal-PHEA$_{72}$@AuNP$_{16}$. Tests lines used are A) PVP$_{50}$ & PVP$_{50}$-Gal, B) PVP$_{80}$ & PVP$_{80}$-Gal and C) PVP$_{150}$ & PVP$_{150}$-Gal.

PVP Test line (1 mg/mL)	No Lectin	SBA (0.5 mg/mL)	UEA (0.5 mg/mL)
PVP50-Gal	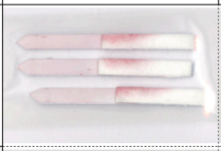	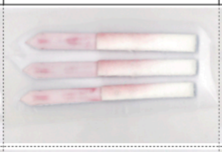	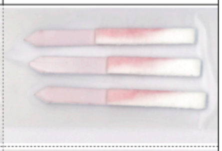
PVP80-Gal	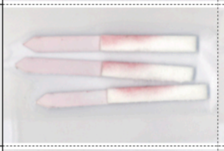	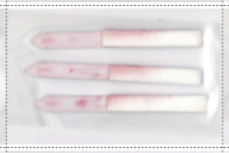	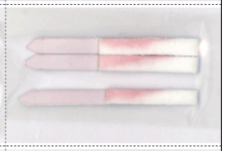
PVP150-Gal	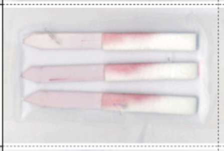	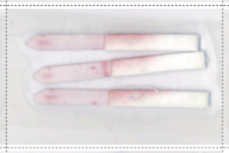	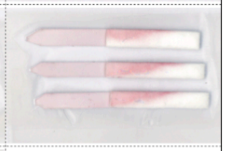
PVP50	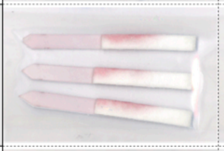	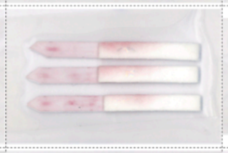	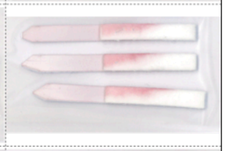
PVP80	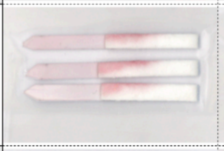	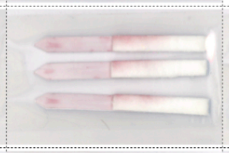	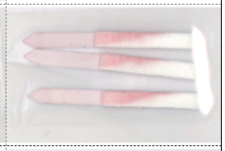
PVP150	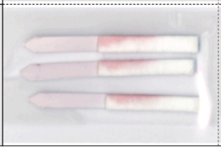	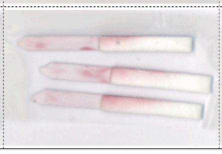	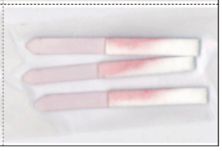

Table S19. Scans of lateral flow strips using test lines of PVP and PVP-Gal (1 mg/mL) versus no lectin, SBA (0.5 mg/mL) and UEA (0.5 mg/mL) using Gal-$PHEA_{72}$@$AuNP_{16}$

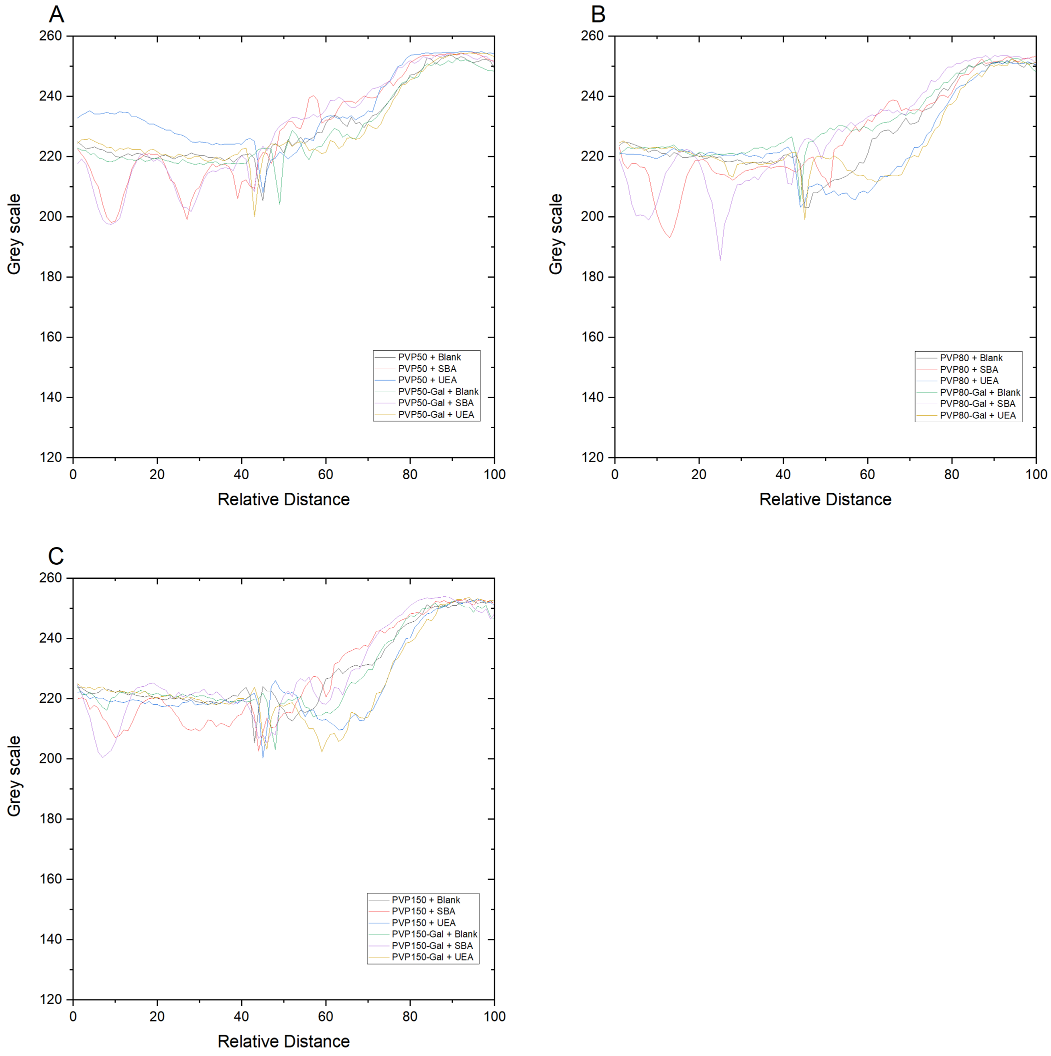

Figure S34. Analysis of scanned lateral flow strips using test lines of PVP and PVP-Gal (1 mg/mL) versus either no analyte, SBA (0.5 mg/mL) and UEA (0.5 mg/mL), using Gal-$PHEA_{72}$@$AuNP_{16}$. Tests lines used are A) PVP_{50} & PVP_{50}-Gal, B) PVP_{80} & PVP_{80}-Gal and C) PVP_{150} & PVP_{150}-Gal.

XPS Data

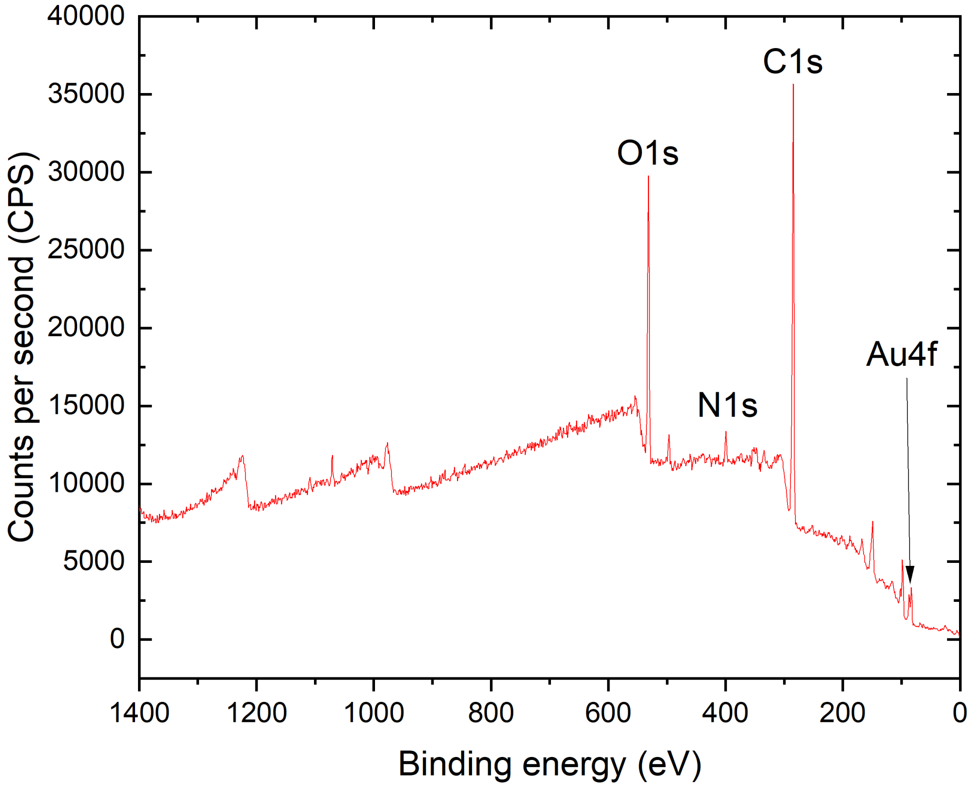

Figure S35. Representative XPS survey scan of Biotin-PHEA$_{72}$@AuNP$_{40}$

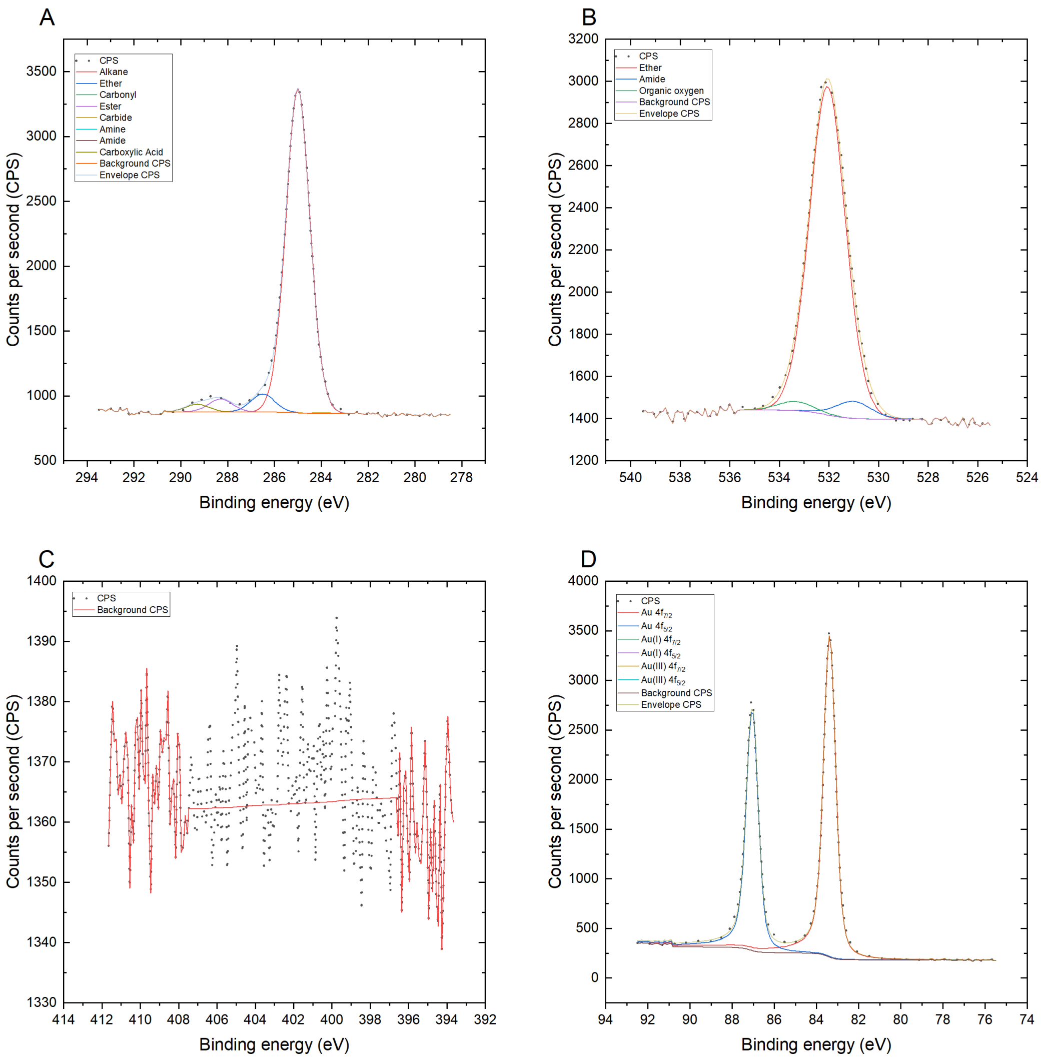

Figure S36. XPS of naked 16 nm AuNP A) C 1*s* B) O 1*s* C) N 1*s* and D) Au 4*f*

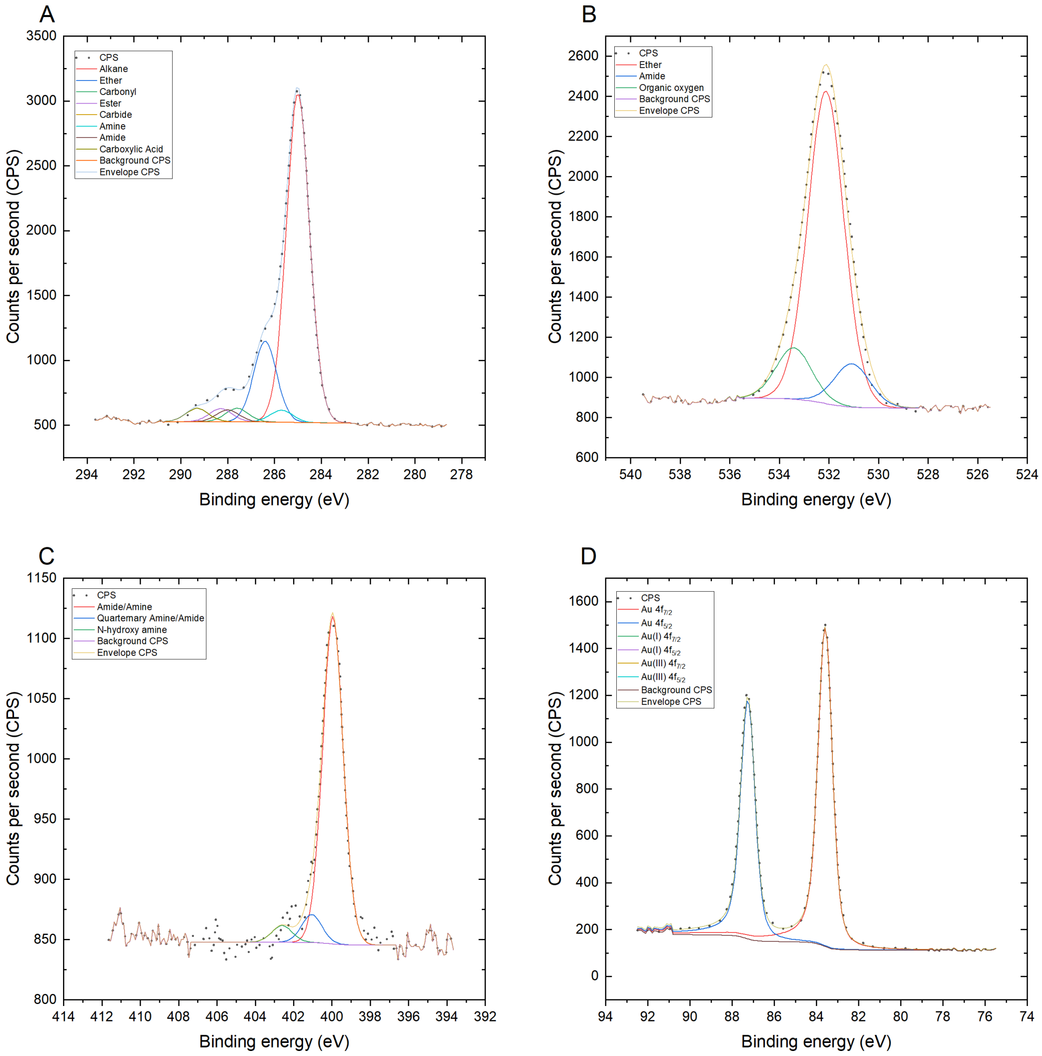

Figure S37. XPS of Gal-PHEA$_{72}$@AuNP$_{16}$ A) C 1*s* B) O 1*s* C) N 1*s* and D) Au 4*f*

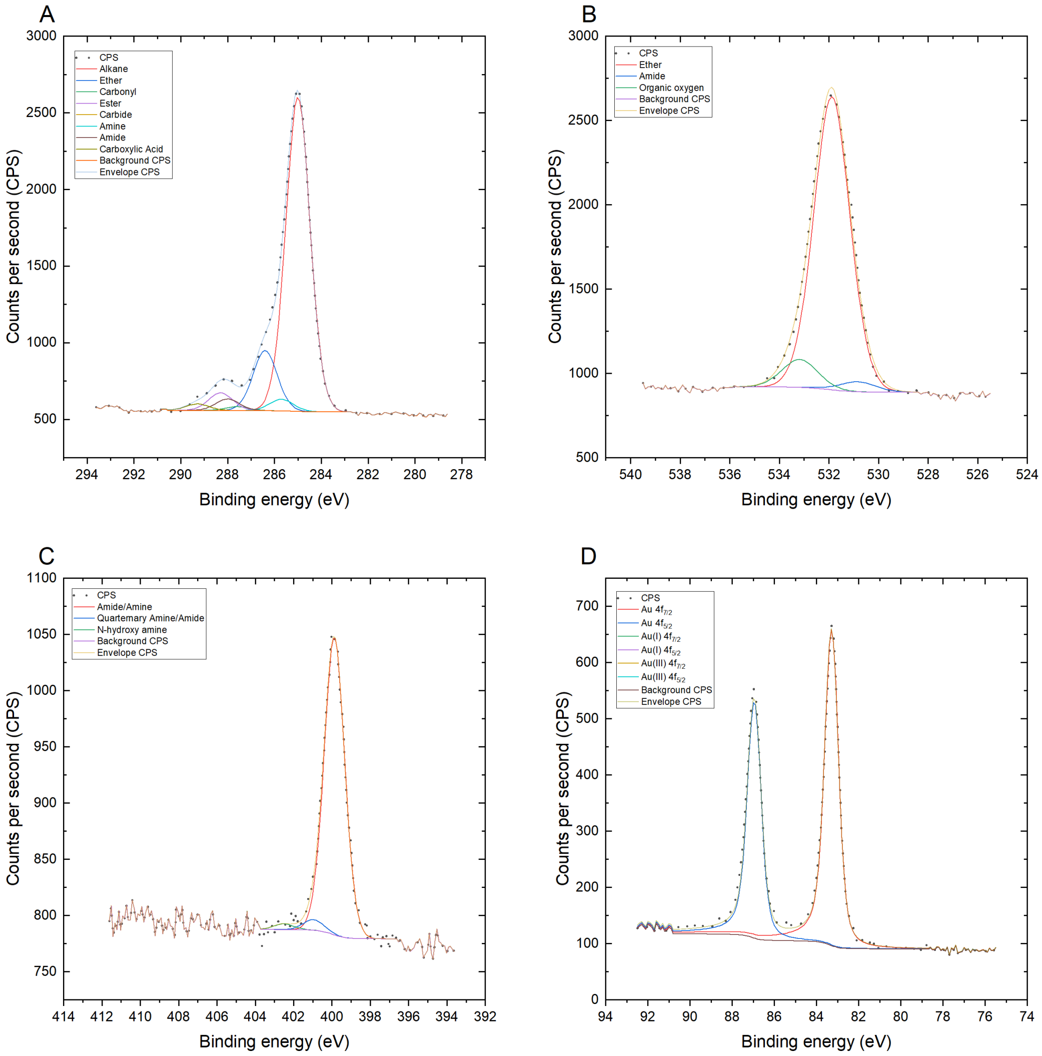

Figure S38. XPS of Biotin-PHEA$_{53}$@AuNP$_{16}$ A) C 1*s* B) O 1*s* C) N 1*s* and D) Au 4*f*

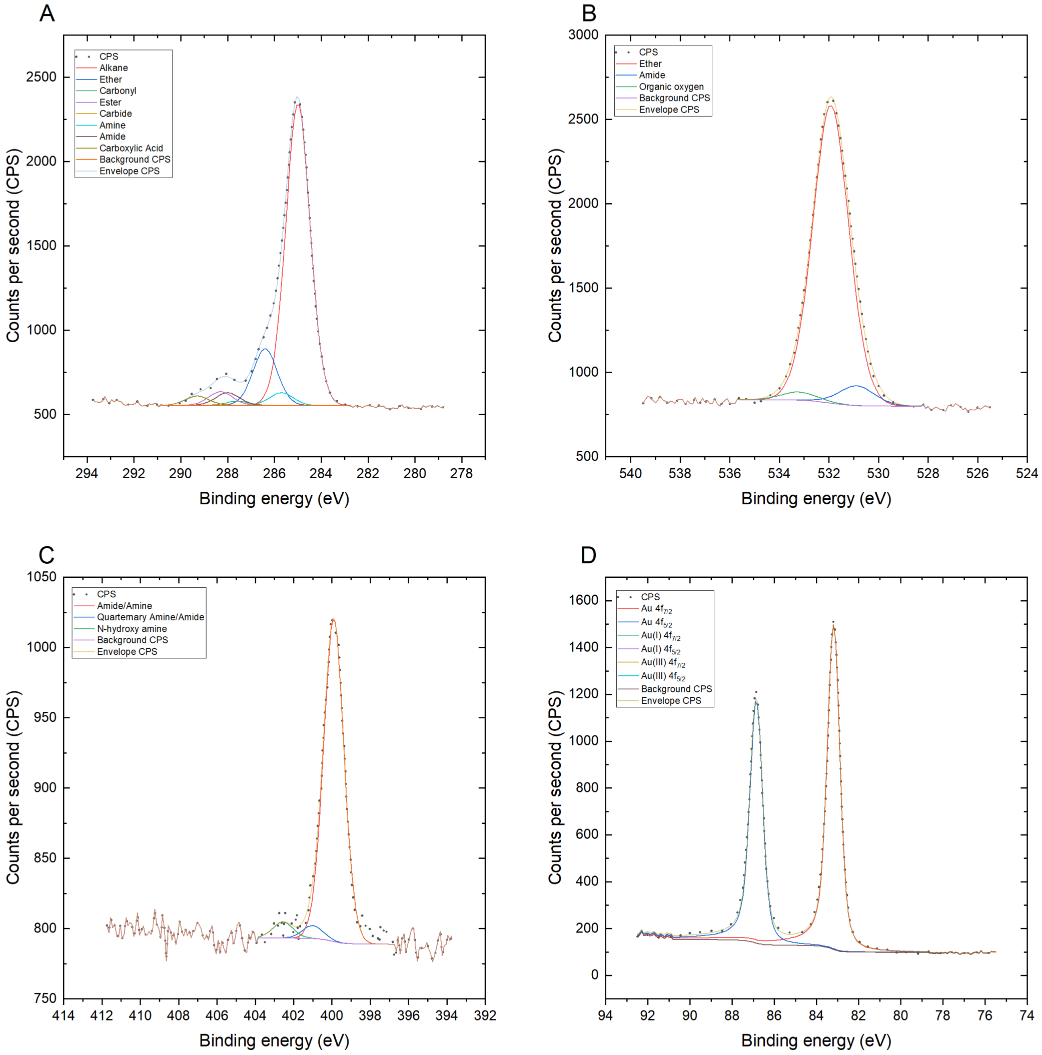

Figure S39. XPS of Biotin-PHEA$_{72}$@AuNP$_{16}$ A) C 1*s* B) O 1*s* C) N 1*s* and D) Au 4*f*

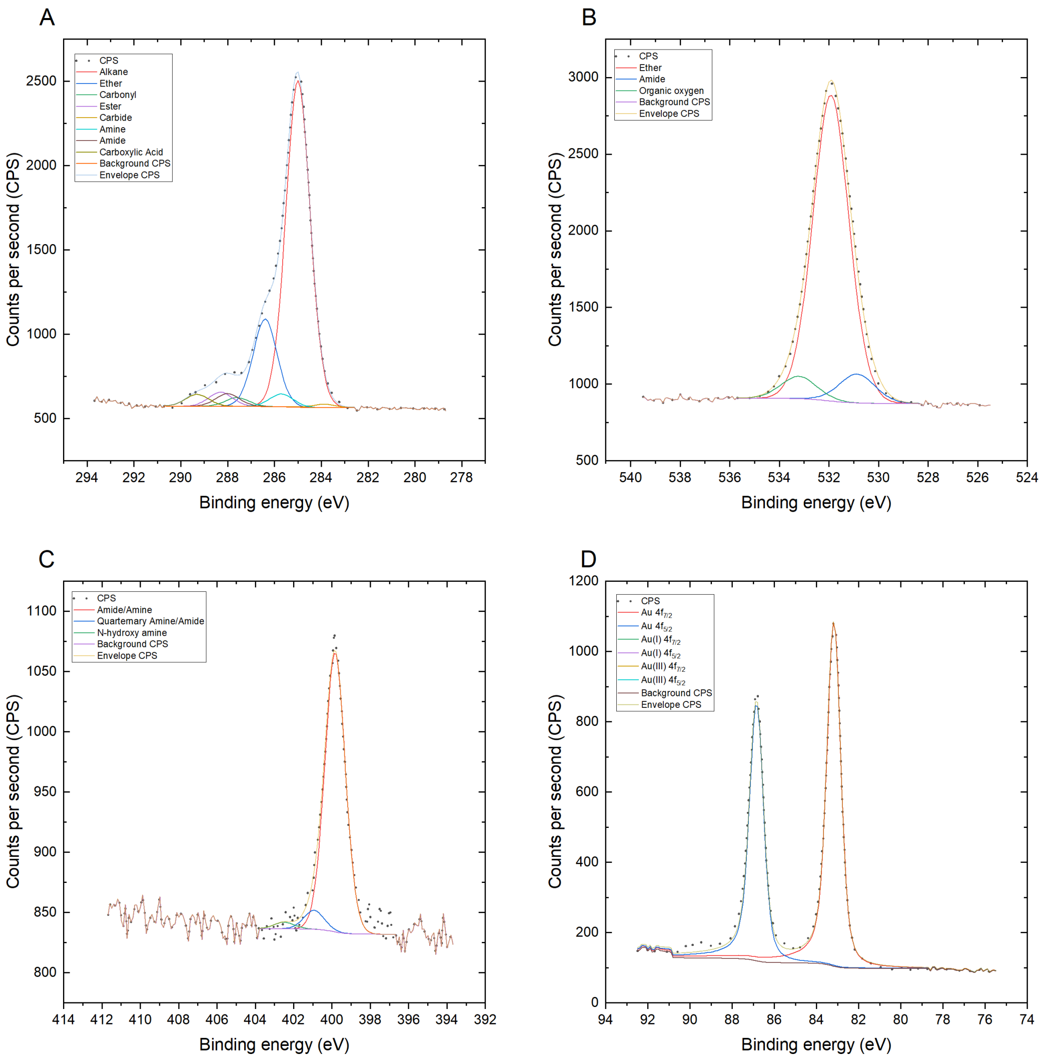

Figure S40. XPS of Biotin-PHEA$_{110}$@AuNP$_{16}$ A) C 1*s* B) O 1*s* C) N 1*s* and D) Au 4*f*

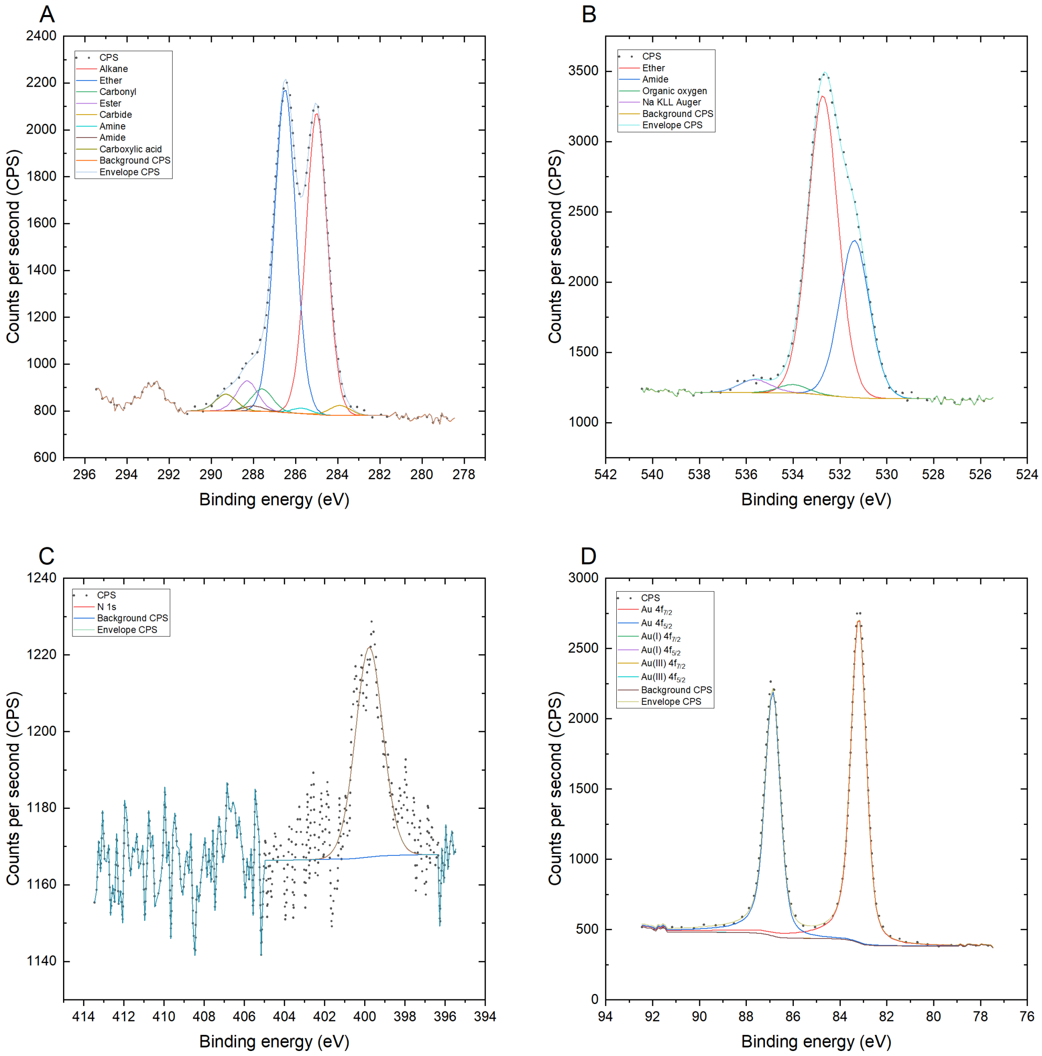

Figure S41. XPS of naked 40 nm AuNP A) C 1*s* B) O 1*s* C) N 1*s* and D) Au 4*f*

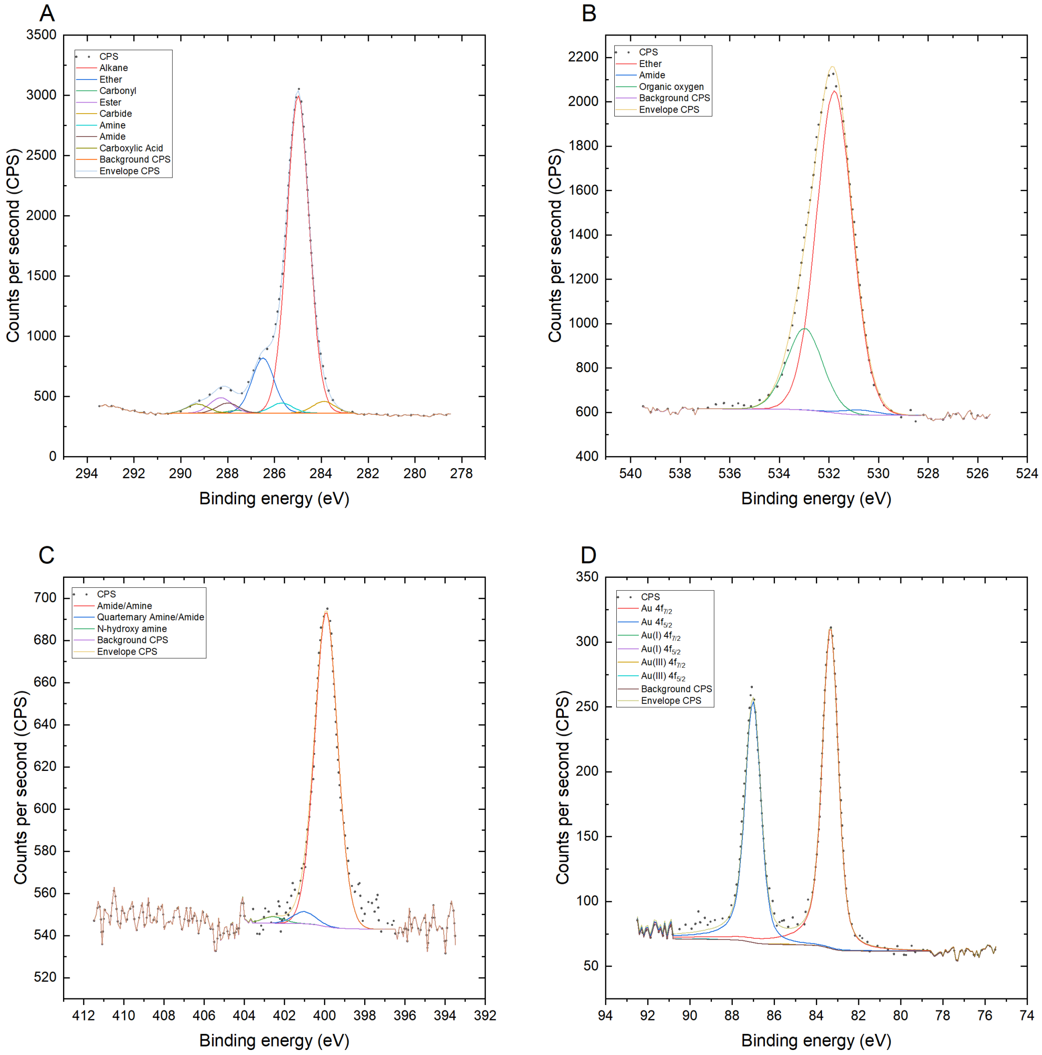

Figure S42. XPS of Biotin-PHEA$_{72}$@AuNP$_{40}$ A) C 1*s* B) O 1*s* C) N 1*s* and D) Au 4*f*

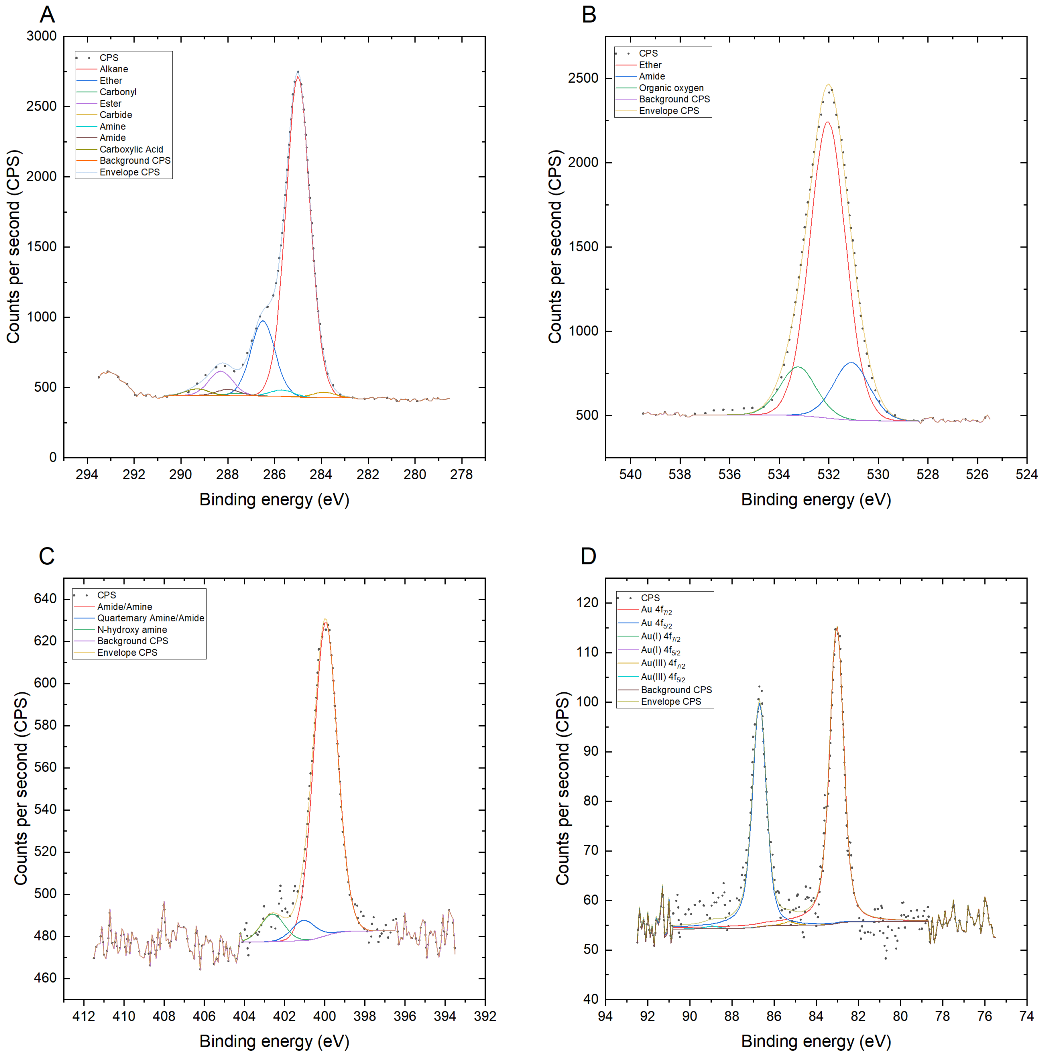

Figure S43. XPS of Biotin-PHEA$_{110}$@AuNP$_{40}$ A) C 1*s* B) O 1*s* C) N 1*s* and D) Au 4*f*

Particle Composition	Elemental Percentage Composition (%)				Elemental Ratios	
	C 1*s*	O 1*s*	N 1*s*	Au 4*f*	N 1*s*/ C 1*s*	N 1*s*/ Au 4*f*
Naked 16 nm	73.3	20.3	0.5	6.0	0.01	0.08
Gal-$PHEA_{72}$@$AuNP_{16}$	74.1	19.7	3.9	2.4	0.05	1.62
Biotin-$PHEA_{53}$@$AuNP_{16}$	71.5	23.2	4.1	1.2	0.06	3.50
Biotin-$PHEA_{72}$@$AuNP_{16}$	68.3	24.8	4.1	2.8	0.06	1.44
Biotin-$PHEA_{110}$@$AuNP_{16}$	68.5	25.9	3.6	1.9	0.05	1.88
Naked 40 nm	61.7	33.9	1.0	3.4	0.02	0.30
Biotin-$PHEA_{72}$@$AuNP_{40}$	77.2	19.8	2.4	0.5	0.03	4.43
Biotin-$PHEA_{110}$@$AuNP_{40}$	72.3	25.1	2.5	0.1	0.03	22.46

Table S20. Elemental composition of nanoparticles determined by XPS

Particle Composition	C 1*s* Bond Percentage Composition (%)								Bond Ratios	
	Alkane	Ether	Carbonyl	Ester	Carbide	Amine	Amide	Carboxylic Acid	Amide/Alkane	Amide/Ether
Naked 16 nm	89.0	5.0	0.0	3.6	0.2	0.0	0.0	2.2	0.00	0.00
Gal-$PHEA_{72}$@$AuNP_{16}$	69.3	17.1	2.9	2.8	0.0	2.5	2.5	2.9	0.04	0.15
Biotin-$PHEA_{53}$@$AuNP_{16}$	73.7	14.2	0.9	4.2	0.0	2.8	2.8	1.6	0.04	0.19
Biotin-$PHEA_{72}$@$AuNP_{16}$	73.3	13.8	1.0	3.4	0.0	3.1	3.1	2.3	0.04	0.23
Biotin-$PHEA_{110}$@$AuNP_{16}$	68.3	18.3	1.9	3.0	0.7	2.7	2.7	2.5	0.04	0.15
Naked 40 nm	42.2	45.2	3.2	4.2	1.4	0.8	0.8	2.36	0.02	0.02
Biotin-$PHEA_{72}$@$AuNP_{40}$	73.3	12.8	0.7	3.6	2.7	2.4	2.4	2.1	0.03	0.19
Biotin-$PHEA_{110}$@$AuNP_{40}$	71.5	16.8	0.6	5.5	1.2	1.4	1.4	1.5	0.02	0.09

Table S21. C 1*s* bonding composition of nanoparticles determined by XPS

References

(1) Schneider, C. A.; Rasband, W. S.; Eliceiri, K. W. NIH Image to ImageJ: 25 Years of Image Analysis. *Nat. Methods* **2012**, *9* (7), 671–675.

(2) Richards, S.-J.; Gibson, M. I. Optimization of the Polymer Coating for Glycosylated Gold Nanoparticle Biosensors to Ensure Stability and Rapid Optical Readouts. *ACS Macro Lett.* **2014**, *3* (10), 1004–1008.

(3) Eisenführ, A.; Arora, P. S.; Sengle, G.; Takaoka, L. R.; Nowick, J. S.; Famulok, M. A Ribozyme with Michaelase Activity. *Bioorg. Med. Chem.* **2003**, *11* (2), 235–249.

(4) Kaufman, N. E. M.; Meng, Q.; Griffin, K. E.; Singh, S. S.; Dahal, A.; Zhou, Z.; Fronczek, F. R.; Mathis, J. M.; Jois, S. D.; Vicente, M. G. H. Synthesis, Characterization, and Evaluation of Near-IR Boron Dipyrromethene Bioconjugates for Labeling of Adenocarcinomas by Selectively Targeting the Epidermal Growth Factor Receptor. *J. Med. Chem.* **2019**, *62* (7), 3323–3335.

(5) Ieong, N. S.; Brebis, K.; Daniel, L. E.; O'Reilly, R. K.; Gibson, M. I. The Critical Importance of Size on Thermoresponsive Nanoparticle Transition Temperatures: Gold and Micelle-Based Polymer Nanoparticles. *Chem. Commun.* **2011**, *47* (42), 11627–11629.

(6) Baker, A. N.; Richards, S.-J. J.; Guy, C. S.; Congdon, T. R.; Hasan, M.; Zwetsloot, A. J.; Gallo, A.; Lewandowski, J. R.; Stansfeld, P. J.; Straube, A.; Walker, M.; Chessa, S.; Pergolizzi, G.; Dedola, S.; Field, R. A.; Gibson, M. I. The SARS-COV-2 Spike Protein Binds Sialic Acids and Enables Rapid Detection in a Lateral Flow Point of Care Diagnostic Device. *ACS Cent. Sci.* **2020**, *6* (11), 2046–2052.
